# The chromosome-level wintersweet (*Chimonanthus praecox*) genome provides insights into floral scent biosynthesis and flowering in winter

**DOI:** 10.1186/s13059-020-02088-y

**Published:** 2020-08-10

**Authors:** Junzhong Shang, Jingpu Tian, Huihui Cheng, Qiaomu Yan, Lai Li, Abbas Jamal, Zhongping Xu, Lin Xiang, Christopher A. Saski, Shuangxia Jin, Kaige Zhao, Xiuqun Liu, Longqing Chen

**Affiliations:** 1 Key Laboratory of Horticultural Plant Biology, Ministry of Education, Huazhong Agricultural University, Wuhan, Hubei 430070 People’s Republic of China; 2 Novogene Bioinformatics Institute, Beijing, 100083 People’s Republic of China; 3 National Key Laboratory of Crop Genetic Improvement, Huazhong Agricultural University, Wuhan, Hubei 430070 People’s Republic of China; 4 Xinjiang Production and Construction Corps Key Laboratory of Protection and Utilization of Biological Resources in Tarim Basin, Tarim University, Alaer, Xinjiang, 843300 China; 5 Department of Plant, Clemson University, Clemson, SC 29631 USA; 6 Southwest Engineering Technology and Research Center of Landscape Architecture, State Forestry Administration, Southwest Forestry University, Kunming, Yunnan 650224 People’s Republic of China

**Keywords:** Wintersweet (*Chimonanthus praecox*), Hi-C, Genome, Floral scent, Flowering, Cold tolerance

## Abstract

**Background:**

Wintersweet (*Chimonanthus praecox*), an important ornamental plant, has evolved unique fragrant aroma and winter-flowering properties, which are critical for its successful sexual reproduction. However, the molecular mechanisms underlying these traits are largely unknown in this species. In addition, wintersweet is also a typical representative species of the magnoliids, where the phylogenetic position of which relative to eudicots and monocots has not been conclusively resolved.

**Results:**

Here, we present a chromosome-level wintersweet genome assembly with a total size of 695.36 Mb and a draft genome assembly of *Calycanthus chinensis*. Phylogenetic analyses of 17 representative angiosperm genomes suggest that Magnoliids and eudicots are sister to monocots. Whole-genome duplication signatures reveal two major duplication events in the evolutionary history of the wintersweet genome, with an ancient one shared by Laurales, and a more recent one shared by the Calycantaceae. Whole-genome duplication and tandem duplication events have significant impacts on copy numbers of genes related to terpene and benzenoid/phenylpropanoid (the main floral scent volatiles) biosynthesis, which may contribute to the characteristic aroma formation. An integrative analysis combining cytology with genomic and transcriptomic data reveals biological characteristics of wintersweet, such as floral transition in spring, floral organ specification, low temperature-mediated floral bud break, early blooming in winter, and strong cold tolerance.

**Conclusions:**

These findings provide insights into the evolutionary history of wintersweet and the relationships among the Magnoliids, monocots, and eudicots; the molecular basis underlying floral scent biosynthesis; and winter flowering, and highlight the utility of multi-omics data in deciphering important ornamental traits in wintersweet.

## Background

Calycantaceae is a small, evolutionarily ancient family composed of ten species assigned to three genera: *Calycanthus* L, *Chimonanthus*, and *Idiospermum Black* that display differences in flower color, flowering time, and geographical distribution [1, 2]. *Chimonanthus praecox* (Chinese name “La Mei,” commonly known as wintersweet, 2*n* = 22), is a perennial deciduous shrub that belongs to the Calycantaceae family [3]. It originates in China and has been cultivated for over a thousand years. Because of its distinctive fragrant aroma and unique flowering time in winter, wintersweet is also widely cultivated as an ornamental plant in Japan, Europe, and America, with high ornamental and economic value. Additionally, wintersweet is also utilized for its medicinal chemistries, primarily for the treatment of coughs, rheumatism, and measles [4].

Wintersweet flowers possess an intense fragrance that is endowed by a combination of volatile terpenoids (monoterpenes and sesquiterpenes) and benzenoids [5] which are emitted from nectaries distributed on the adaxial of inner petals [6]. These essential oils derived from its flower are widely used as components in perfume, cosmetics, and various flavor industries [7, 8]. Apart from broad industrial applications, the floral scent also functions in attracting and guiding pollinators to ensure its reproductive success, in addition to protecting the vulnerable reproductive organs from florivores and pathogens [9]. Despite the high ornamental and ecological value of floral scent in wintersweet, the molecular mechanisms underlying the biosynthesis and regulation of the floral volatiles are not well understood. Furthermore, genetic resources are limited for wintersweet, which impedes progress on understanding floral scent biosynthesis and metabolism in wintersweet [10].

**Fig. 1:**
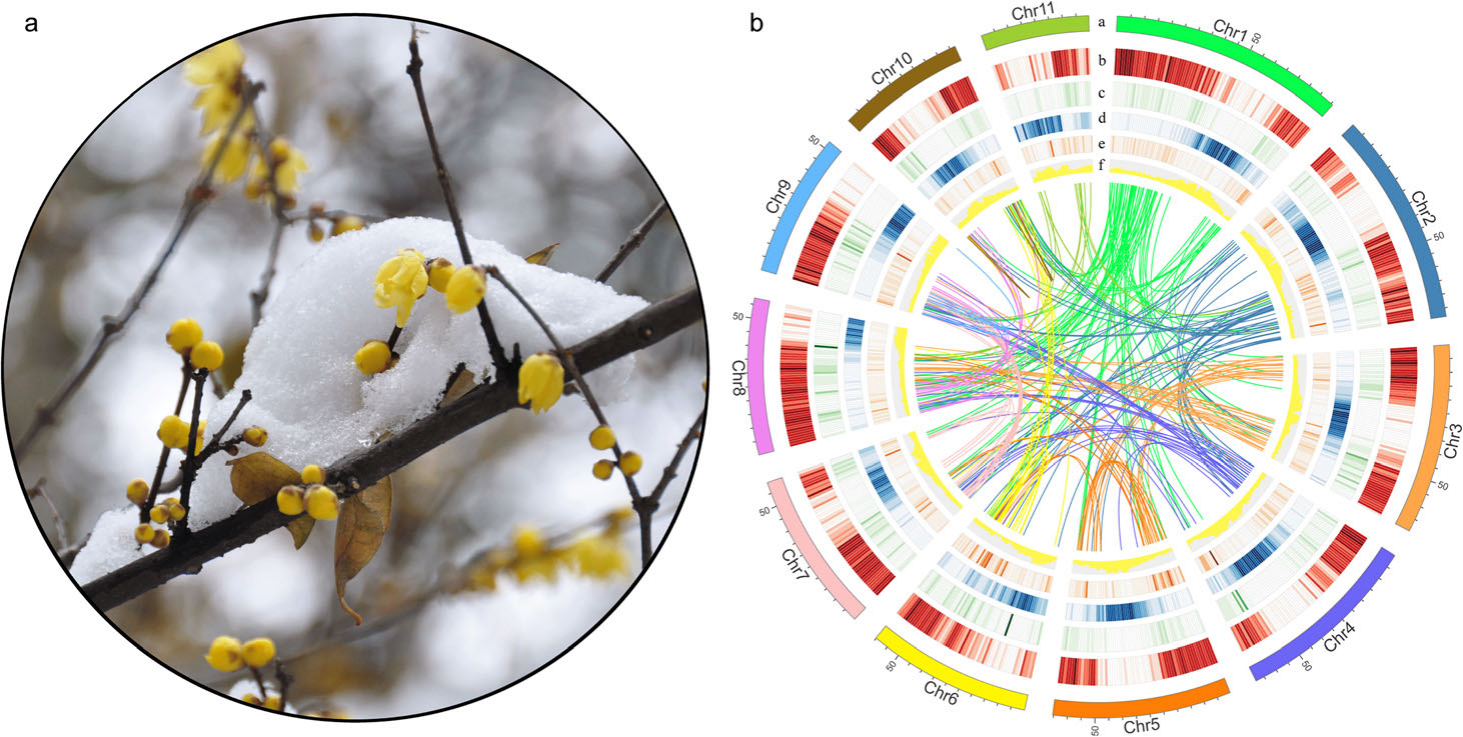
Wintersweet flower morphology, genome features, and synteny information. **a** Wintersweet flower blooming in winter. **b** Overview of the wintersweet draft genome assembly. The outer layer of colored blocks represents the 11 pseudomolecules, with tick marks every 5 Mb in size (i). Tracks displayed are as follows: (ii–v) the density of genes, long interspersed nuclear elements, long terminal repeat retrotransposons and DNA elements; (vi) guanine-cytosine (GC) content; (vii) relationship between syntenic blocks, as indicated by lines. All the colored bands were featured in 1-Mb intervals across the chromosomes

Flowering is a critical developmental milestone in the plant life cycle influenced by endogenous cues and diverse environmental factors [11]. Different trees display diverse seasonal patterns in flowering. Wintersweet has evolved to synchronize flowering with seasonal climate changes (especially temperature fluctuations). Flower initiation occurs in spring, but flowers bloom during winter (typically late December or early January). During summer and autumn, flower buds grow extremely slow completing floral organ specification, differentiation, and maturation. Like most long-lived trees, wintersweet undergoes growth cessation and dormancy establishment prior to the advent of winter. Different from most flowering trees, wintersweet floral buds break and flowers bloom in midwinter (Fig. 1a). This unusual flowering season requires flowers to have strong cold hardiness. Furthermore, wintersweet flowers possess an entirely petaloid perianth without differentiation of sepals and petals [2]. Together, these interesting features offer a unique system for elucidating flowering-time regulation, flower development, floral bud dormancy, and break.

Calycanthaceae belongs to Laurales and, together with Magnoliales, Canellales, and Piperales, constitutes the magnoliids (Magnoliidae). The magnoliids are the third major clades of Mesangiospermae with approximately 9000 species, many of which are early diverging lineages and possess an important phylogenetic position for better understanding the evolutionary history of extant flowering plants [12]. Decades of work have been dedicated to resolving the evolutionary relationships among Magnoliids, monocots, and eudicots; however, the phylogenetic position of Magnoliids relative to monocots and eudicots still remains to be debated. For instance, nuclear genomes of four magnoliids (that is, *Cinnamomum kanehirae*, *Liriodendron chinense*, *Persea americana*, and *Piper nigrum*) have been subsequently published [13–16]; however, phylogenetic analyses of these four genomes resulted in two incongruent placements of Magnoliids relative to monocots and eudicots—that is, either monocots as sister group to a clade consisting of magnoliids and eudicots, or magnoliids as the sister to monocots-eudicots clade [13–16]. Furthermore, the genome evolution within Magnoliidae is also a widely studied topic [13, 14, 17], but still not fully resolved.

In this study, a chromosome-level genome assembly of wintersweet was obtained using a combination data produced from three advanced technologies. Comparative analyses of the wintersweet genome with those of the other four magnoliids and 12 angiosperms have enabled the resolution of the phylogenetic position of magnoliids and yielded new insights into the genome evolution of magnoliids. Through gene mining, cytology, transcriptome, and metabolic data generated from diverse floral developmental stages, we present new insights into the molecular basis of floral scent biosynthesis and flowering in winter.

## Results

### Genome sequencing, assembly, and annotation

The DNA for genome sequencing of wintersweet was obtained from an accession planted in the campus of Huazhong Agricultural University. DNA was extracted and sequenced by combining three different sequencing methods that include Illumina HiSeq, 10X Genomics, and PacBio SMRT sequencing. A total of 76.96 Gb of PacBio long reads were achieved (Additional file 1: Table S1), approximately 98.83-fold high-quality sequence coverage of the 778.71 Mb genome (size estimated by k-mer frequency analysis) (Additional file 2: Fig. S1a and Additional file 1: Table S2). Flow cytometry determined an estimated haploid genome size of 805.88 Mb (Additional file 2: Fig. S1b), which was consistent with the k-mer method. After interactive error correction, the PacBio reads were assembled into primary contigs using FALCON [18]. The primary generated contigs were then polished with Quiver, yielding 1623 contigs with an N50 length of 2.19 Mb (Table 1). The sequence error correction of the final contigs were performed using 36.48 Gb (46.85X) Illumina short reads by pilon [19]. The consensus sequences were further scaffolded by integrating with 156.26 Gb (200.67X) 10X Genomics linked reads (Additional file 1: Table S1). The final assembly consists of 1259 scaffolds totalling 695.31 Mb with a scaffold N50 size of 4.49 Mb, covering 89.2% of the genome size estimated by genome survey (Table 1 and Additional file 1: Table S3). In order to improve the assembly, we used 93 × Hi-C data to assist the assembly correction and anchored 1027 of 1259 scaffolds into 763 super-scaffolds (Additional file 1: Table S4 and S5). All the super-scaffolds were accurately clustered and ordered into 11 pseudochromosomes (Additional file 2: Fig. S2), covering 99.42% of the original 695.31 Mb assembly, with a super-scaffold N50 of 65.35 M and a maximum scaffold length of 85.71 Mb (Additional file 1: Table S5). The number of groups corresponded well with the experimentally determined number of chromosomes in somatic cells (2*n* = 22) (Additional file 2: Fig. S3). In addition, 185.93 Gb Illumina sequence data was also generated and used to assemble the *Calycanthus chinensis* (a close relative of wintersweet belonging to the same family) genome (Additional file 1: Table S1). The size of the assembled *C. chinensis* draft genome was 767.4 Mb, representing ~ 92.78% of estimated genome size (Additional file 1: Table S2), with 291,991 contigs (N50 = 38.7 kb) and 241,923 scaffolds (N50 = 20.34 Mb) respectively (Additional file 1: Table S3).

**Table 1:** Major indicators of the wintersweet genome

Assembly feature	Statistic
Estimated genome size (by k-mer analysis) (Mb)	778.71
Contig N50 (Mb)	2.19
Scaffold N50 (Mb)	65.35
Longest scaffold (Mb)	85.71
Assembled genome size (Mb)	695.36
Assembly % of genome	99.42
Repeat region % of assembly	47.53
Predicted gene models	23,591
Average coding sequence length (bp)	1250
Average exons per gene	5.69

To assess the genome assembly quality, we performed BUSCO and CEGMA analysis and found that 95% and 92.74% complete eukaryotic conserved genes were identified in wintersweet genome respectively (Additional file 1: Table S6), suggesting a high degree of completeness of the final assembly. In addition, the high-quality short reads generated from Illumina were mapped to the assembled genome, which exhibits excellent alignments with a mapping rate of 99.95%. Taken together, the above results indicate a high degree of contiguity and completeness of the wintersweet genome.

Based on de novo and homology-based predictions and transcriptome data, a total of 23,591 protein-coding genes were predicted with an average length of 9017 bp and an average CDS length of 1250 bp, which were comparable to that in *Amborella* and *Lotus* (Additional file 1: Table S7). The spatial distribution of these protein-coding genes along the chromosome was uneven with higher densities located at the ends of the chromosomal arms (Fig. 1b). A total of 21,940 (93.1%) predicted protein-coding possessed functional annotation (Additional file 1: Table S8). A total of 2749 non-coding RNAs (ncRNAs) including 245 ribosomal RNAs (rRNAs), 567 transfer RNAs (tRNAs), 909 microRNA, and 1028 small nuclear RNAs (snRNAs) (Additional file 1: Table S9) were also identified.

### Comparative evolutionary analyses of wintersweet and other typical flowering plant species

**Fig. 2:**
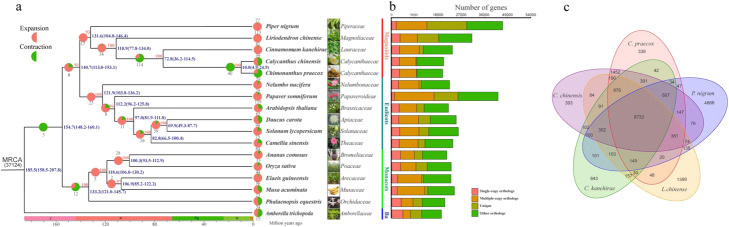
Evolution of the wintersweet genome and gene families. **a** Phylogenetic tree of 17 plant species. The blue numbers denote divergence time of each node (MYA, million years ago), and those in brackets are 95% confidence intervals for the time of divergence between different clades. The red numbers on the branch represent bootstrap value. The pie diagram on each branch of the tree represents the proportion of gene families undergoing gain (red) or loss (green) events. The numbers below the pie diagram denote the total number of expansion and contraction gene families. Basal angiosperm (Ba). **b** The distribution of single-copy, multiple-copy, unique, and other orthologs in the 17 plant species. **c** Venn diagram represents the shared and unique gene families among five closely related magnoliids (*C. praecox*, *C. kanehirae*, *L. chinense*, *P. nigrum*, and *C. chinensis*). Each number represents the number of gene families

The expansion or contraction of gene families has a profound role in driving phenotypic diversity and adaptive evolution in flowering plants [20]. In comparison with gene families in its relative species *C. chinensis*, wintersweet exhibited significant enrichment and reduction of 12 and 45 gene families respectively (Fig. 2a). KEGG functional enrichment analysis of the expanded gene families demonstrates that they were mainly assigned in “Sesquiterpenoid and triterpenoid biosynthesis,” “Monoterpenoid biosynthesis,” “Flavonoid biosynthesis,” and “Phenylpropanoid biosynthesis” pathways (Additional file 2: Fig. S4a and Table S10), which are responsible for the major trait (strong fragrance) specific to wintersweet.

Defining the relationship of gene families among flowering plant species has been a powerful approach in investigating the genetic basis of plant evolution. Based on pairwise sequence similarities, we applied the predicted proteomes of wintersweet and 16 other sequenced species to identify putative orthologous gene clusters. A total of 37,137 orthologous gene families composed of 554,042 genes were identified from 17 plant species, of which 5339 clusters of genes were shared by all investigated species, representing ancestral gene families (Fig. 2b). On the other hand, 8733 gene families were present across wintersweet, *C. chinensis*, *L*. *chinensis*, and *C*. *kanehirae*, which most likely represent the “core” proteome of the magnoliids (Fig. 2c). There are 339 gene families containing 507 proteins specific to the wintersweet genome (Additional file 1: Table S11). Gene Ontology (GO) term enrichment analyses of wintersweet-specific genes revealed that the functional categories termed “oxidoreductase activity” and “pectinesterase activity” involved in metabolism were enriched (Additional file 2: Fig. S4b).

### Repetitive content and recent burst of LTR retrotransposons

In the wintersweet genome, repetitive elements occupied 45.73% of the genome, of which 96.69% were annotated as transposable elements (TEs) (Additional file 1: Table S12). Long terminal repeat retrotransposons (LTRs) were the major class of TEs that accounts for 36.2% of the assembly. Among the LTRs, the LTR/Gypsy elements were the most abundant, composing 23.3% of the genome, followed by LTR/Copia elements (8.6%, Additional file 1: Table S12). Besides the main groups of LTR elements, 3.65% of the genome was annotated as DNA elements and 3.45% as long interspersed nuclear elements, whereas the rest were assigned to other repeat families or could not be assigned (Additional file 1: Table S12). Transposable elements are unevenly distributed across the chromosomes and found to be particularly abundant in centromeric regions (Fig. 1b). Further comparative analysis of the distribution of TEs indicated a higher proportion in intergenic regions (79.19%) when compared to genic regions (16.04%) and regions adjoining genes (4.77%) (Additional file 2: Fig. S5a). Within genic regions, the TEs exhibited unequal distribution between exons and introns. 98.98% of TEs in the genic regions occurred in introns and constituted 25% of the total length of introns (Additional file 2: Fig. S5b). Comparison of gene structure with other species revealed that the average length and number of exons is similar, while the average length of introns is slightly longer and to some extent can be attributed to repeat accumulation. Moreover, the time of the LTR-RT burst in wintersweet was estimated using the 8812 putative complete LTR-RTs and revealed a peak substitution rate at around 0.03 (Additional file 2: Fig. S6). We assumed a mutation rate of 1.51 × 10^− 9^ per base per year [14], resulting in an insertion time of approximately 9.9 Ma.

In order to investigate the evolution of TEs in Magnoliids, phylogenetic trees of domains in reverse transcriptase genes were constructed for both *Ty1/Copia* and *Ty3/Gypsy* superfamily. In the phylogenetic tree of *Ty3/Gypsy* superfamily, the majority of LTR-RTs from wintersweet were clustered into the tork clade (Additional file 2: Fig. S7a). Compared with *L. chinensis* and *C kanehirae*, the LTR-RTs in wintersweet and *C*. *chinensis* exhibited higher diversity and abundance within the tork clade, indicating greater expansion and divergence in wintersweet and *C*. *chinensis* genome. The *Copia* superfamily displayed a different pattern, with four major clades consisting of elements from all these four species (Additional file 2: Fig. S7b), suggesting a conserved evolution pattern of the *Copia* superfamily, as described previously [21, 22].

### Phylogenomic placement of Magnoliids sister to eudicots

The phylogenetic relationships of Magnoliids, monocots, and eudicots have been somewhat controversial in plant taxonomy. In an effort to infer the phylogenetic position of the Magnoliids relative to monocots and eudicots, a set of 213 evaluated single-copy ortholog sets (OSCG) were first identified with OrthoMCL [23] using genome data from 17 flowering plant species that includes 5 monocots, 6 eudicots, 5 magnoliids, and 1 basal angiosperm. We applied both coalescent and concatenation approaches to reconstruct phylogenetic trees using the 213-gene dataset. Both coalescent and concatenation analyses yielded an identical highly supported topology with magnoliids as a sister group to eudicots after their divergence from monocots (Fig. 2a and Additional file 2: Fig. S8a). To avoid the potential errors in ortholog identification, we also used SonicParanoid [24] to extract single-copy genes (SSCG) from the 17 plant genomes described above. Only those genes sampled from at least 14 species were utilized for the construction of phylogenetic trees. On the basis of 216 single-copy genes, the phylogenetic trees were then similarly inferred by both coalescent and concatenation methods as those described above. The resulting species trees were topologically identical to the phylogenetic findings revealed by OrtholMCL described above (Additional file 2: Fig. S8b).

Although the same set of phylogenetic relationships among Magnoliids, monocots, and eudicots was consistently recovered, the topological conflicts were also observed among coalescent-based gene trees (Additional file 2: Fig. S8c). To estimate the discordance among gene trees in OSCG and SSCG datasets, we took advantage of the quartet score in ASTRAL [25] to display the proportions of gene trees in support of three different branching orders for Magnoliids, monocots, and eudicots (Additional file 2: Fig. S8d) and found that the percentages of gene trees supporting Magnoliids and eudicots together forming a sister group with monocots is higher than the other two topologies (Additional file 2: Fig. S8c). However, in the phylogenetic analyses of a concatenated sequence alignment of 38 chloroplast single-copy genes for 26 taxa, the magnoliids were placed as a sister group to the clade consisting of eudicots and monocots (Additional file 2: Fig. S9). Furthermore, the short phylogenetic branches among magnoliids, eudicots, and monocots clades, representing rapid speciation events, were also observed in these phylogenetic genes. The phylogenetic incongruence between nuclear and plastid genomes may be caused by incomplete lineage sorting (ILS), which appears more frequently during the rapid divergence of early mesangiosperms. As the inadequate taxon sampling could result in incongruent phylogeny, we improved the taxon sampling by adding additional genome data from 11 phylogenetically pivotal species and a transcriptome data set of chloranthales to reconstruct the phylogenetic tree. This approach recovered the same phylogenetic relationships among magnoliids, eudicots, and monocots (Additional file 2: Fig. S10). Thus, from these results, we believe the phylogenetic relationship proposed in our study is relatively accurate under the current dataset. Based on the high-confidence phylogenetic tree and calibration points selected from articles and TimeTree website, the divergence time between the magnoliids and the eudicots were estimated to be 113.0–153.1 Ma (95% confidence interval) (Fig. 2a), which overlaps with three recent estimates (114.6–164 Ma, 117–189 Ma, and 136.0–209.4 Ma) [13, 25, 26].

### Whole-genome duplication and genome evolution analysis

**Fig. 3:**
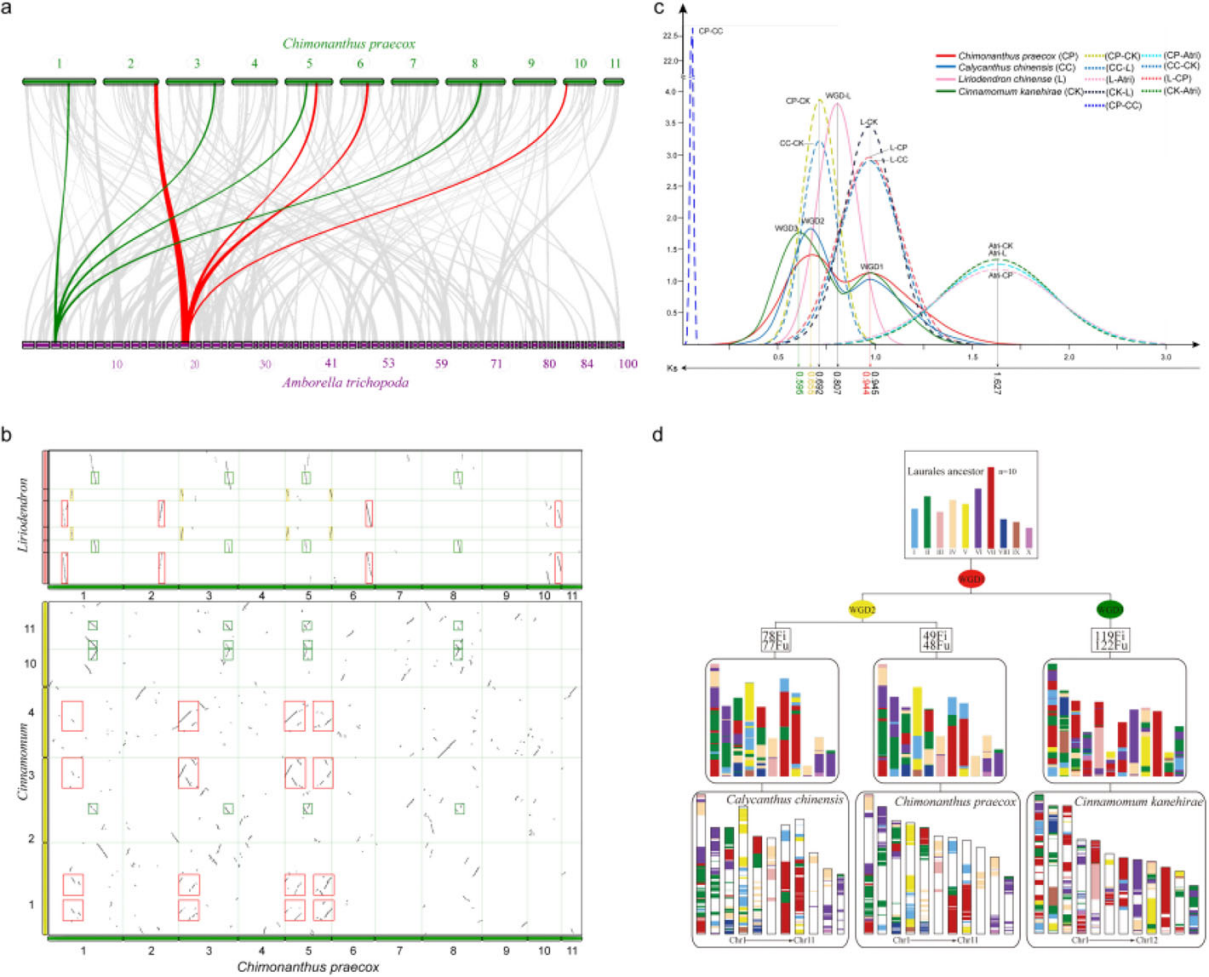
Comparative genomics analyses. **a** Synteny patterns between genomic regions from wintersweet and Amborella. This pattern shows that some typical ancestral regions in the basal angiosperm Amborella have four corresponding copy regions in wintersweet. This collinear relationship is highlighted by one syntenic set shown in red and green colors. **b** Syntenic blocks between genomes. Dot plots of orthologs show a 4–4 chromosomal relationship between the wintersweet genome and *C. kanehirae* genome, and 2–4 chromosomal relationship between wintersweet genome and *L. chinense* genome. **c** Distribution of synonymous substitution levels (Ks) of syntenic orthologous (dashed curves) and paralogous genes (solid curves) after evolutionary rate correction. **d** Evolutionary model of the Laurales genomes. The Laurales ancestral chromosomes are represented by ten colors. Polyploidization events are shown by 3 dots of different colors, along with the chromosome fusions (Fu) and fissions (Fi). The modern structure of the Laurales genomes is illustrated at the bottom of the figure. In some regions, we could not determine which ancestral chromosome they derived from, and those regions were represented as white spaces

Whole-genome duplication (WGD) has long been regarded as the major driving force in plant evolution [27]. To investigate WGD events during the evolutionary course of wintersweet, we first searched for genome-wide duplications and assigned them into four different modes with MCScanX analysis (Additional file 2: Fig. S11). The WGD/segmental duplication was identified as the dominant type that includes 4511 paralogous gene pairs in 265 syntenic blocks. Among these syntenic blocks, 36.09% were found to share relationships with three other blocks across the genome (Additional file 2: Fig. S12). The widespread synteny and well-maintained one-versus-three syntenic blocks suggest that two WGD events might have occurred during wintersweet genome evolution. It is well accepted that *Amborella* is a single living species that is the sister lineage to all other groups within the angiosperms, and there is no evidence of lineage-specific polyploidy after it diverged from the last common ancestor of angiosperms [28]. Collinearity and synteny analysis between the wintersweet and *Amborella* genome also provided clear structural evidence for two WGDs in wintersweet with a 1:4 syntenic depth ratio in *Amborella*-wintersweet comparison (Fig. 3a). To further elucidate the polyploidy of wintersweet genomes, we performed a comparative genomic analysis of wintersweet with *C. kanehirae* and *L. chinensis*. Syntenic depth ratios of 4:4 and 2:4 were inferred in the wintersweet-*Cinnamomum* and wintersweet-*Liriodendron* comparisons, respectively (Fig. 3b). Based on the syntenic relationships between and within each species, our analyses collectively indicate that wintersweet underwent two WGD events.

To estimate the timing of the two WGD events in the wintersweet genome, we characterized synonymous substitutions on synonymous nucleotide sites (Ks) between collinear homoeologs within or between wintersweet and other three species including *C. chinensis*, *Cinnamomum kanehirae*, and *Liriodendron chinensis* from Magnoliids. The Ks distributions of one-to-one orthologs identified between *Amborella* and the other four species show different Ks peaks, suggesting divergent evolutionary rates among these four species (Additional file 2: Fig. S13). After correction for evolutionary rate [29], the synonymous substitutions per site per year as 4.21 × 10^− 9^ for Laurales were calculated using the mean Ks values of syntenic blocks, resulting in the estimated time of the WGD event at approximately 77.8 million and 112.1 million years ago (Ma), respectively (Fig. 3c). Previous analysis of the genome of *Cinnamomum* suggested that the ancient WGD event seems shared by Magnoliales and Laurales [13], and the absolute dating of the identified WGD events in *Liriodendron tulipifera* also supported this hypothesis [14]. In our study, we also detected two and one polyploidization events in *Cinnamomum* and *Liriodendron* respectively, but no common WGD event was shared by these two species. Furthermore, the wintersweet genome shares an ancient WGD event with *Cinnamomum* but not with *Liriodendron*. Moreover, the trees of the syntenic gene groups of wintersweet and *Liriodendron* vs. *Amborella* indicated that wintersweet and *Liriodendron* experienced a WGD event respectively after their divergence from a common ancestor (Additional file 2: Fig. S14 and Additional file 3: Supplementary Note 3). Thus, from these results, we conclude that the ancient wintersweet WGD event has occurred before the divergence of Calycantaceae and Lauraceae but after the divergence of Calycantaceae and Magnoliaceae.

We also used orthologous and paralogous genes derived from the intergenomic and intragenomic analysis of the wintersweet and *C. chinensis* as well as *C*. *kanehirae* genomes to construct a putative ancestral genome of the Laurales, and proposed an evolutionary scenario where these three lineages were derived from a putative ancestor (Fig. 3d and Additional file 2: Fig. S15), which consisted of ten chromosomes and 4216 genes. This ancestor went through a WGD event to reach a 20-chromosome intermediate and then experienced chromosomal rearrangements to form present-day karyotypes. In wintersweet, all the chromosomes underwent rearrangements and every chromosome came from at least two ancient chromosomes. A minimum of 49 chromosomal fissions and 48 chromosomal fusions were predicted to have occurred in wintersweet to reach its current structure of 11 chromosomes (Fig. 3d).

### Genetic basis of floral transition, floral organ specification, and early blooming in winter

**Fig. 4:**
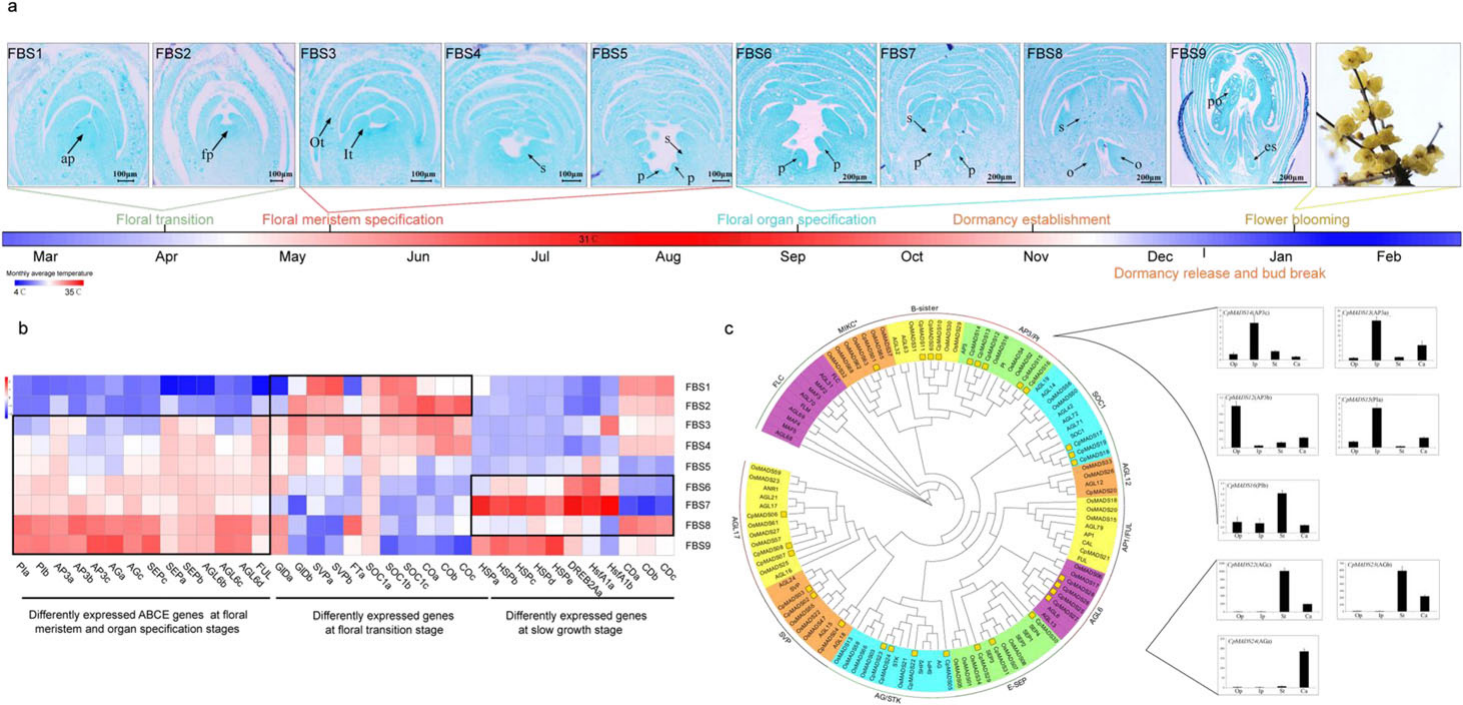
Schematic depiction of the key developmental stages of flower bud and the analysis of floral organ identity and flowering-time related genes. **a** Flower developmental stages including floral transition, meristem specification, floral organ specification, floral bud dormancy and release, and blooming. Abbreviations for the flower bud developmental stages: undifferentiated flower bud stage (FBS1); flower primordium formation stage (FBS2); tepal primordium formation stage (FBS3); stamen primordium formation stage (FBS4); pistil primordium formation stage (FBS5); flower organ development and differentiation stage (FBS6); slow growth stage (FBS7); ovule appearance stage (FBS8); pollen formation stage (FBS9), blooming in winter. ap, shoot apex; fp, floral primordium; t, tepals; s, stamens; p, pistil; a, anther; o, ovule; po, pollen; es, embryonic sac. **b** The expression patterns of genes related to flowering time and floral organ identity at different flower developmental stages. **c** The phylogenetic tree of MADS-box genes and gene expression patterns of B-/C-function genes from various floral organs. The MADS-box proteins in wintersweet are marked by the yellow box

Wintersweet is one of the perennial trees that bloom in the deep winter. It took approximately 10 months for *C. praecox* to complete its reproductive development. To investigate this whole process of the flower development that may influence the final flowering time, we first performed a systematic study on the floral ontogeny and developmental patterns by paraffin sections through observation. The results indicated that the floral bud was initiated in April, floral patterning and floral organ specification occurred from April to July, slow growth in summer, the male and female gametophytes were formed in October and December respectively, the flower bud transitioned into dormancy, then break occurred in December, and the flower bloomed in deep winter (Fig. 4a). To investigate the molecular mechanisms underlying the critical flower developmental stages, we generated and analyzed RNA-seq data for representative flower developmental stages from the timing of floral initiation to maturation.

Floral initiation is controlled by the spatial and temporal expression of flowering-time-related genes in multiple pathways [11]. Many genes from these pathways have been identified and characterized in various herbaceous and perennial species and reported to have the conserved function [30–32]. A database of flowering-time gene networks was recently constructed in *Arabidopsis thaliana* [30]. Taking advantage of this database, we identified 594 flowering-time genes in eight pathways (Additional file 1: Table S13). Analysis of RNA-seq data shown that during the floral transition the flowering-time genes related to gibberellin biosynthesis and signaling transduction pathway were significantly activated (padj < 0.01), and the expression of some genes in photoperiodic and circadian clock pathways were also upregulated (Fig. 4b and Additional file 1: Table S14) suggesting the endogenous hormone (gibberellin) and environmental factor (photoperiod) may play a major role in the switch from vegetative to reproductive growth in spring.

After floral patterning and floral organ specification from April to July, the floral organ development processes slowly from Summer to Autumn, during which the temperature is very high and maximum temperature could reach up to 39 °C (Fig. 4a and Additional file 1: Table S15). Therefore, the temperature may be a key factor that affects the flower organ slow development. The direct reflection is the significantly increased expression level of heat shock protein genes (Fig. 4b). In addition, comparing with other developmental stages, many genes associated with cell division downregulated significantly (Fig. 4b and Additional file 1: Table S16). Heat stress transcriptional factors (HSFs) and heat responsive genes play an essential role in heat stress response [33]. We have found 21 members in HSF family in *C. praecox*. Six *HsfA1s*, which serve as the master transcriptional regulators in the heat stress response, were identified (Fig. 4b and Additional file 1: Table S17). The DEHYDRATION-RESPONSIVE ELEMENT BINDING PROTEIN2A (DREB2A) regulated by the HsfA1s. Both *HsfA1-1* and *DREB2A-1* displayed an opposite expression pattern with the genes related to cell division (Fig. 4b and Additional file 1: Table S15). The DRE sequence (CTAGA motif) which is recognized by DREB2A was also detected in the promoter of the cell division genes (Additional file 2: Fig. S16a). These results may suggest that the heat signals can be integrated into transcriptional regulatory networks by the HSFs then to regulate expression of the genes related to the cell division, finally resulting in the slow growth of flower organs.

In total, 58 MADS-box genes were identified in the wintersweet genome, 31 of which are MIKC-type MADS-box genes (Additional file 1: Table S13). Phylogenetic and collinearity analyses of these genes indicated that the homologs of ABCE model prototype genes, except for *AP1/FUL*, were all found to be duplicated (Fig. 4c). Four *AGL6s*, generated by WGD events, were identified in wintersweet assembly, the number of which is larger than that in Arabidopsis and rice. Among these genes, the *CpAGL6a* has been reported to promote flowering when overexpressed in *Arabidopsis* [34]. Meanwhile, the FLOWERING LOCUS C (*FLC*), which serves as a flowering repressor [35], was lost in wintersweet (Fig. 4c). The selective expanded promoter and loss of repressor of flowering-time related genes may be associated with the earlier flowering of wintersweet. The morphology between the inner and outer perianth in wintersweet displayed a slight difference, which is the same as in some basal eudicots (*Ranunculus* and *Aquilegia*). “Sliding boundary” model was proposed to explain this morphology in *Ranunculus*, in which the B-function homologs are expressed in those whorls that produce petaloid organ [36]. The B-function homologs in wintersweet displayed broad expression pattern, with *AP3b* preferably expressed in the outer perianth whorl (Fig. 4c), which supported the “sliding boundary” model and may complement the absence of a clear morphological distinction between sepals and petals in wintersweet. The strong expression of C-function homologs is limited to stamen and carpel, which suggests that these genes have a conserved function in stamen and carpel specification. The expression profiles of wintersweet ABCE homologs during the flower development largely agree with the gradual formation of the floral organs they specify (Fig. 4b).

The relative earlier flowing time in winter suggested the shorter chilling requirement for dormancy release and earlier bud break in wintersweet. The members of the *SHORT VEGETATIVE PHASE* (*SVP*) clade of the MADS-box gene family, including SVP and DAM genes, are well known to be associated with dormancy release and bud break [37, 38]. Two homologs of *SVP* genes were identified in the wintersweet genome. Phylogenetic analysis of *SVPs* revealed that these two genes cluster close to *PtSVL* (Additional file 2: Fig. S16b), the function of which had been characterized as a repressor in the genetic network of temperature-mediated vegetative bud break in hybrid aspen [39]. The downstream genes of *PtSVL* in the network including *TEOSINTE BRANCHED1*, *CYCLOIDEA*, *PCF/BRANCHED1* (*TCP18/BRC1*), and *FLOWERING LOCUS T* (*FT*), which function as negative and positive regulators of temperature-mediated control bud break, were also identified in the wintersweet genome (Additional file 2: Fig. S16c). During the transition from endodormancy to flush stage, the increase in expression of *CpFT1* and downregulation of *CpTCP18/BRC1* and *CpSVL1* were noted (Additional file 2: Fig. S16d). Gibberellin acid (GA) and abscisic acid (ABA) acts as positive and negative regulators of bud break respectively [40], and the content of which to some extent is associated with the expression level of biosynthesis and catabolism genes. The increased expression of GA biosynthesis genes such as *GA20 oxidase* and the decreased expression of ABA biosynthesis genes such as the *NCED* (Additional file 2: Fig. S16d) was also observed at the bud break stage, which may coincide with their role in bud break.

### Genetic basis of strong cold resistance

Wintersweet is one of the perennial trees that bloom in the deep winter, during which the temperature always falls below the freezing point. Therefore, wintersweet has evolved a systematic mechanism to withstand cold stress. Volatile glycosylation is a common form of plant volatile compounds and plays an important role in response to abiotic stress in plants [41]. Recent studies revealed that the volatile terpene glucosylation mediated by UDP-glycosyltransferases (UGTs) was involved in the modulation of cold stress tolerance in tea plants [42]. In wintersweet, abundant volatiles were present in glycosidically bound forms, such as linalool glucoside, benzaldehyde benzyl alcohol (Additional file 2: Fig. S17). The considerable expansion in the UGT family (Additional file 1: Table S10) and abundant terpene glycosides in wintersweet flowers lead to the hypothesis that the strong cold tolerance of wintersweet, to some extent, is related to the volatile glucosylation.

### Evolution of terpene biosynthesis and regulation-related genes

**Fig. 5:**
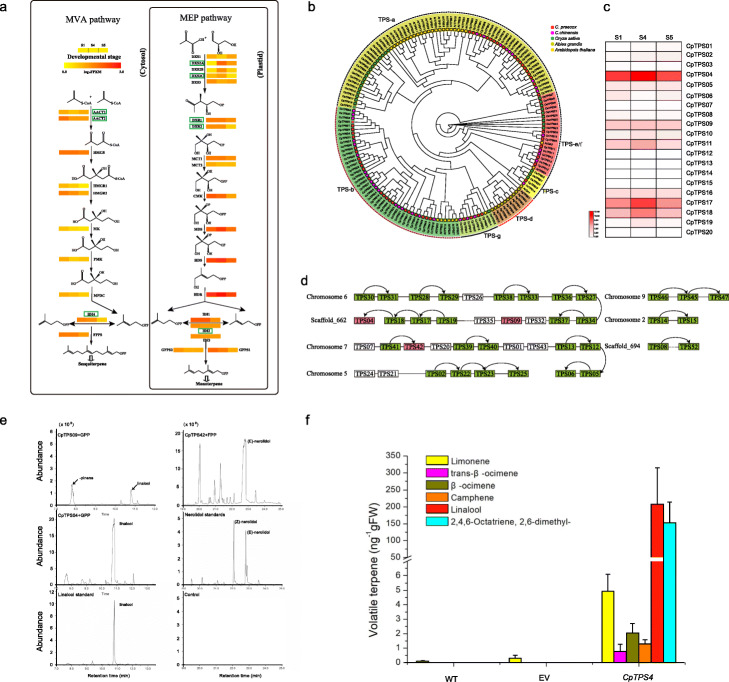
Terpenes biosynthesis in wintersweet. **a** Expression profiles of genes encoding enzymes possibly involved in monoterpene and sesquiterpene biosynthesis. Abbreviations for enzymes in each catalytic step are shown in bold. The gradient color for each gene represents the gene expression levels in three floral developmental stages (S1: bud stage; S4: full open flower stage; S5: senescence stage). Homologous genes are represented by equal colored horizontal stripes and are termed from top to bottom in Arabic numerical order. The full names of enzymes are listed in the Additional file 1: Table S18. The genes circled in green box were generated by WGD event. The non-boxed genes did not undergo this event. **b** Phylogeny of TPS proteins identified in wintersweet and 5 other sequenced plant genomes showing the subfamilies from a–g. **c** The expression of *CpTPS* genes in three different floral development stages. These genes were expressed in at least one of the three developmental stages. **d** The chromosomes with more than one *CpTPS* gene. The red diamonds represent the functionally characterized genes. The green diamonds represent the genes generated by tandem duplication events. The cure with arrow linked two duplicated genes. **e** Identification of enzymatic products after incubating recombinant CpTPSs proteins with geranyl diphosphate (GPP)/farnesyl diphosphate (FPP). The volatile terpenes were analyzed by GC-MS analysis and comparing with authentic standards. **f** Increased linalool biosynthesis in the *CpTPS4*-overexpressed *tobacco* compared with wild type (WT) and empty vector control (EV). Data represent the mean ± SDs of three biological replicates

Monoterpenes are the major components of floral volatile organic compounds (VOCs) in wintersweet, especially the linalool, which accounts for more than half of the floral scent [5]. In plants, monoterpenes/diterpenes and sesquiterpenes are usually generated via the 2-*C*-methyl-D-erythritol 4-phosphate (MEP) pathway and the mevalonate (MVA) pathway, respectively [43]. A total of 46 genes in these two pathways were identified (Additional file 1: Table S18). The key genes involved in the MEP pathway such as 1-deoxy-d-xylulose 5-phosphate synthase (*DXS*), 1-deoxy-d-xylulose 5-phosphate reductoisomerase (*DXR*), and isopentenyl diphosphate isomerase (*IDI*) were generated through WGD events (Fig. 5a). The high rate of paralog generation in these genes could increase the efficiency of catalytic reaction through dosage effects, thereby increasing the metabolic flux toward the MEP pathway. Terpene synthases (TPSs) are the enzymes responsible for the last catalytic reaction in the MVA and MEP pathway to generate terpenoid compounds. With the aid of the assembly genome, a total of 52 complete *CpTPSs* were identified (Additional file 1: Table S19), the number of which is approximately double that detected by transcriptomics in our previous study [5]. Phylogenetic analysis of TPS from four species revealed that *CpTPSs* were clustered into five of six subfamilies described for land plants (Fig. 5b). The majority of *CpTPSs* were placed in the TPS-a (18) and TPS-b (24) subfamilies, which is predominantly composed of angiosperm-specific sesquiterpene and monoterpene synthases respectively [44]. Comparative genomics analysis revealed that the TPS genes are significantly expanded, especially in the TPS-b subfamily (Fig. 5b and Additional file 1: Table S10). These lineage-specific gene expansions in the TPS-b subfamily may contribute to the monoterpene accumulation in floral VOCs in wintersweet.

Expression analysis of the 52 *CpTPS* genes by RNA-seq revealed that six genes displayed similar expression patterns with the emission of major monoterpenes (Fig. 5c and Additional file 2: Fig. S18). Based on the expression pattern and phylogenetic analysis, we further selected three genes from TPS-b/g subfamily for functional characterization and found that all the genes encoded versatile enzymes with multiple products (Fig. 5e). Subcellular localization analysis showed that *CpTPS4* and *CpTPS9* were localized to the plastid whereas *CpTPS42* was targeted to the cytosol (Additional file 2: Fig. S19). *CpTPS42* was shown to be a sesquiterpene synthase, which mainly catalyzed the formation of nerolidol, together with other sesquiterpenes (Fig. 5e). *CpTPS4* and *CpTPS9* are both monoterpene synthases and produce β-pinene and linalool as its main product respectively (Fig. 5e). To further understand the function of *CpTPS4*, we also overexpressed the gene in tobacco. Enhanced levels of the monoterpenes including linalool, limonene, β-ocimene, and trans-β-ocimene were found in transgenic tobacco leaves in comparison with the wild type control (Fig. 5f). These results indicated that *CpTPS4* plays a primary role in the biosynthesis of linalool, the main components of floral scent.

The available genome assembly allows for *CpTPSs* to be localized to either chromosomes or scaffold positions to consider a genomic context. The *CpTPS* genes are not uniformly distributed throughout the chromosomes with 44 genes located on six chromosomes and eight genes on seven scaffolds (Fig. 5d). Fourteen of the 52 *CpTPS* genes have at least two copies and each duplicated gene copy was located adjacent to the other. For example, *CpTPS4* is located on scaffold662 and has three copies including *CpTPS17*, *CpTPS18*, and *CpTPS19*. These three genes were arranged as a tandem array on chromosome 6 and highly expressed at the full open flower stage, which may have a similar function as *CpTPS4* and contribute equally to linalool production.

Terpenoid formation does not only depend on the biochemical properties of enzymes encoded by *CpTPS* genes but also requires the involvement of transcription factors (TFs). A total of 1313 TFs that show differential expression during petal development have been identified. Among these, 99 display positive correlation with the emission of terpenes and are predominantly distributed in MYB, bHLH, WRKY, and bZIP families (Additional file 2: Fig. S20 and Additional file 1: Table S20). The transcriptional control of terpene biosynthesis genes correlates with the presence of cis-elements in their promoter regions, which were recognized and bound by specific transcription factors. When screening the 2000-bp regions upstream of the 52 *CpTPS* genes, several defense and stress responsive elements were found to be significantly enriched, such as bHLH- and MYB-binding elements (Additional file 2: Fig. S21). The results indicated that the MYB/bHLH transcription factors may serve as key factors in regulating the *CpTPS* genes expression and provide us with the starting point for the further studies to reveal the cross-talk in the regulation of plant secondary metabolites and stress responses.

### Evolution of benzenoid/phenylpropanoid biosynthesis-related genes

**Fig. 6:**
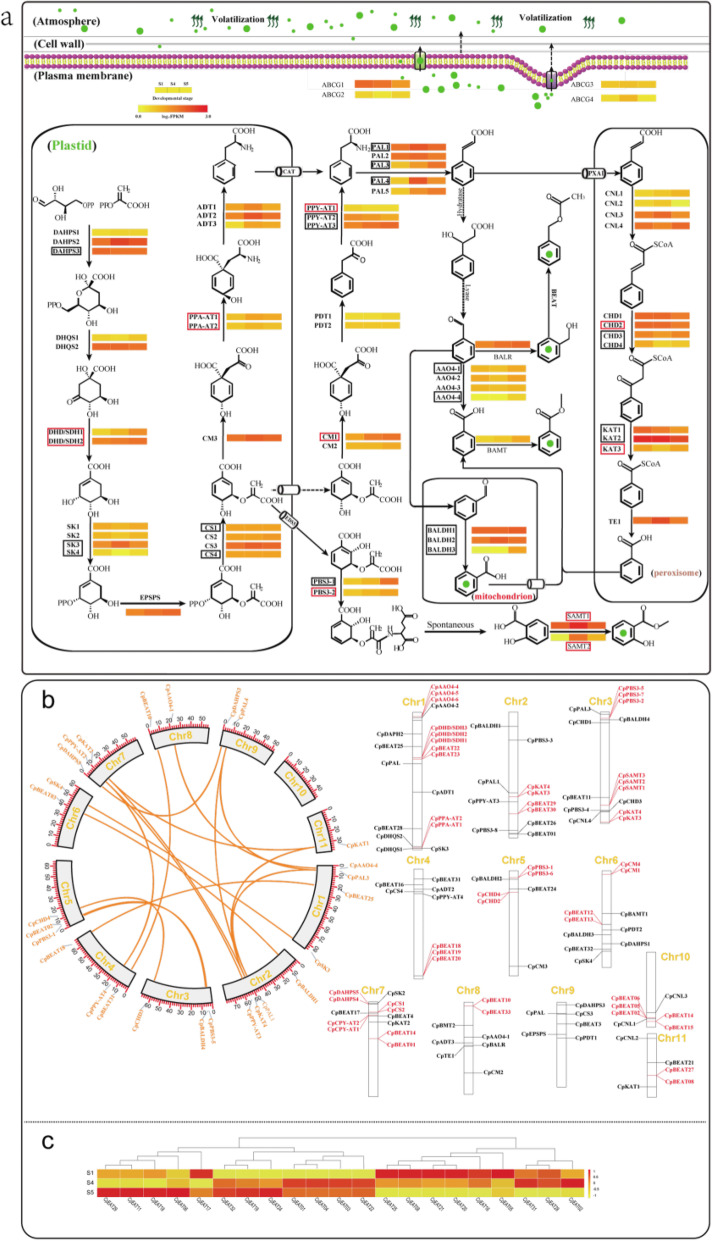
Evolution and expression of key genes involved in benzenoid/phenylpropanoid biosynthesis. **a** Expression profiles of genes encoding enzymes possibly involved in the shikimate/benzenoid pathway in wintersweet. Abbreviations for enzymes in each catalytic step are shown in bold. The gradient color for each gene represents the gene expression levels in three petal developmental stages in wintersweet (S1: bud stage; S4: full open flower stage; S5: senescence stage). Homologous genes are represented by equal colored horizontal stripes and are termed from top to bottom in Arabic numerical order. The full names of enzymes are listed in the Additional file 1: Table S21. The genes circled in black and red boxes were generated by WGD and tandem duplication events respectively. The non-boxed genes did not undergo these events. **b** Schematic representation of the wintersweet chromosomes together with the positions of key genes involved in benzenoid/phenylpropanoid biosynthesis. The genes marked in brown and red were generated by WGD and tandem duplication events respectively. **c** Expression profiles of the 21 *BEAT* homologous genes in three different stages (S1, S4, and S5). These genes were expressed in at least one of the three developmental stages

Benzenoids/phenylpropanoids are the second largest group of the floral VOCs in wintersweet, which are derived from the aromatic acid phenylalanine. Phenylalanine is synthesized via two pathways (phenylalanine pathway and aragenate pathway) [45], and these two pathways split from the plastidial shikimate pathway [46]. The genes involved in the shikimate pathway (20), phenylpyruvate pathway (7), and arogenate pathway (6) were identified as shown in Fig. 6a. In the wintersweet genome, both WGD and tandem duplication events have considerably impacted both the upstream genes in the phenylpropanoid pathway and downstream genes involved in specific benzenoid (benzyl acetate and methyl salicylate) biosynthesis (Fig. 6a,b), which lead to the high rate of paralog formation in 15 gene families (Additional file 1: Table S21).

Benzyl acetate, the dominant compounds of floral scent in wintersweet, is synthesized from benzyl alcohol via acetyl-CoA-dependent reaction catalyzed by acetyl-CoA: benzyl alcohol acetyltransferase (BEAT) [47]. Comparative genomic analysis revealed that the wintersweet genome harbors 33 *BEAT* homologous genes (Additional file 1: Table S21), the number of which is comparable to that in *Prunus mume*, in which benzyl acetate is also the major component of floral scent. Similar to *P. mume*, the expansion of the *BEAT* homologous genes was mainly attributed to tandem and WGD duplication events [47]. Of 33 *BEAT* homologous genes found in wintersweet genome, 8 were derived from the WGD event, and 14 were amplified via tandem duplication. Transcriptome and metabolite correlation analysis showed that the expression pattern of 4 *CpBEATs* coincided with benzyl acetate emission (Fig. 6c and Additional file 2: Fig. S18). These genes might be responsible for benzyl acetate biosynthesis in the wintersweet flower. Methyl salicylate is also the major composition of floral VOCs in wintersweet. Three tandem duplication-derived salicylic acid methyltransferase (*SAMTs*) were identified in the wintersweet (Additional file 2: Fig. S22), two of which were highly expressed in the flower and their expression patterns correlated with methyl salicylate emission, suggesting that these two genes may be primarily responsible for methyl salicylate biosynthesis (Fig. 6a). These observations above suggested that the expansion of specific genes and selective expression in flower could induce the heightened activity of the corresponding enzymes, which resulted in the abundant characteristic aroma formation in the flowers of wintersweet.

## Discussion

In this study, we constructed a high-quality chromosome-level genome assembly for wintersweet by combining the long-read sequences from PacBio with highly accurate short reads from Illumina sequencing and using Hi-C data for super-scaffolding. The assembly of wintersweet adds to the growing body of genome information for the Calycantaceae family. As the relatively domesticated species in the Calycantaceae family [48], wintersweet has a range of specific biological features such as early blooming in deep winter, strong cold resistance, and fragrant flowers [4, 10, 49]. As a representative of the Magnoliids, it also maintains a key evolutionary position on the tree of life. The availability of the wintersweet genome sequence makes it possible to consider deep angiosperm phylogenic questions, determine genome evolution signatures, and to reveal the genetic basis of interesting traits. This assembly also facilitates in-depth fundamental comparative genomic analysis to elucidate biology and gain resolution of genome evolution between wintersweet and other species within the Calycantaceae family.

Resolution of the relationship among Magnoliids, monocots, and eudicots has not been conclusively determined, despite numerous attempts. In four independent studies, four genomes representing three orders (Magnoliales, Piperales, and Laurales) within magnoliales have been published [13–16], and each study attempted to resolve the phylogenetic position of magnoliidaes. Three species including *Piper nigrum* (representative of Piperales clade), *L. tulipifera* (representative of Magnoliales clade), and *P. americana* (representative of laurales clade) were placed as sister to the monocots and eudicots, while *C. kanehirae* (representative of Magnoliales clade) was found to be a sister clade to the eudicots. Many factors could be responsible for these topological differences such as taxon sample size [50], possible incomplete lineage sorting (ILS) [17], and the number of retrieved orthologs [51]. For example, adequate taxon sampling, especially those smaller sister lineages such as Chloranthales in angiosperm clades [52], was vital to obtain a resolved phylogeny. To account for incomplete lineage sorting, we used two complementary tools to extract the single-copy genes and two methods (coalescent and concatenation-based analysis) to reconstruct the phylogeny. In addition, we also improved taxon sampling, selected key lineages (representative of chloranthales clade) as well as additional lineages in the monocots and eudicots, and included five magnoliids to cover key representative clades. Finally, all the analyses recovered the magnoliids together with eudicots as sister to the monocots. This result is congruent with a recent study of 59 low-copy nuclear genes from 26 mesangiosperm transcriptomes [51] and 410 single-copy genes nuclear gene families extracted from genomic and transcriptomic data from 1153 species [53], but disagrees with the plastid trees which supported a topology of magnoliales as the sister to monocots and eudicots. In comparison with nuclear genes, the plastid genes are uniparentally inherited and may recover different deep-level relationships resulting from ancient lineage sorting and hybridization, which might potentially introduce biases and errors to phylogenetic reconstruction [51]. To date, the genome data were still absent in the key clades of angiosperms, such as Chloranthales. Even though we have suggested a robust phylogeny using “genome-scale” data, sequencing of the complete angiosperm lineages will facilitate future investigations of the phylogenetic relationships of flowering plants.

Wintersweet is one of the very few flowering plant lineages that bloom in winter, which make it an ideal perennial plant for flowering-time study. Application of database about the flowering-time gene networks in *Arabidopsis thaliana* serves to identify the homologs of flowering-time genes in wintersweet. Comparative transcriptome analyses provide an array of resources for further flowering-time-related gene identification. Mapping quantitative trait loci (QTL) onto linkage maps, with segregating genetic populations, is a powerful strategy to dissecting complex agronomical characters [54]. The availability of high-quality genome and diverse germplasm of wintersweet with different flowering time makes it possible to use this genetic approach to detect flowering-time quantitative trait loci in the future. The petaloid sepal is another striking distinction of wintersweet. This flower structure also exists in some basal eudicots (such as *Ranunculus* and *Aquilegia*), some monocots (such as *Liliumus* and *Tulipa*), and basal angiosperm lineages, which was supposed to be displayed by the ancestral angiosperm flower [55]. The broad expression pattern of B-function genes was shared by these species, which may represent the ancestral condition for angiosperms. The genetic network for seasonal temperature-mediated control of bud break has been elucidated in the vegetable bud of hybrid aspen [39]. In this genetic network, the *FT* and *SVL* are the homologs of *FT* and *SVP* in Arabidopsis respectively, both of which act as a flowering regulator [56]. Similar to the vegetable buds, the floral buds are also subjected to the dormancy and bud break. The homologs of the key components in wintersweet displayed a similar expression pattern during the transition from endodormancy to bud break stage, leading us to the hypothesis that wintersweet may utilize the common signaling components in both flowering and bud break process.

The evolution, adaptation, and domestication of wintersweet resulted in specific qualities and quantities of floral volatiles, primarily consisting of monoterpenes and benzenoids [7]. The diversification of terpenes is mainly determined by the *TPS* family genes, among which the *TPS*-b subfamily is well known for monoterpenes synthesis [44]. The extensive expansion of *TPS*-b subfamily genes in the wintersweet genome may be one explanation for diverse monoterpene accumulation. The production of terpenes is regulated to a large extent by the transcription level of *TPS* genes [43]. The results of the present expression analyses revealed a dynamic expression of the *TPS* genes, which may be another explanation for the monoterpene diversification. Using the genomic data, we found remarkable duplications of the metabolic genes in both terpene and benzoid/phenylpropanoid biosynthesis pathways, especially in the *TPS* and *BEAT* genes which are responsible for the major components (linalool and benzyl acetate) production. Tandem duplication is the major contributor to the expansions of *TPS* and *BEAT* genes and most of these duplicated genes are tandemly organized in clusters. In the *Drosophila melanogaster* genome, the *Adh* gene is tandemly duplicated and shows a 2.6-fold greater expression than the single-copy gene. The overactivity caused by the tandem arrangement was proposed to be a general property of tandem gene duplicates [57]. The greater output of the tandem arrangement in the *TPS* and *BEAT* genes may increase transcript abundance of the tandem duplicates and thereby led to the mass production of major components. Based on our data, we speculate that the remarkable duplication, tandem clustering of gene, and gene expression dynamics may contribute to the abundant characteristic aroma formation in wintersweet.

## Conclusions

In summary, we have presented a high-quality assembly of wintersweet, the first genome reference in the Calycantaceae family. The integration of multi-omics data advanced our understanding of floral scent biochemistry as well as the molecular mechanisms underlying flowering. Over a thousand years of wintersweet cultivation, evolutionary forces have shaped abundant cultivars with novelty in floral scent and color. The available complete wintersweet genome will provide a fundamental resource for comparative genomics studies on the diversity and the evolutionary mechanisms of ornamental traits (floral scent and color) at the genome level, which will be invaluable for genetic improvement through molecular breeding in the future.

## Materials and methods

### Plant materials, library construction, and genome sequencing

All plant materials used in this study were collected from the campus of Huazhong Agriculture University (Wuhan, China) under natural photoperiod. High-quality, intact genomic DNA was extracted from fresh leaves of *Chimonanthus praecox “H29”* and *Calycanthus chinensis* using DNAsecure Plant Kit (TIANGEN). The isolated DNA was quantified using a NanoDrop D-100 spectrometer (Nanodrop Technologies) according to the manufacturer’s protocol. A high-quality genome assembly for the *C. praecox* genome was achieved by using three sequencing methods that include Illumina paired-end and mate-pair sequencing, 10X Genomics sequencing, and single molecule real-time (SMRT) sequencing from Pacific Biosciences (PacBio).

A total of 10 μg of sheared DNA was used for a 20-kb insert size library subsequently sequenced on the Pacbio Sequel platform. A short-read genomic library was prepared using a Illumina TruSeq library construction kit according to the manufacturer’s instructions (Illumina, San Diego, CA). A total of 6 libraries with insert sizes ranging from 250 bp to 20 kb were constructed for *C*. *chinensis* and one library with a 350-bp insert size was constructed for *C. praecox*. The libraries were then sequenced on the Illumina HiSeqXTen platform. For the 10X Genomics library, about 1 ng input DNA with an average 50 kb length was used for the GEM reaction procedure during PCR, and 16-bp barcodes were introduced into droplets. The droplets were then fractured followed by final purification of the DNA library. A total of six libraries were finally sequenced on the Illumina HiseqXTen.

### Genome assembly and assessment of the assembly quality

De novo assembly of the PacBio reads was performed using the FALCON assembler [18] (https://github.com/PacificBiosciences/FALCON/). Before assembly, we used FALCON to correct the PacBio reads and then assemble them into contigs with parameters (length_cutoff_pr = 4000, max_diff = 100, max_cov = 100). This resulted in primary contigs (p-contigs) that were then polished using Quiver [58] by aligning SMRT reads. Pilon was used to perform the second round of error correction with short paired-end reads generated from Illumina HiSeq Platforms. For the scaffolding step, Long Ranger (version 2.1.2) (https://support.10xgenomics.com/genome-exome/software/pipelines/latest/installation) was first used to build scaffolds using 10X data. FragScaff (version 1.1) [59] was further applied to build super-scaffolds using the barcoded sequencing reads.

We applied both CEGMA (Core Eukaryotic Gene Mapping Approach) [60] and BUSCO (V3, Benchmarking Universal Single-Copy Orthologs) [61] to evaluate the completeness of the assembly.

For the assembly of *C. chinensis*, we first used SOAPdenovo [62] to filter duplicates, adaptor contamination, and low-quality bases from Illumina PE reads before assembling them into preliminary scaffolds. Then GapCloser (version 1.12) [63] from the SOAPdenovo package was used for gap filling within assembled scaffolds using all pair-end reads. Lastly, SSPACE [64] was used to improve the assembled genome.

### Chromosome assignment using Hi-C

One Dovetail Hi-C library was prepared in a similar manner as described previously [65]. The library was sequenced on an Illumina HiSeq platform. The number and length of read pairs produced for the library was 431 million and 2 × 100 bp respectively. The Dovetail Hi-C library reads provided 93.06 × physical coverage of the genome (1–50 kb pairs). The Hi-C sequencing data was aligned to the assembled contigs with BWA-mem [66], and then clustered onto chromosomes with LACHESIS (http://shendurelab.github.io/LACHESIS/).

### Repeat and non-coding RNA annotation

We used two complementary methods (one homology-based and de novo-based) to discover and classify transposable elements (TEs). The homology-based repeat library was generated from a known repeat library (Repbase 15.02) using RepeatMasker (version 3.3.0) [67]. RepeatModeler (Vision 1.0.5) (http://www.repeatmasker.org/), RepeatScout [68], Piler, and LTR_FINDER [69] were used to build the de novo-based repeat library. RepeatProteinMask was performed to detect TEs in the *C. praecox* and *C. chinensis* genome by comparing the TE protein database. The integrated repeat library was finally annotated by the Tandem Repeats Finder (TRF) [70]. The tRNA genes were predicted using tRNAscan-SE software [71]. The rRNA, miRNA, and snRNA fragments were predicted using INFERNAL software [72] with searches against the Rfam database (release 9.1) [73].

### Structural and functional annotation of genes

We adapted a combination of three strategies that include (1) de novo predictions, (2) homolog-based predictions, and (3) RNA-seq-based predictions to annotate the protein-coding genes in the *C. praecox* and *C. chinensis* genomes. De novo predictions were carried out using five ab initio gene prediction programs that include Augustus (version 3.0.2) [74], Genscan (version 1.0) [62], Geneid [75], GlimmerHMM (version 3.0.2) [76], and SNAP [77]. For the homolog-based predictions, the protein sequences of six species namely *Arabidopsis thaliana*, *Oryza sativa*, *Nelumbo nucifera*, *Beta vulgaris*, *Solanum lycopersicuma*, and *Vitis vinifera* were aligned against the repeat-masked genome using TBLASTN [78] with a cutoff *E*-value of 10^− 5^. Genewise (version 2.2.0) [79] was employed to predict gene models based on the alignment sequences. We used two methods to achieve RNA-seq-based predictions. One was mapping the RNA-seq data to the *C. praecox* and *C*. *chinensis* genome and assembling the transcripts using Tophat (version 2.0.8) [80] and cufflinks (version 2.1.1) (http://cufflinks.cbcb.umd.edu/) [81]. The other was applying Trinity [82] to assemble the RNA-seq data followed by the PASA software (http://pasapipeline.github.io/) [83] to improve the gene structures. To finalize the gene set, all the predictions were combined using EVidenceModeler (EVM) [84] to produce the non-redundant gene sets.

Functional annotation of protein-coding genes was carried out by performing BLASTP (*E*-value ≤1e^− 5^) searches against SwissProt (http://www.uniprot.org/), TrEMBL [85], and NCBI non-redundant (NR) protein databases. Motifs and domains were annotated by using InterProScan (version 4.7) [86] to search against InterPro (v29.0) databases which include Pfam, PRINTS, PROSITE, ProDom, and SMART. The GO term [87] for each gene was achieved from the corresponding InterPro descriptions. Additionally, the gene set was mapped to the KEGG (release 53) [88] pathway database to identify the best match classification for each gene.

### Gene family and phylogenomic analysis

To identify gene family groups, we analyzed protein-coding genes from 17 species, *Amborella trichopoda*, *Ananas comosus*, *A. thaliana*, *Camellia sinensis, C. kanehirae*, *Daucus carota*, *Elaeis guineensis*, *L. chinense*, *Musa acuminata*, *N. nucifera*, *O. sativa*, *Papaver somniferum*, *Phalaenopsis equestris*, *S. lycopersicum*, *V. vinifera*, *C. praecox*, and *C*. *chinensis* genomes. Orthologous gene groups of *C. praecox* and 16 other species were identified by running OrthoMCL program (http://orthomcl.org/orthomcl/) [23]. We determined the expansion and contraction of the gene families by comparing the cluster size differences between the ancestor and each species using the CAFÉ program [89].

To infer the phylogenetic placements of wintersweet, two sets of single-copy genes (SSCG and OSCG) were identified using SonicParanoid [24] v.1.0 and OrthoMCL [23] v.2.0.9 from 17 seed plants respectively. For each gene, amino acid sequences were aligned using MUSCLE [90]. For each dataset SSCG and OSCG, the phylogenetic trees were constructed by both coalescent and concatenation approaches. For the coalescent approach, gene trees were inferred by IQ-TREE [91] v.1.6.9; these gene trees were then used by ASTRAL [25] v.5.6.1 to construct species trees. The quartet score was estimated for each node showing quartet support for the species tree. For the concatenation-based analyses, the ML trees were inferred from the concatenated amino acid sequences using IQ-TREE [91] v.1.6.9 with ultrafast bootstrap testing (1000 replicates) [92]. For plastid genes, the extrons of 38 single-copy genes were extracted from 26 plastid genome sequences. We aligned these genes with MUSCLE [90]. The concatenated amino acid sequences of these genes were used to infer the ML trees with RAxML [93]. The mcmctree program of PAML (http://abacus.gene.ucl.ac.uk/software/paml.html) [94] was applied to estimate divergence time among 17 species with main parameters (burn-in = 10,000, sample-number = 100,000, and sample-frequency = 2). Four calibration points were selected from articles and TimeTree website (http://www.timetree.org) as normal priors to restrain the age of the nodes, such as 105–115 Mya between *V. vinifera* and *A. thaliana*, 110–124 Mya between *C. sinensis* and *A. thaliana*, 148–173 Mya between *O. sativa* and *A. thaliana*, and 94–115 Mya between *A. comosus* and *O. sativa*.

### Analysis of genome synteny and whole-genome duplication

We performed synteny searches to identify syntenic blocks within *C. praecox* and between *C. praecox* and *Amborella* using MCScanX [95]. Synonymous substitutions per synonymous site (*K*s) between colinear genes were estimated using the codeml approach as implemented in the PAML package [94]. Calculation and correction of Ks of collinear blocks with reference to Prickly waterlily and rigid hornwort genome [17] and Paleotetraploidization study in Cucurbitaceae [96] as follows: Firstly, the median Ks values were selected to represent each syntenic block, and the probability density distribution curve of Ks was estimated using MATLAB with the kernel smoothing density function. Multipeak fitting of the curve was performed using the Gaussian approximation function (cftool) in MATLAB. Secondly, based on Ks distribution of *Liriodendron* and *Cinnamomum* described in Prickly waterlily and rigid hornwort genome [17], the Ks correction coefficient of species of *Magnolia* were calculated and then we got the corrected Ks rate. The Dot plots between *C. praecox* and *C. kanehirae* as well as the *L. chinense* genome was generated with Quota synteny alignment software [97] to visualize the paleopolyploidy level of *C. praecox* in relation to *C. kanehirae* and *L. chinense*.

### Ancestral genome reconstruction

The ancestral genome of Laurales was reconstructed according to the procedure (Additional file 2: Fig. S15) as described previously [98, 99]. The ancestral karyotype of *C. praecox*, *C. chinensis*, and *C. kanehirae* was first determined by genome alignments using cumulative identity percentage (CIP) and cumulative alignment length percentage (CALP) BLAST parameters [100]. The coordinate of conserved genes among the three genomes were extracted from these alignments that belong to syntenic blocks identified by McscanX [95]. Then the syntenic blocks were merged using GRIMM [101] to get the coordinate correspondence between the three collinear groups. Finally, the MGR [102] software was used to rearrange the multiple genomes to get the ancestral gene orders.

### Comparative analysis of LTR retrotransposons

Intact long terminal repeat retrotransposons (LTR) were identified by searching the genomes of *C. praecox* with LTRharvest [103] (-motif tgca -motifmis 1) and LTR_Finder [69] (LTR length 100 to 5000 nt; length between two LTRs: 1000 to 20,000 nt). A two-step procedure was used to filter the candidate sequences to reduce false positives. First, the primer binding site (PBS) motif was identified by LTR digest [104] based on the predicted tRNA sequences from tRNAscan-SE [72], and only elements that contained PBS were retained; next, protein domains (pol, gag, and env) in candidate LTR retrotransposons were identified by searching against HMM profiles collected by Gypsy Database (GyDB) (http://gydb.org/). Elements containing a gag domain, protease domain, reverse transcriptase (RT) domain, and integrase domain were considered as intact. Second, families of these intact LTR retrotransposons were clustered using the SiLiX software package (lbbe.univ-lyon1.fr/SiLiX) [105]. Finally, LTRs that did not contain protein domains or that belonged to families with less than 2 members were discarded. Each nucleic acid diversity (*λ*) was calculated from the MUSLCE alignment of the 5′and 3′ LTR sequences of LTR-RTs by EMBOSS program distmat. Using a substitution rate (*r*) of 1.51 × 10^− 9^ substitutions per site per year [14], the insertion date (*T*) was computed for each LTR-RT (*T* = *K*/2*r*, *K*: genetic distance, *K* = − 0.75ln(1 − 4*λ*/3)).

The sequences of intact LTR-RT identified in *C. praecox*, *C. chinensis*, *C. kanehirae*, and *L. chinense* genomes were translated according to 3-frames. The translated amino acid sequences were then searched for the *Ty1/Copia* (PF07727) and *Ty3/Gypsy* (PF000078) domains using HMMER (version 3.1b2, http://hmmer.org) (E-value ≤1e^− 5^). The target hit amino acid sequences of *Copia* and *Gypsy* superfamilies were aligned using mafft (https://mafft.cbrc.jp/alignment/software/) with default parameters. The phylogenetic trees of *Copia*-like and *Gypsy*-like LTR-RTs were constructed using fastTree (http://www.microbesonline.org/fasttree/).

### Histological examination

To investigate the floral developmental characteristics of wintersweet, we performed a detailed developmental study from the initiation to the opening of the flower bud. Data collection was initiated on the day that the floral buds were visible to the naked eye. The sampling of *C. praecox* began on April 11, 2016, and ended on February 6, 2017. Ten axillary buds on current-year-old short branches were collected at random every 7 days from three trees. Five homogenized buds were used for histological examination and the others were frozen in liquid nitrogen and stored at − 80 °C for further RNA sequencing.

Flower buds at various stages of development were fixed in FAA (70% ethanol: formalin: acetic acid = 5:5:90) for more than 24 h. Then, 8-μm-thick microtome sections were made after embedding in paraffin wax. These sections were mounted on slides and stained with 1% safranine and 0.5% fast green. Slides were visually examined and photographed using a Nikon Eclipse 80i digital imaging microscope equipped with an electronic photo system. Based on the observation and the annual morphological changes of flower buds, we divided the flower developmental process into nine stages (FBS1–9) (Fig. 4a).

### RNA isolation, library construction, and sequencing

The flower buds or whole flowers with three biological replicates were harvested at 14 stages including FBS1–9 and S1–5 (Fig. 4 and Additional file 2: Fig. S18) respectively. Total RNA from flower buds or whole flowers at different stages was extracted using an RNA prep pure Plant RNA Purification Kit (Tiangen Biotech, Beijing, China). RNA quality was verified using an Agilent 2100 (Agilent Technologies, CA, USA) plus electrophoresis system. A total of 42 cDNA libraries were prepared and sequenced on an Illumina HiSeq 2500 platform using paired-end cycles.

### Analysis of genes involved in the formation of floral scent

Using terpene and phenylpropanoid biosynthesis pathway genes in *A. thaliana* [43] and *Petunia hybrida* [45] as bait, the corresponding genes in *C. praecox* were identified based on genome annotation and local blast search against wintersweet genome with a filtered parameter (*E*-value < 10^− 5^, identity ≥50%, and coverage ≥30%). For larger-size gene families, the genes were determined by combining local blast reports and phylogenetic analysis to distinguish the orthologs of corresponding functionally characterized genes.

To identify the *CpTPS* genes in *C. praecox* and *C. chinensis*, two Pfam domains: PF01397 and PF03936 were used to search against the proteome using HMMER [106]. The target hits (*E*-value < 10^− 5^) were identified as candidate terpene synthase genes. Synteny and collinearity between these genes were analyzed using MCScanX [95]. Based on the chromosome location in the gff files, the structure and chromosomal distribution of *CpTPS* genes were illustrated with TBtools [107]. The 2000-bp region upstream of *CpTPS* genes was defined as the promoter region, in which the cis-acting regulatory elements were identified with PlantCARE [108] and PLACE [109] databases. A heat map was generated with the TBtools [107] based on the expression level of wintersweet *TPS* genes at different floral developmental stages. *A. thaliana* and *O. sativa TPSs* were downloaded from phytozome (https://phytozome.jgi.doe.gov/pz/portal.html). All the phylogenetic trees of functional genes were constructed by maximum likelihood method with MEGAX [110] using the amino acid sequence alignments generated by ClustalX [111].

### Characterization of the *TPS* genes

The ORF of the three *TPS* genes (*CpTPS4*, *CpTPS9*, and *CpTPS42*) were isolated and subcloned into the BamHI/XhoI sites of pET28a using ClonExpress II One Step Cloning Kit (http://www.vazymebiotech.com/index.html) according to the manufacturer’s introductions. The recombinant plasmids and pET28a lacking an insert (control) were transformed into *E. coli* BL21 (DE3) competent cells. After 0.5 mM IPTG induction at 16 °C overnight, the proteins were harvested and purified by affinity chromatography on nickel-nitrilotriacetic acid-agarose (Qiagen, http://www.qiagen.com). The purity of the isolated proteins was verified by the densitometry of the SDS-PAGE gels after Coomassie Brilliant Blue staining. The concentration of purified proteins was determined by the Bradford method [112].

Assays for TPS protein activity were carried out in a 1 ml assay buffer (30 mM HEPES, pH 7.5, 5 mM DTT, 25 mM MgCl_2_) containing 10 μg purified CpTPS proteins and 60μΜ GPP/FPP. The mixture was incubated at 30 °C for an hour and then 45 °C for 15 min. After incubation, the synthesized volatiles were collected using the DVB/CAR/PDMS headspace sampler and analyzed as Additional file 3: Supplementary Note 2.

## Supplementary information

**Additional file 1: Table S1.** Summary of sequencing data of *Chimonanthus praecox* and *Calycanthus chinensis* genome. Table S2. Estimation of genome size of *Chimonanthus praecox* and *Calycanthus chinensis* using K-mer analysis. Table S3. Statistic of *Chimonanthus praecox* and *Calycanthus chinensis* genome assembly. Table S4. Chromosomes length of wintersweet using HiC reads. Table S5. Assembly results of wintersweet using Hi-C. Table S6. Validation of genome assembly using BUSCO and CEGMA method. Table S7. Statistics of predicted protein-coding genes in *Chimonanthus praecox* and *Calycanthus chinensis* genome. Table S8. Functional annotation of the wintersweet protein-coding genes. Table S9. Annotation of conserved non-coding RNA genes in the wintersweet genome. Table S10. Wintersweet-specific gene families. Table S11. The list of expansion genes in wintersweet genome. Table S12. Annotation of repeat sequences in the wintersweet genome. Table S13. List of flowering-time and floral organ identity gene candidates in wintersweet. Table S14. The upregulated expressed genes involved in flowering networks in FBS1 stage compared with FBS2 stage. Table S15. Monthly temperatures in Wuhan (March 2017–February 2018). Table S16. Differently expressed genes between FBS6 and FBS7 stage. Table S17. List of Heat stress transcription factors (Hsf) and DEHYDRATION- RESPONSIVE ELEMENT BINDING PROTEIN (DREB) gene candidates identified in the wintersweet genome. Table S18.The candidate genes involved in the terpene synthesis pathways in wintersweet. Table S19. Terpene synthase family in wintersweet. Table S20. The candidate genes involved in the benzenoid/ phenylpropanoid synthesis pathways in wintersweet. Table S21. The list of transcription factor candidates involved in the regulation of VOCs synthesis in wintersweet. Table S22. Primers for *CpTPS* genes coloning and MADS-box B&C class genes Real-Time PCR. Table S23. Transporter candidates involved in benzenoid/phenylpropanoid synthesis pathways in wintersweet genome. Table S24. Tandem duplicated gene clusters in wintersweet genome.

**Additional file 2: Fig. S1.** Evaluation of Chimonanthus praecox genome by k-mer analysis and cell flow cytometry. Fig. S2. Hi-C map of the wintersweet genome showing genome-wide all-by-all interactions. Fig. S3. Chromosome biology of *Chimonanthus praecox* (a) and *Calycanthus chinensis*. Fig. S4. Annotation of the wintersweet-specific and expanded genes. Fig. S5. An uneven TE distribution across wintersweet genome and TE distribution in genic regions. Fig. S6. LTR insertion time estimation. Fig. S7. Phylogenetic analysis of wintersweet LTR retrotransposons. Fig. S8. Concatenated- and ASTRAL-based phylogenetic trees. Fig. S9. The phylogenetic tree based on 38 chloroplast genes from 26 species. Fig. S10. A phylogenetic tree of 29 plant species based on 2420 concatenated genes trees using RAxML. Fig. S11. Types of gene duplication in the wintersweet genome. Fig. S12. Duplications of genomic paralogous genes in wintersweet. Fig. S13. Distribution of synonymous substitution levels (Ks) of syntenic orthologous (solid curves) and paralogous genes (dashed curves). Fig. S14. Topologies of gene trees depicting the two possible scenarios of speciation among wintersweet and *Liriodendron.* Fig. S15. A modified pipeline of ancestral genome reconstruction. Fig. S16. Phylogenetic analysis of FT-like and SVP-like and the analysis of bud break and cell division (*CpCDs*) related genes and genes involved in phytohormone-related pathways. Fig. S17. Gas chromatogram of glycosidic floral volatiles from the flowers of wintersweet. Fig. S18. Changes of the major floral volatiles during flower development in wintersweet. Fig. S19. Subcellular location of CpTPS proteins. Fig. S20. Cluster and STEM analysis of differentially expressed TFs during flower development. Fig. S21. Promoter cis-element analysis of 52 *CpTPSs*. Fig. S22. Analysis of benzenoid carboxyl methyltransferases (*BCMTs*) and cell division (*CpCDs*) related genes. Fig. S23. Gene structure and classification of putative *CpTPSs*. Fig. S24. Annotation of the Tandem duplicated genes.

**Additional file 3: Supplementary Note 1.** Estimation of Genome Size and Chromosome Number Assessment; Identification of Orthologs of Flowering Time Genes; Identification of MADS-box Genes; Genes Expression Analysis. Supplementary Note 2. Headspace Collection and GC–MS Analyses of Floral Volatiles; Changes of volatile compounds during flower development; Phylogenetic and Structural Analysis of TPS Family; Subcellular Location of the fused *CpTPS4/9/42*-fused green Fluorescent Protein; Construction of *CpTPS4* Overexpressed Tobacco Plants, Analysis of Transcription of Factors; Analysis of the genes involved in terpene and benzenoid biosynthesis transport. Supplementary Note 3. Tandem duplication analysis; The order of speciation and WGD event in *Liriodendron* and wintersweet lineage.

**Additional file 4.** Review history.

## Data Availability

All raw sequencing reads have been deposited in the NCBI Sequence Read Archive (https://www.ncbi.nlm.nih.gov/sra) under project PRJNA600650 [113].
