# Supplementary material for: The chromosome-level wintersweet (Chimonanthus praecox) genome provides insights into floral scent biosynthesis and flowering in winter
Source: Genome Biol. 2020 Aug 10;21:200. doi: 10.1186/s13059-020-02088-y (PMC7419205; doi:10.1186/s13059-020-02088-y)
Supplement: Supplementary file 2 — Additional file 2: Fig. S1. Evaluation of Chimonanthus praecox genome by k-mer analysis and cell flow cytometry. Fig. S2. Hi-C map of the wintersweet genome showing genome-wide all-by-all interactions. Fig. S3. Chromosome biology of Chimonanthus praecox (a) and Calycanthus chinensis. Fig. S4. Annotation of the wintersweet-specific and expanded genes. Fig. S5. An uneven TE distribution across wintersweet genome and TE distribution in genic regions. Fig. S6. LTR insertion time estimation. Fig. S7. Phylogenetic analysis of wintersweet LTR retrotransposons. Fig. S8. Concatenated- and ASTRAL-based phylogenetic trees. Fig. S9. The phylogenetic tree based on 38 chloroplast genes from 26 species. Fig. S10. A phylogenetic tree of 29 plant species based on 2420 concatenated genes trees using RAxML. Fig. S11. Types of gene duplication in the wintersweet genome. Fig. S12. Duplications of genomic paralogous genes in wintersweet. Fig. S13. Distribution of synonymous substitution levels (Ks) of syntenic orthologous (solid curves) and paralogous genes (dashed curves). Fig. S14. Topologies of gene trees depicting the two possible scenarios of speciation among wintersweet and Liriodendron. Fig. S15. A modified pipeline of ancestral genome reconstruction. Fig. S16. Phylogenetic analysis of FT-like and SVP-like and the analysis of bud break and cell division (CpCDs) related genes and genes involved in phytohormone-related pathways. Fig. S17. Gas chromatogram of glycosidic floral volatiles from the flowers of wintersweet. Fig. S18. Changes of the major floral volatiles during flower development in wintersweet. Fig. S19. Subcellular location of CpTPS proteins. Fig. S20. Cluster and STEM analysis of differentially expressed TFs during flower development. Fig. S21. Promoter cis-element analysis of 52 CpTPSs. Fig. S22. Analysis of benzenoid carboxyl methyltransferases (BCMTs) and cell division (CpCDs) related genes. Fig. S23. Gene structure and classification of putative CpTPSs. Fig. S24. A [file 13059_2020_2088_MOESM2_ESM.docx]

**The chromosome-level wintersweet (*Chimonanthus praecox*) genome provides insights into floral scent biosynthesis and flowering in winter**

**Authors:**

Junzhong Shang^1,#^, Jingpu Tian^3, #^, Huihui Cheng^6,#^, Qiaomu Yan^1^, Lai Li^1^, Abbas Jamal^1^, Zhongping Xu^4,5^, Lin Xiang^1^, Christopher A Saski^7^, Shuangxia Jin^4,5^*, Kaige Zhao^1^*, Xiuqun Liu^1^*, Longqing Chen ^2^*

**Authors affiliations:**

^1^Key Laboratory of Horticultural Plant Biology, Ministry of Education, Huazhong Agricultural University, Wuhan, Hubei, 430070, P. R. China

^2^Southwest Engineering Technology and Research Center of Landscape Architecture, State Forestry Administration, Southwest Forestry University, Kunming, Yunnan, 650224, P. R. China

^3^School of Architecture and Art Design，Hunan University of Science and Technology，Xiangtan, Hunan, 411201, P. R. China

^4^National Key Laboratory of Crop Genetic Improvement, Huazhong Agricultural University, Wuhan, Hubei, 430070, P. R. China

^5^Xinjiang Production and Construction Corps Key Laboratory of Protection and Utilization of Biological Resources in Tarim Basin, Tarim University, Alaer, Xinjiang 843300, China

^6^Novogene Bioinformatics Institute, Beijing, 100083, P. R. China

^7^Department of Plant & Environmental Science, Clemson University, SC 29631 USA


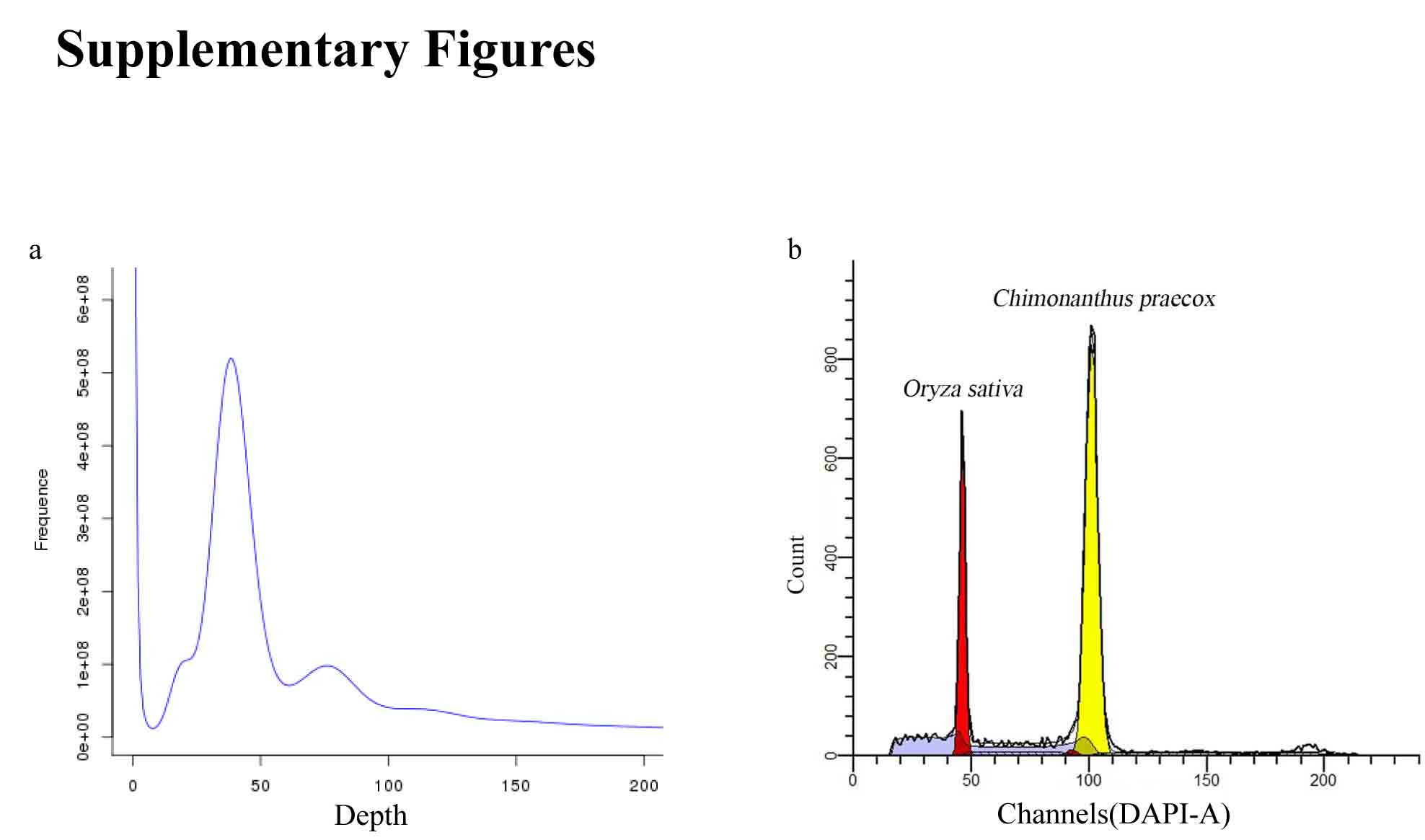


**Fig. S1: Evaluation of *Chimonanthus praecox* genome by k-mer analysis and by cell flow cytometry.** (a) Graph of k-mer distribution, X axis shows k-mer depth and Y axis shows k-mer frequency. The genome size was measured as 796.52Mb. (b) Relative DNA content measured by cell flow cytometry. The X axis shows the relative DNA content and the Y axis shows the strength of fluorescence signal calculated as the number of events. We used the *Oryza sativa* genome (389Mb) as a reference. The genome size of wintersweet was caculated as 805.88 Mb.


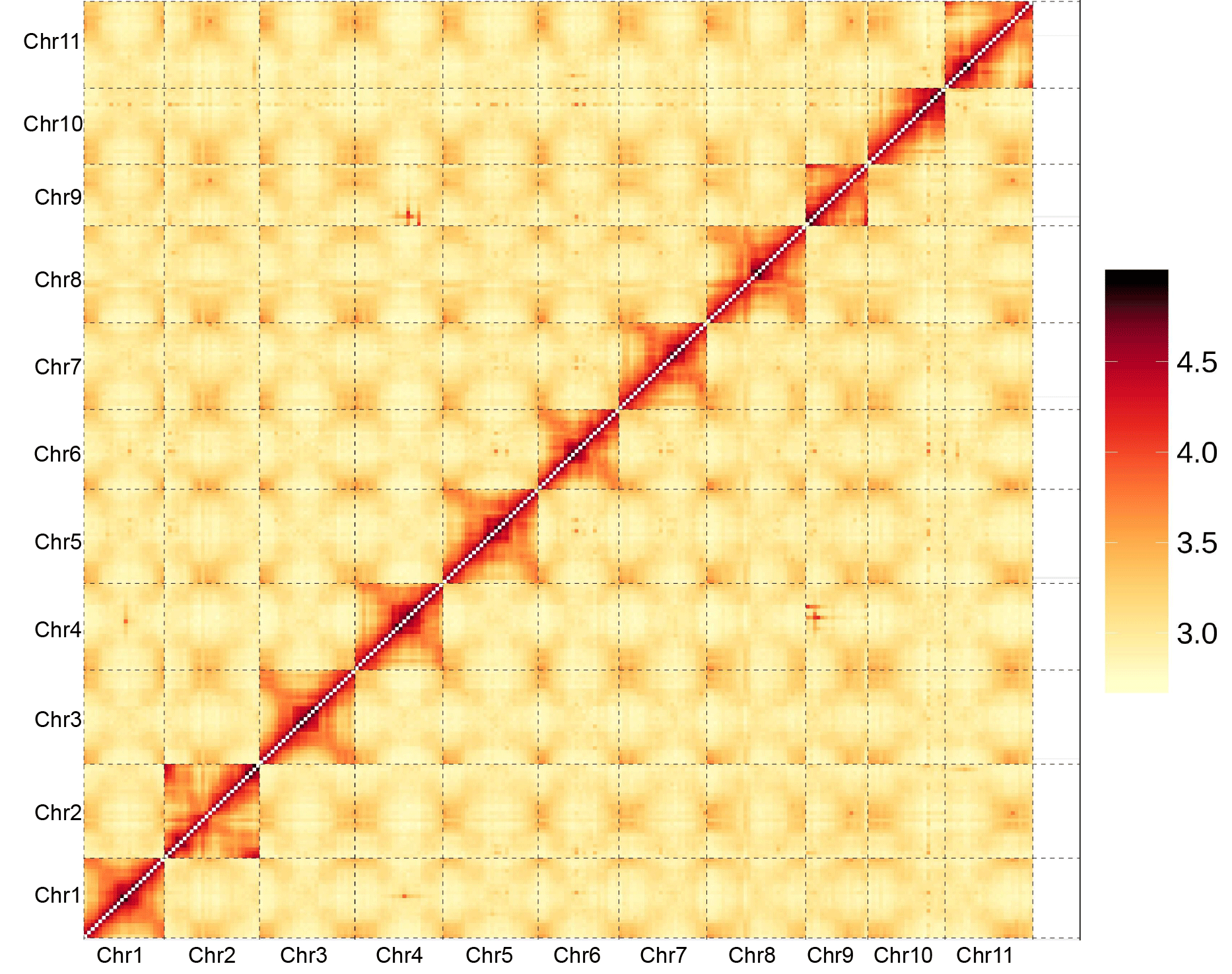


**Fig. S2: Hi-C map of the wintersweet genome showing genome-wide all-by-all interactions**. The map shows a high resolution of individual chromosomes that are scaffolded and assembled independently.


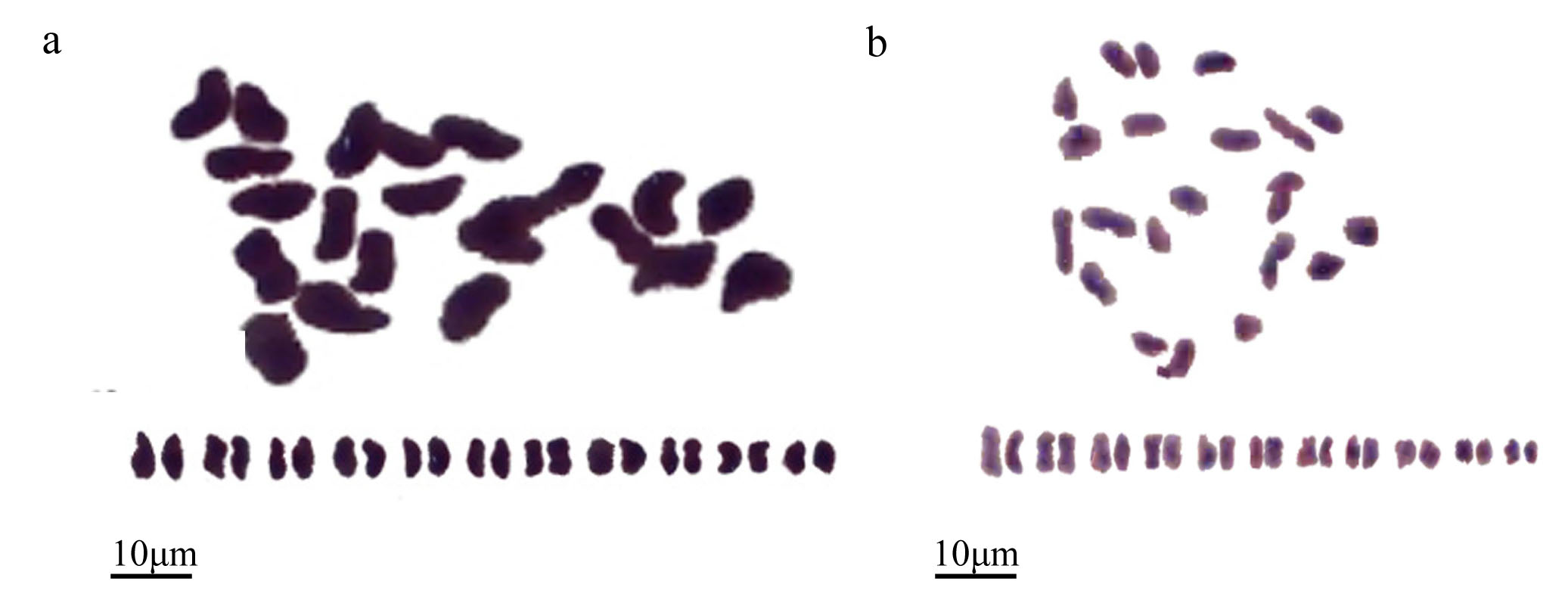


**Fig. S3: Chromosome biology of *Chimonanthus praecox* (a) and *Calycanthus chinensis* (b).** Basic fuchsin-stained young leaves showing the chromosome number (2n = 22). Three independent staining and counts were carried out.


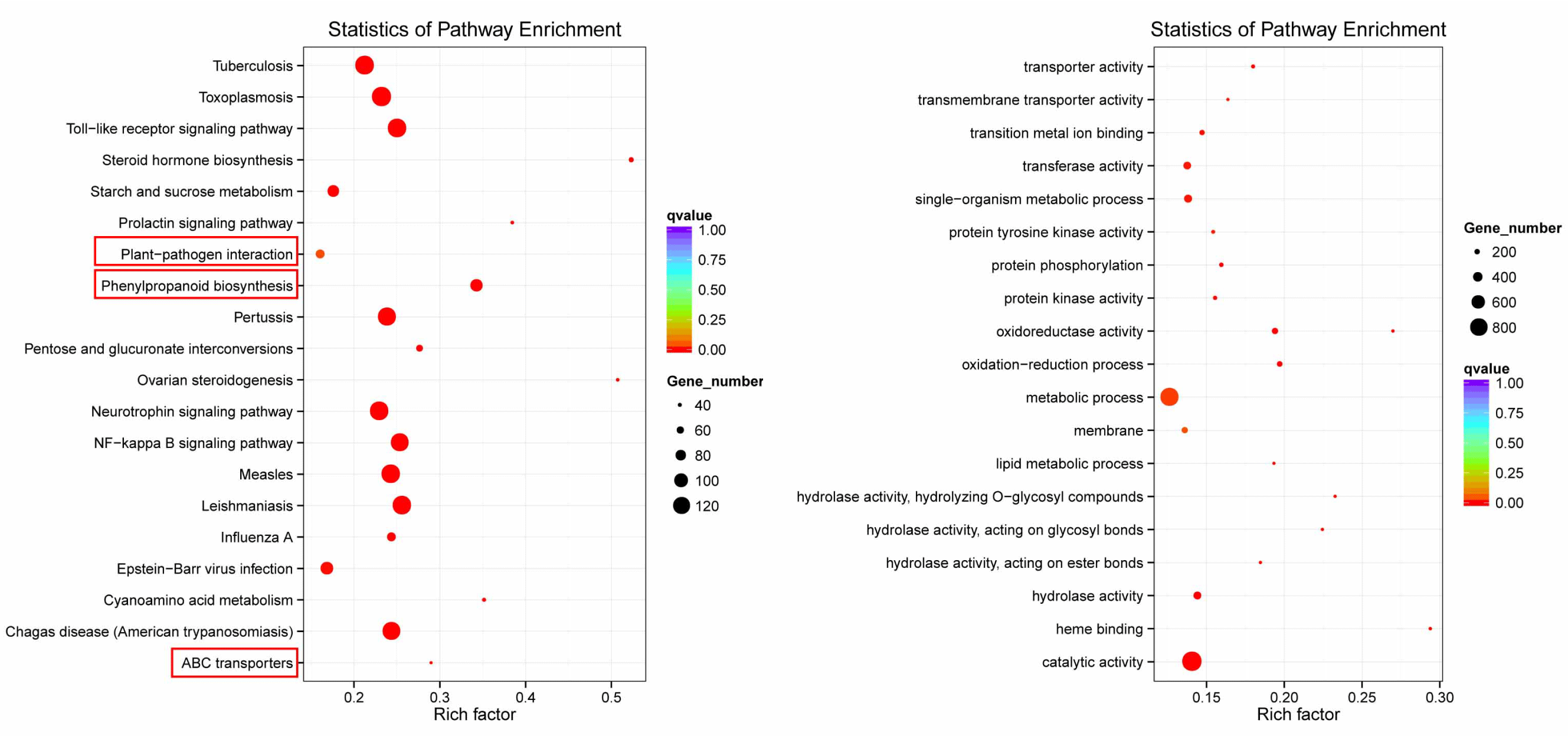


**Fig. S4: Annotation of the wintersweet-specific and expanded genes.** (a) KEGG pathway enrichment distribution of the expanded genes in wintersweet genome. (b) GO functional enrichment of the wintersweet-specific genes .


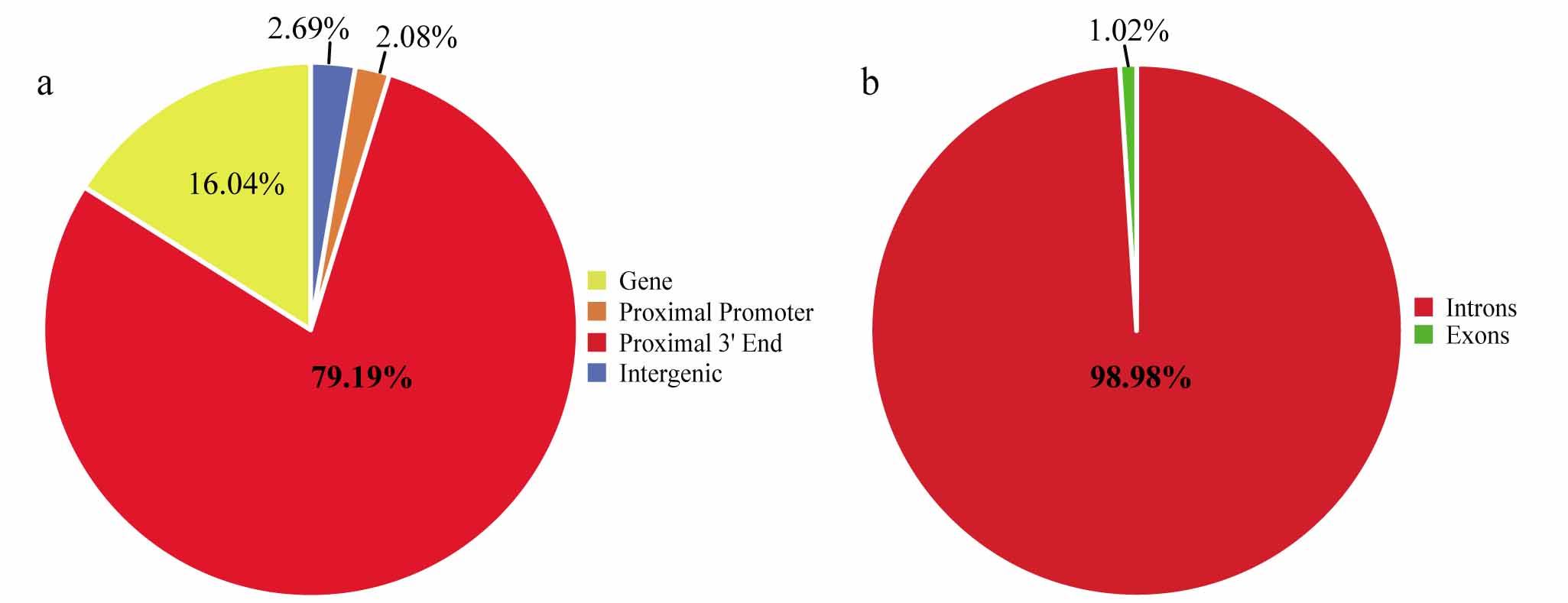


**Fig. S5: An uneven TE distribution across wintersweet genome and in genic regions.** (a) The pie graph demonstrates the proportion of TEs accounting for the total TEs present in the four separate wintersweet genomic regions: gene (yellow), proximal promoter (orange), proximal 3' end (red) and intergenic regions (blue) TEs. (b) Among the TEs present in genic regions, 1.02% located in exons and 98.98% in introns.


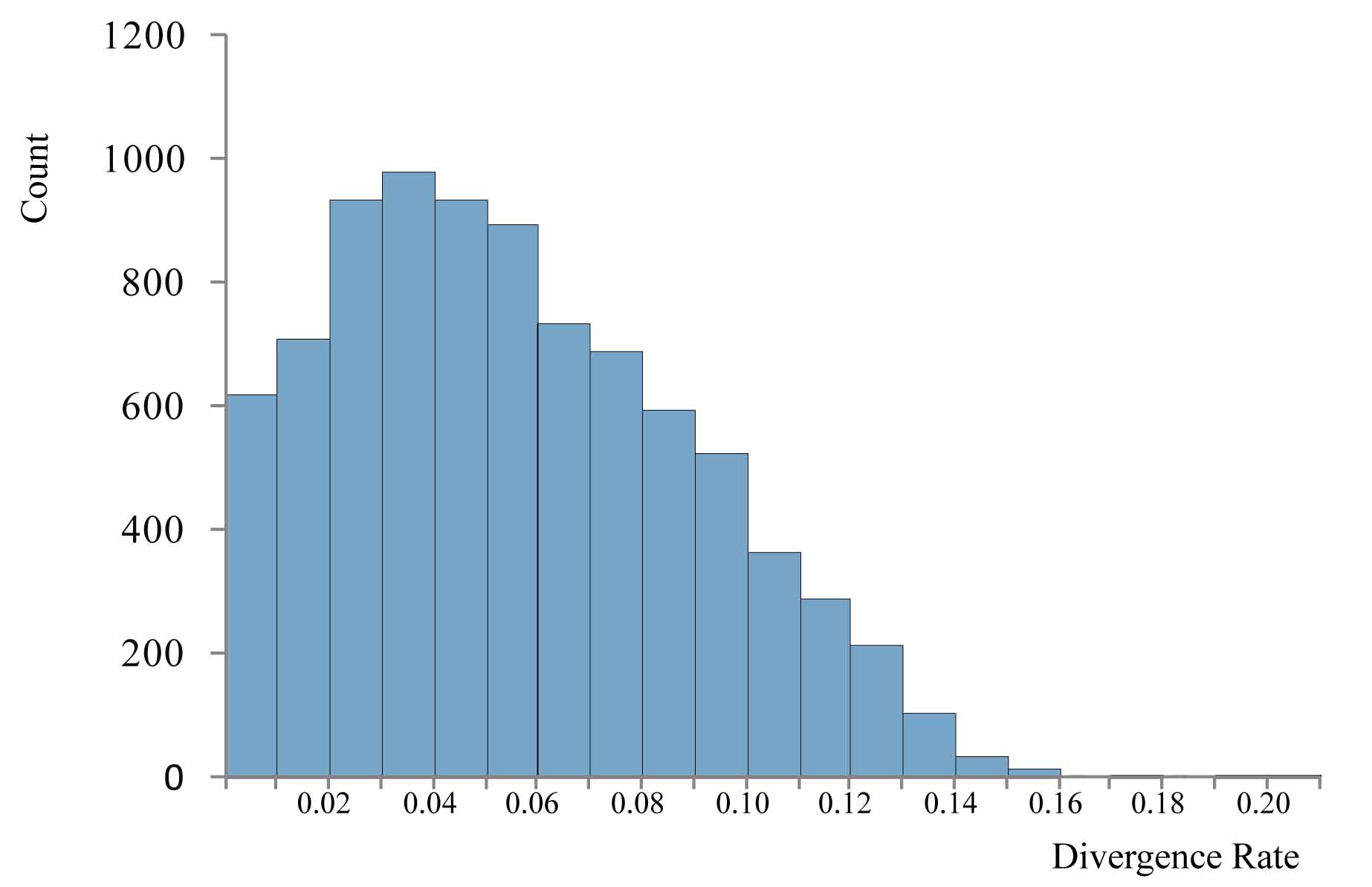


**Fig. S6: LTR insertion time estimation.** Ks distributions of the complete LTR in the wintersweet genome are plotted by a window of 0.01.


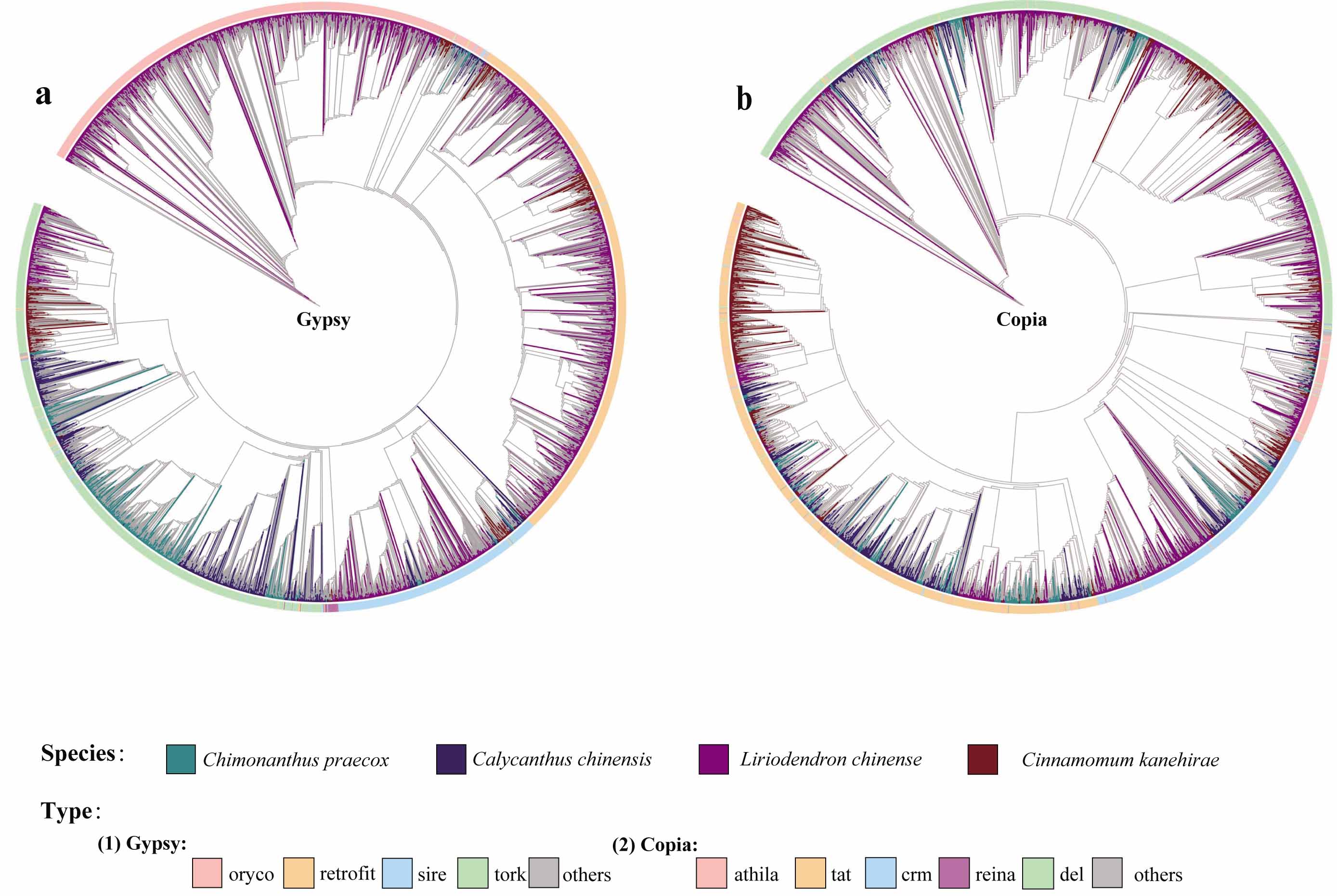


**Fig. S7: Phylogenetic analysis of wintersweet LTR retrotransposons.** The unrooted phylogenetic tree of *Gypsy* (a) and *Copia* (b) elements was constructed on the basis of the reverse-transcriptase domain sequences.


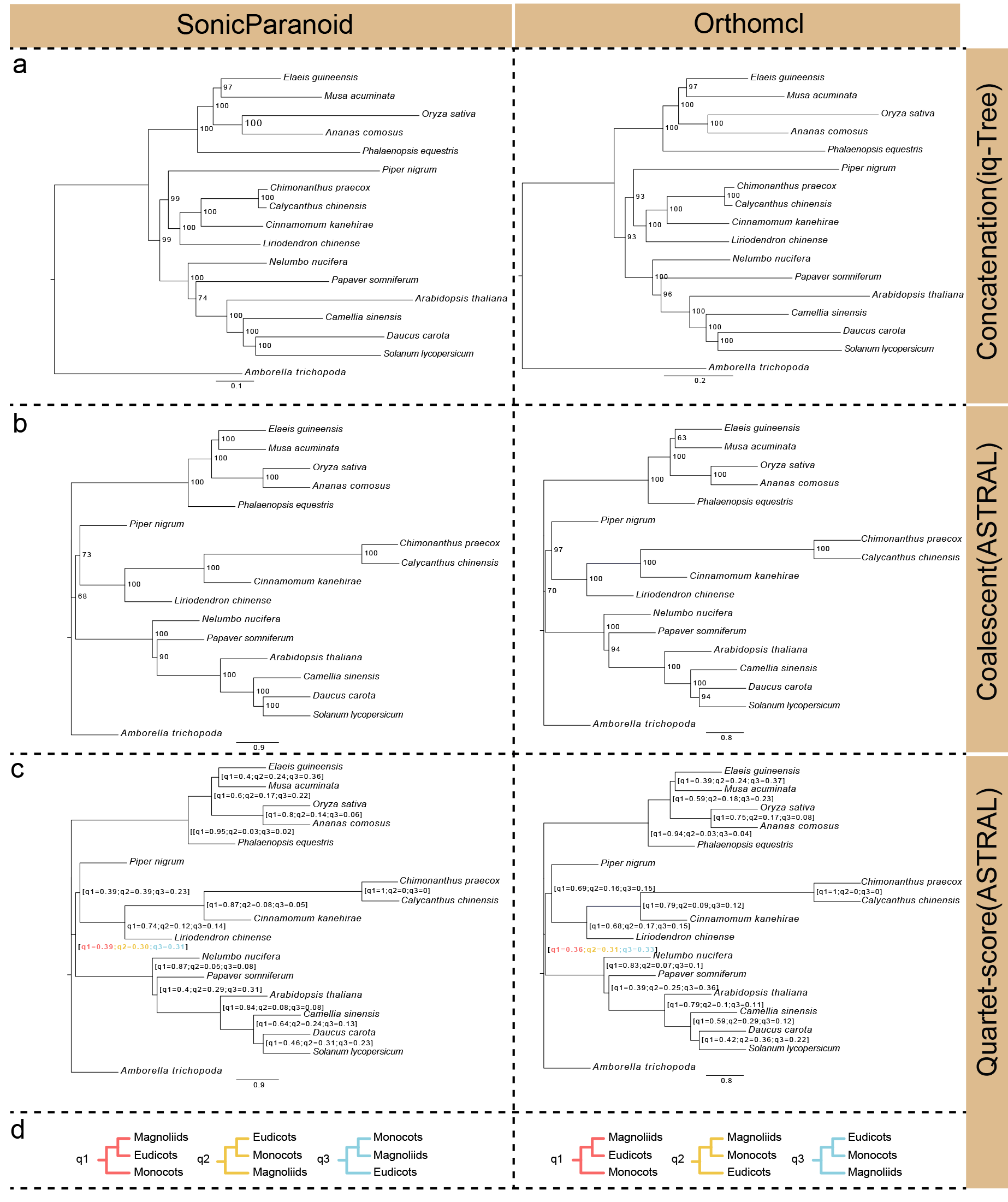


**Fig. S8: Concatenated- and ASTRAL-based phylogenetic trees.** Phylogenetic trees based on the concatenated (a) and multi-species coalescent methods (b) using amino acid sequences. Estimated proportions of the SSCG and OSCG gene trees with different topologies (c) based on amino acid alignments. The q1, q2, and q3 refer to the quartet support for the main topology (red), the first alternative (yellow), and the second alternative (blue), respectively.


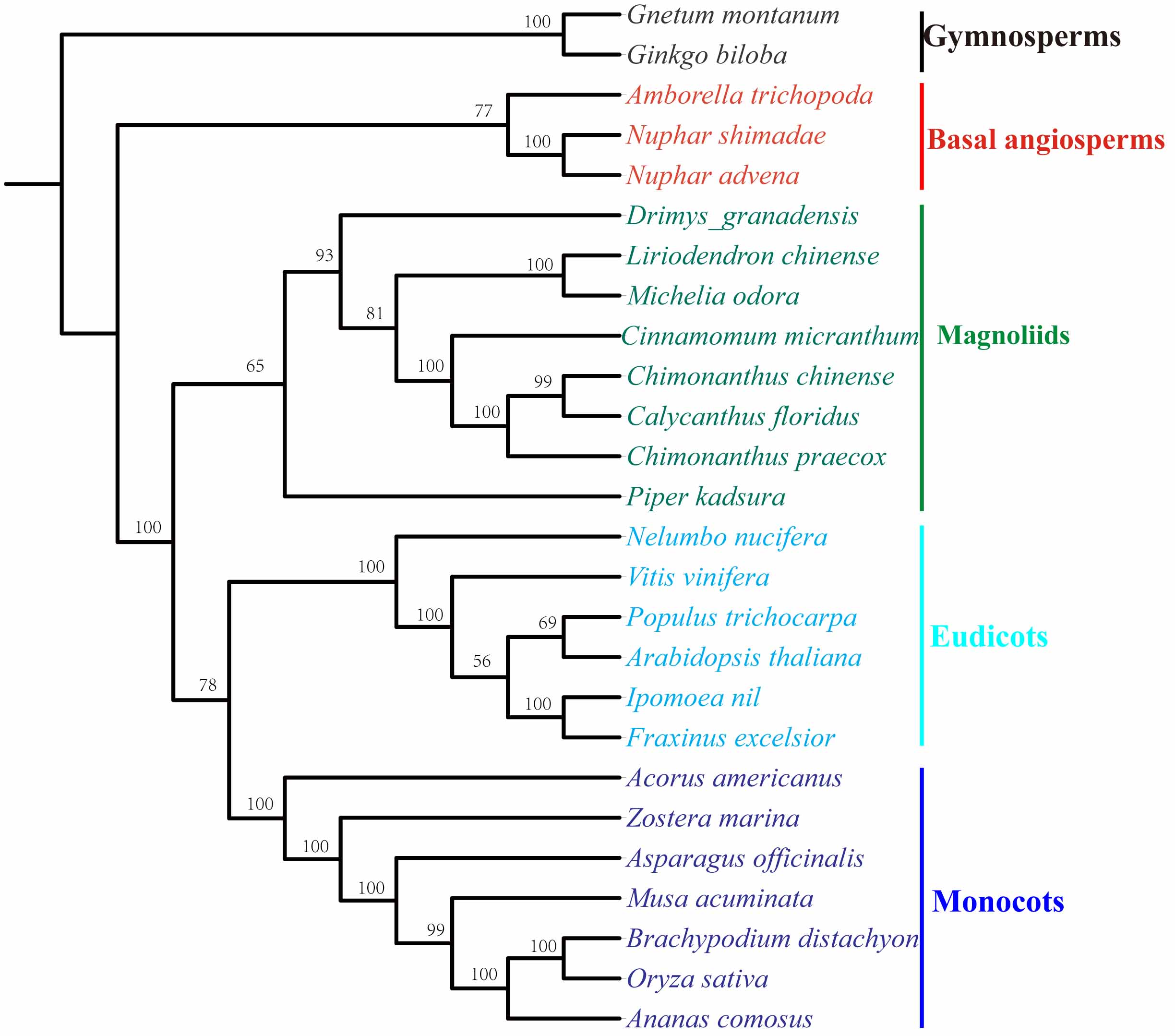


**Fig. S9: The phylogenetic tree based on 38 chloroplast genes from 26 species.** The phylogenetic tree was constructed from 38 single copy chloroplast gene sequences that were shared among 26 plant species using the ML method. Numbers associated with nodes are bootstrap values.


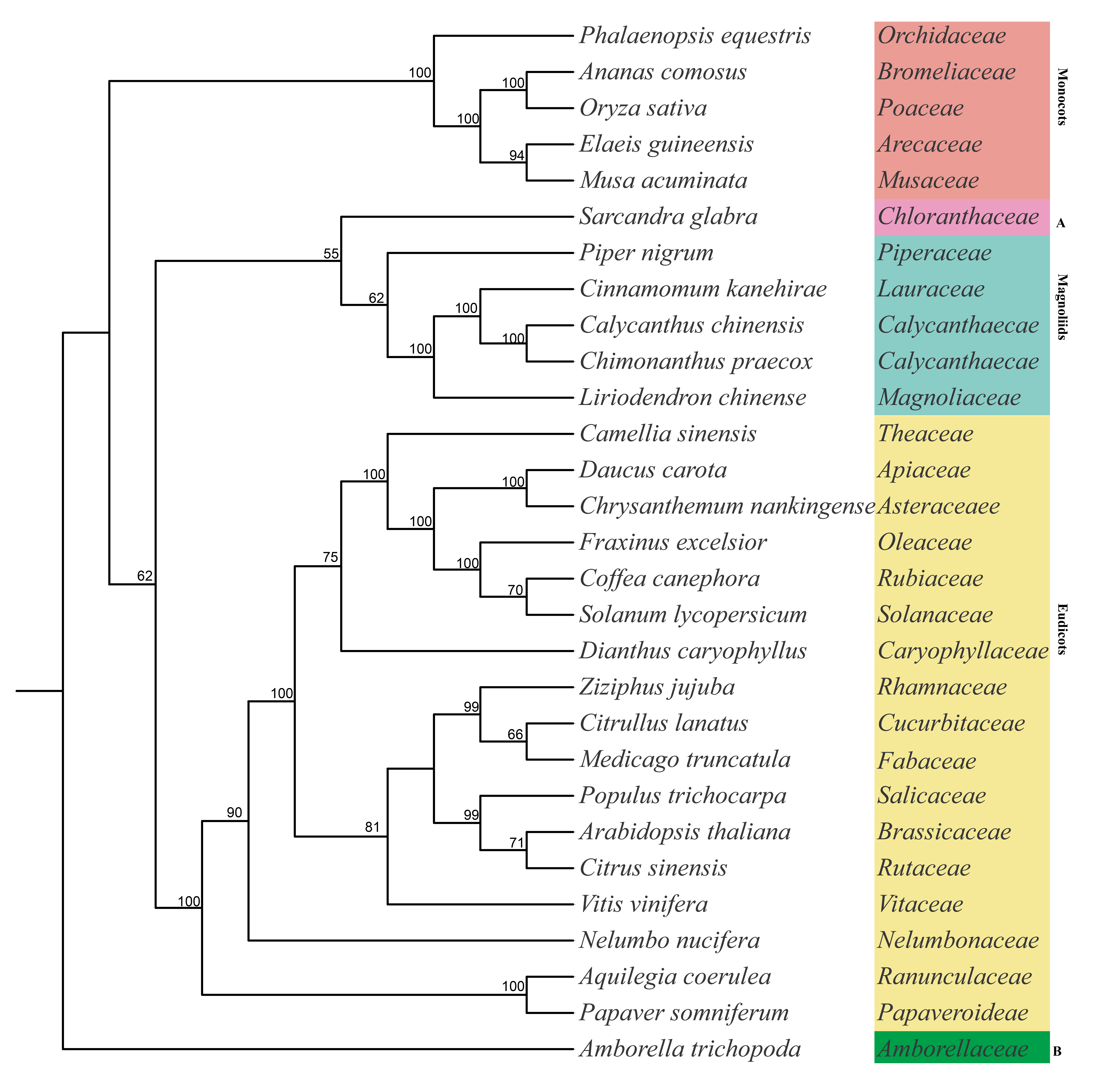


**Fig. S10: A phylogenetic tree of 29 plant species based on 2420 concatenated genes trees using RAxML.** Different colors on the right side represent different clades. A and B represent Chloranthaceae and basal angiosperm respectively.


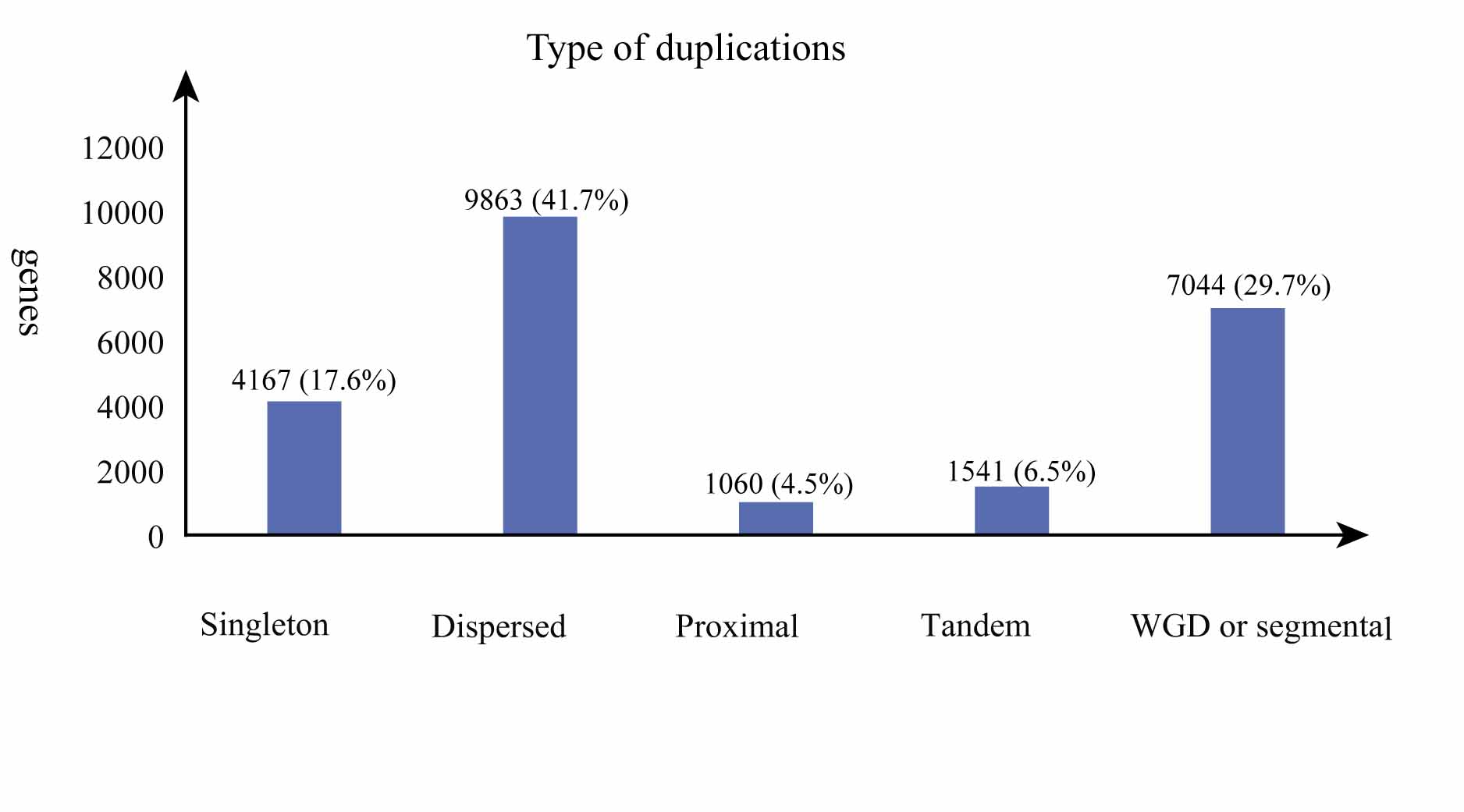
**Fig. S11: Types of gene duplication in the wintersweet genome.** Distribution of the five duplication types classified by MCScanX as follows: Singleton: no duplication; WGD/segmental: whole genome or segmental duplications (collinear genes in collinear blocks); Tandem: consecutive duplication; Proximal: duplications in nearby chromosomal region but not adjacent; Dispersed: duplications of modes other than tandem, proximal or WGD/segmental.


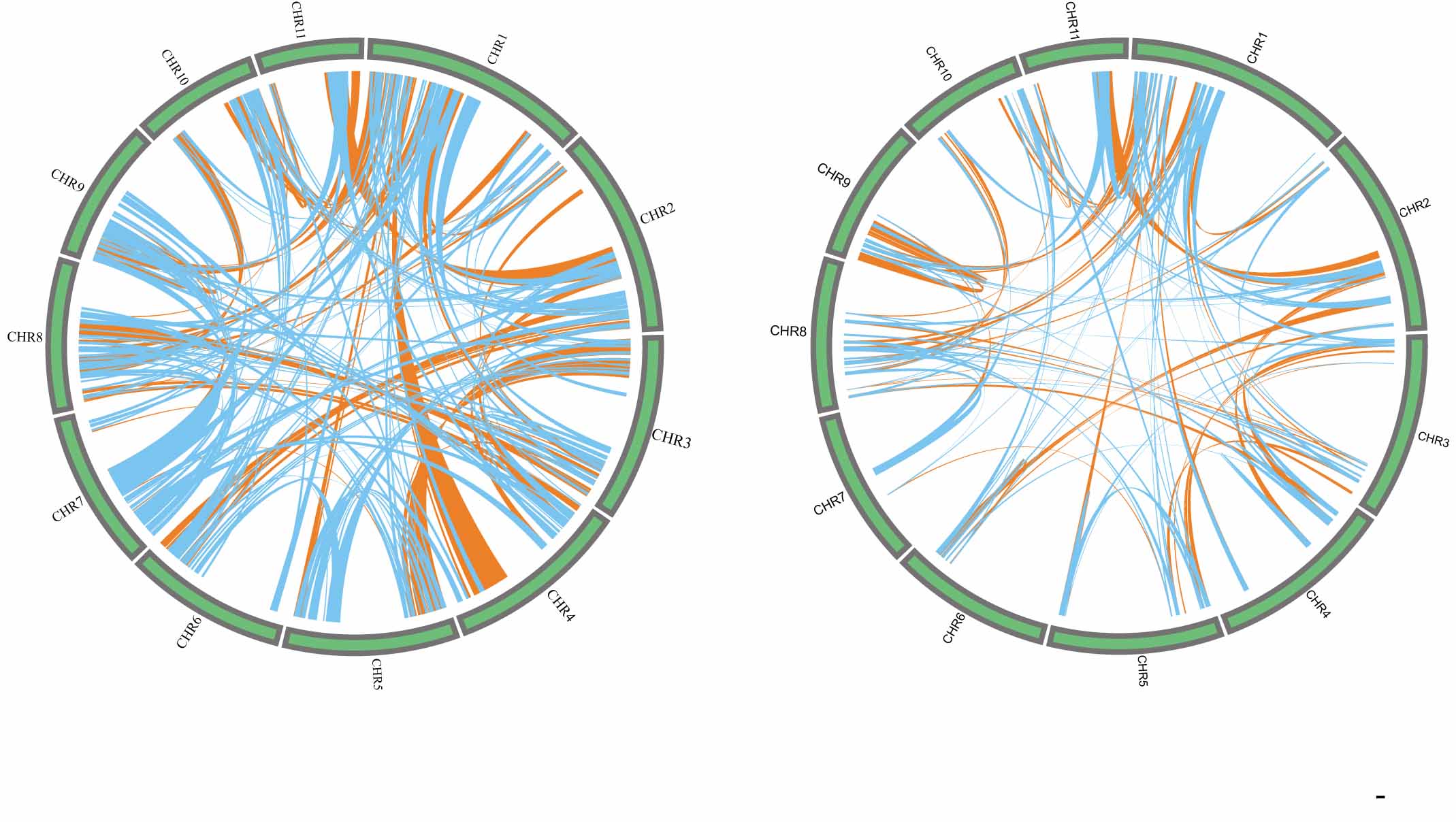
**Fig. S12: Duplications of genomic paralogous genes in wintersweet.** (a) The syntenic blocks corresponding to the ancient WGD (blue) and recent WGD (orange). (b) The overlap synthetic blocks between the ancient WGD (blue) and recent WGD (orange).


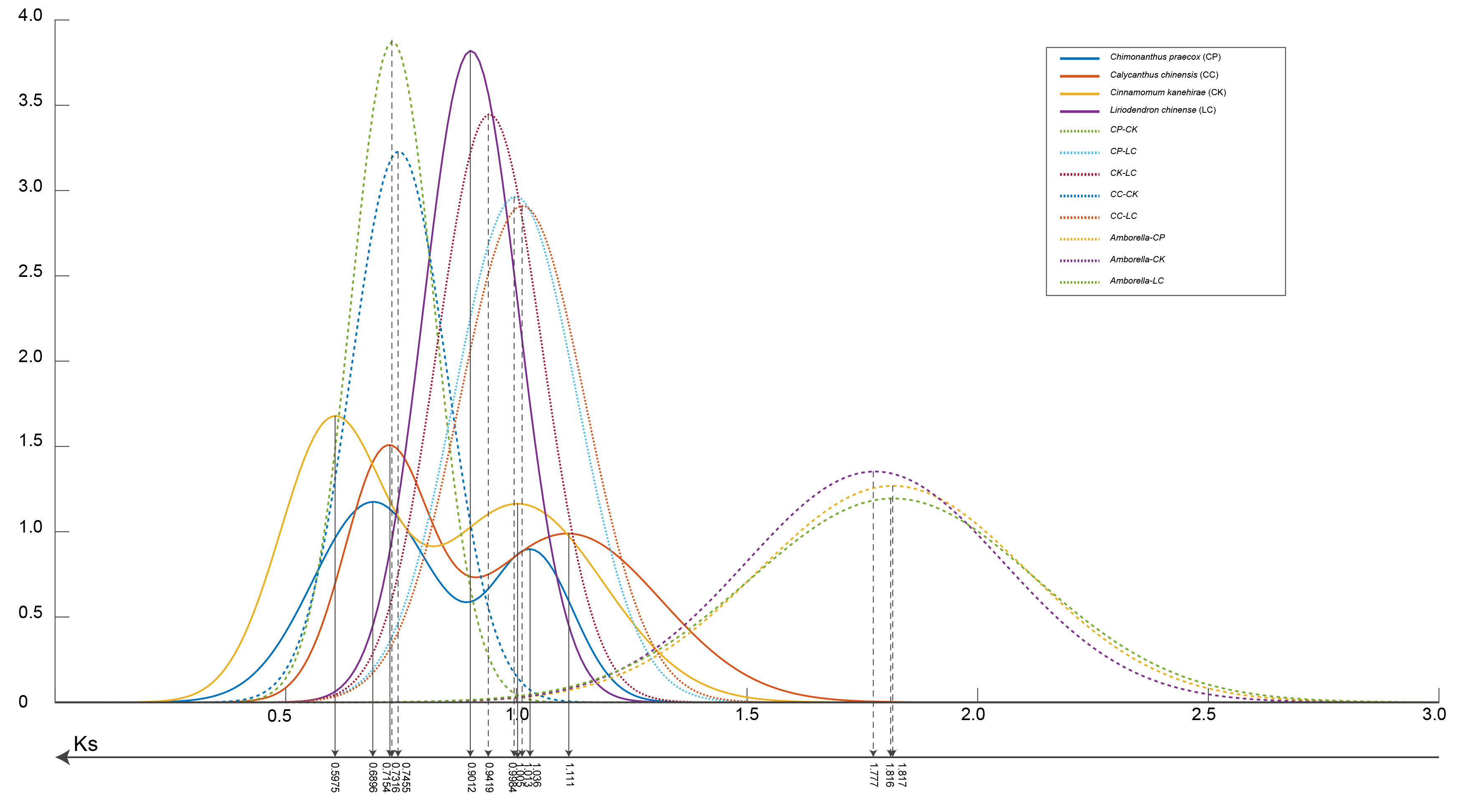


**Fig. S13 Distribution of synonymous substitution levels (Ks) of syntenic orthologous (solid curves) and paralogous genes (dashed curves).**

**
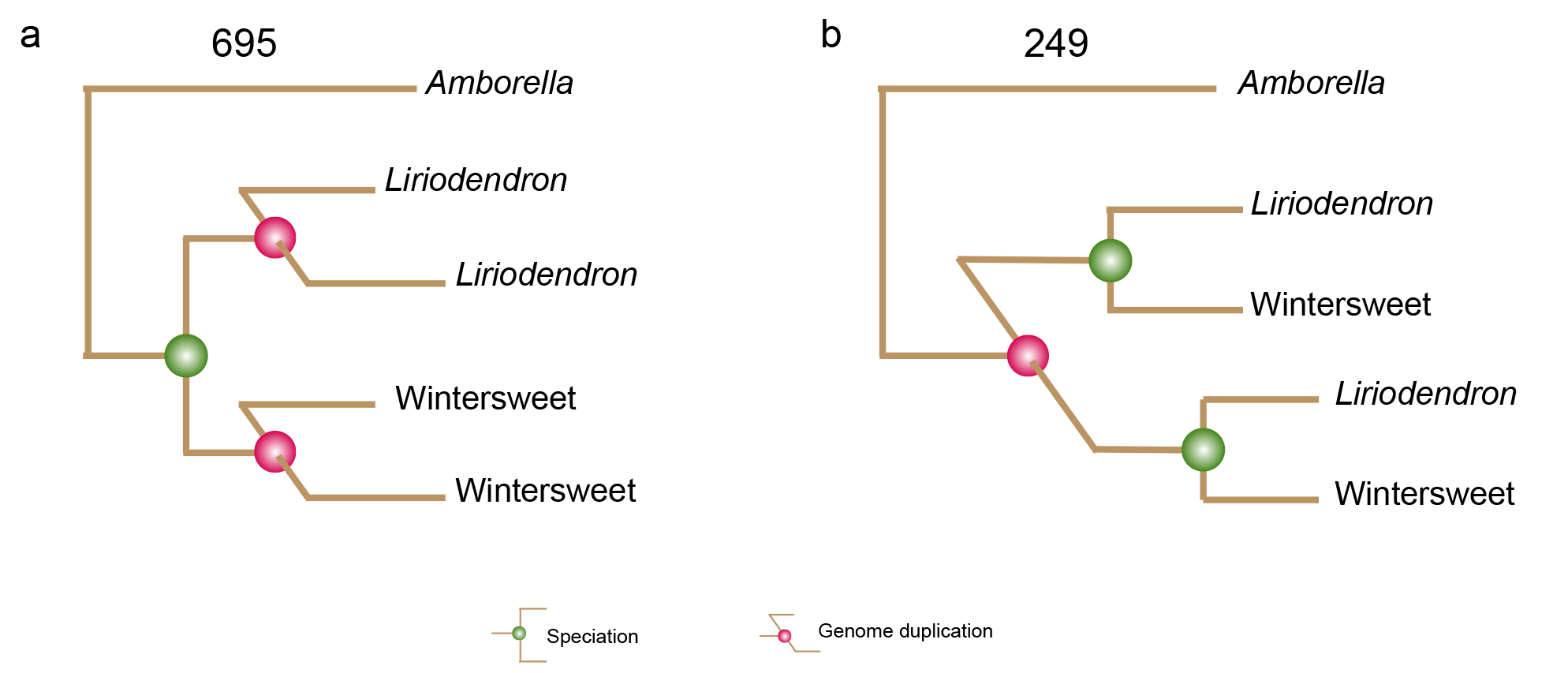
Fig. S14: Topologies of gene trees depicting the two possible scenarios of speciation among wintersweet and *Liriodendron.*** (a) One possible speciation scenario, in which WGD happened before the speciation between wintersweet and *Liriodendron*. (b) The alternative speciation hypothesis in which WGD happened independently after the speciation between wintersweet and *Liriodendron*. The numbers shown on top of the trees represent the number of gene clades supporting different hypotheses on the order of speciation and whole-genome triplication event in the wintersweet and *Liriodendron*.

**
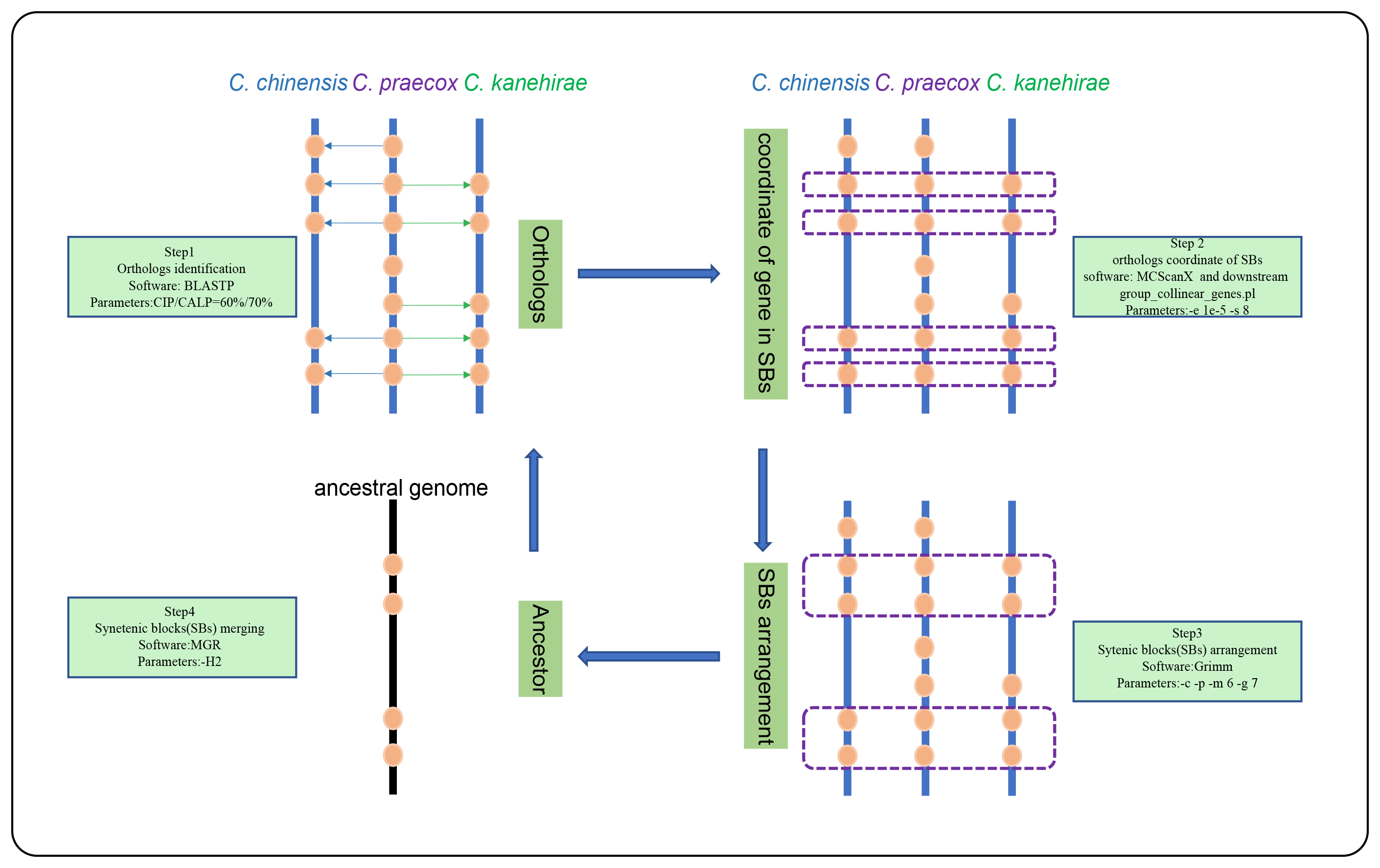
**

**Fig. S15: A modified pipeline of ancestral genome reconstruction (Murat et al.,** 2017 **Nature Genetics).** Analysis pipeline indicating the steps of ancestral genome reconstruction. The ancestral karyotype of *C. praecox*, *C. chinensis* and *C. kanehirae*, was determined by genome alignments using cumulative identity percentage (CIP) and cumulative alignment length percentage (CALP) BLAST parameters. The conserved genes among the three genomes were extracted from these alignments and then used to identify syntenic blocks by MCScanX. Then the syntenic blocks were merged using GRIMM to get the coordinate correspondence between the three collinear groups. Finally, the MGR software was used to rearrange the multiple genomes to get the ancestral gene orders.


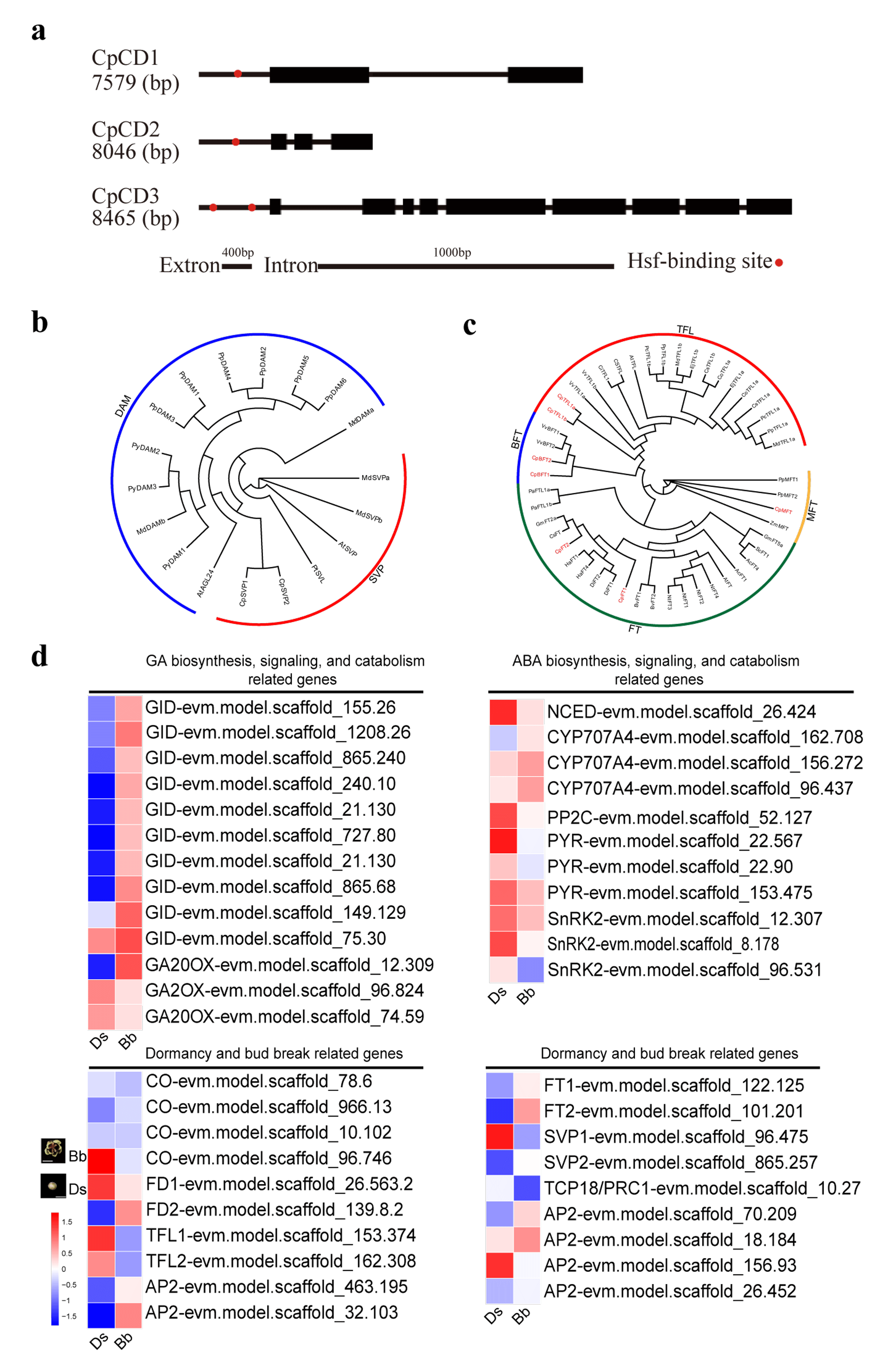


**Fig. S16: Phylogenetic analysis of FT-like and SVP-like and the analysis of bud break and cell division (*CpCDs*) related genes and genes involved in phytohormone-related pathways.** (a) This tree was derived from 7 FT/TFL1-like genes from wintersweet genome and 38 FT/TFL1-like genes from other15 species. *Ac*, *Allium cepa*; *Cs*, *Chaenomeles sinensis*; *Ci*, *Citrus* *sinensis*; *Co*, *Cydonia oblonga*; *Di*, *Dimocarpus* *longan*; *Gm*, *Glycine max*; *Ha*, *Helianthus annuus*; *Nt*, *Nicotiana tabacum*; *Bv*, *Beta vulgaris*; *At*, *Arabidopsis thaliana*; Sc, *Saccharum hybrid cultivar*; *Pp*, *Pyrus pyrifolia*; *Pc*, *Pyrus communis*; *Md*, *Malus* *domestica*; *Ej*, *Eriobotrya japonica*;*Vv*, *Vitis vinifera*; *Zm*, *Zea mays*. The different color curve represent four subclades respectively. (b) Phylogenetic tree of Wintersweet SVPs with Arabidopsis *SVP* and *AGL24* (AT2G22540, AT4G24540), DAM genes from peach (*PpDAM1-6*), DAM genes from pear (*PmDAM6*) and DAM and SVP-Like genes from Apple (*MdDAMa* and *MdDAMb*, *MdSVPa* and *MdSVPb*). (c) Expression heatmap of bud break-related genes and genes involved in phytohormone-related pathways. The color scale for transformed FPKM value by log2 was shown on the left. Bb: Bud break stage; Ds: Dormancy stage. (b) Structures of *CpCDs* in wintersweet. Boxes and lines represent exons and introns, whereas red points represent Hsf-binding site.


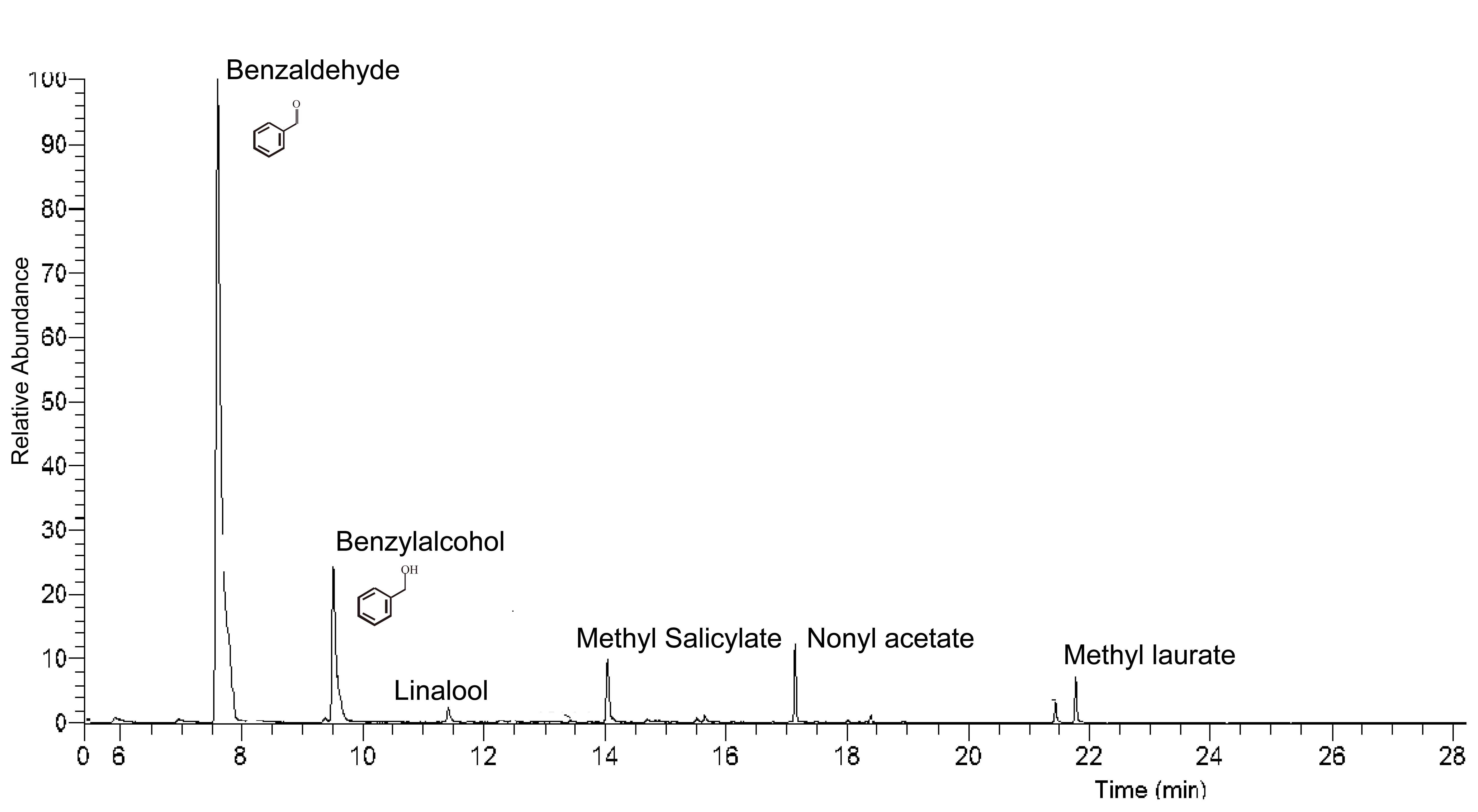


**Fig. S17: Gas chromatogram of glycosidic floral volatiles from the flowers of wintersweet.** The internal standard (IS) is nonyl acetate. Glycosidic floral volatiles was measured three times independently with similar results.


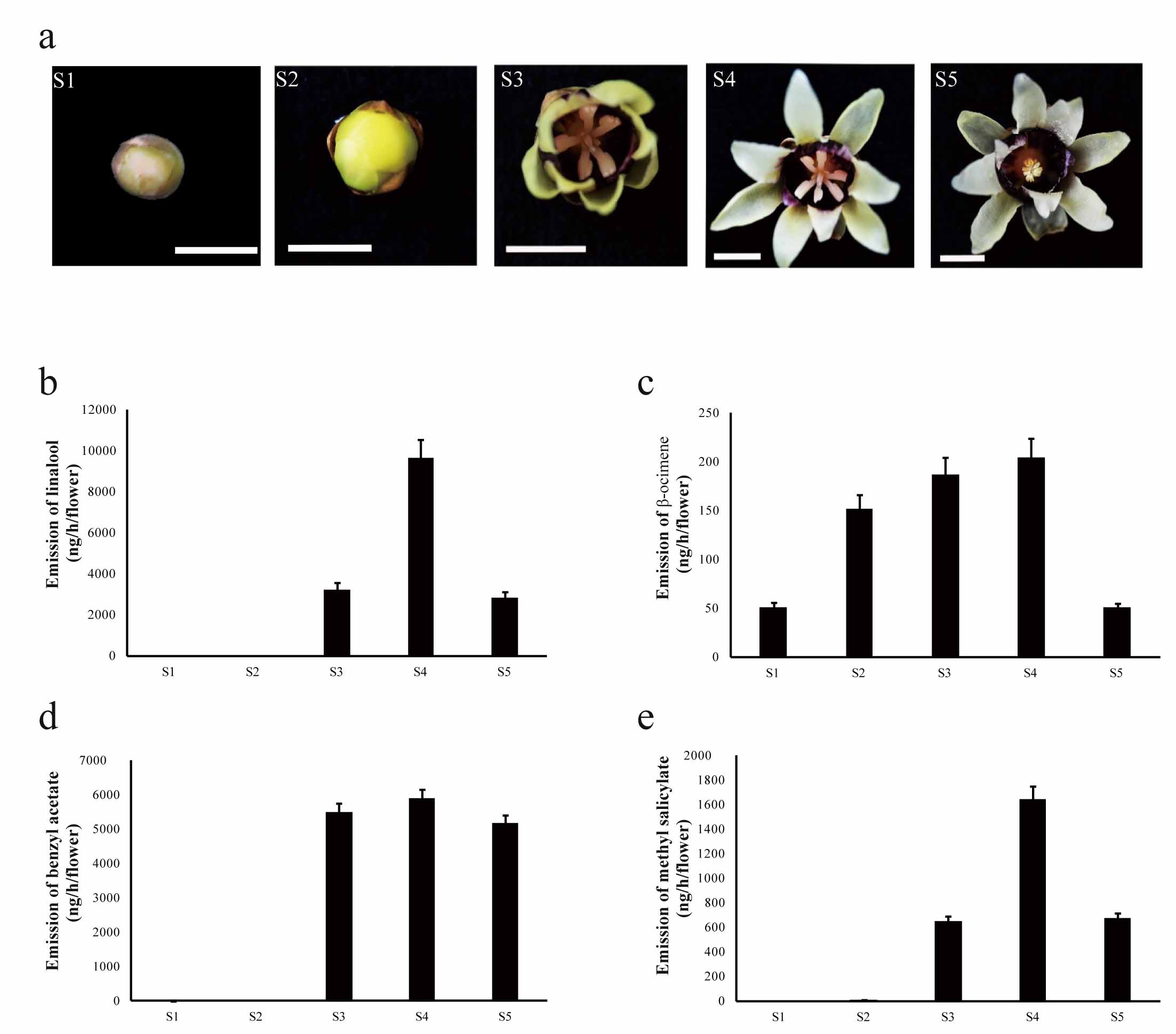


**Fig. S18: Changes of the major floral volatiles during flower development in wintersweet.** (a) flower phenotypes at different stages. (b-e) Emission of linalool (b), β-ocimene (c), benzyl acetate (d), methyl salicylate (e), in flowers at five developmental stages. Date represent mean s ± SD (n=3) from three biological replicates of every flower stage.


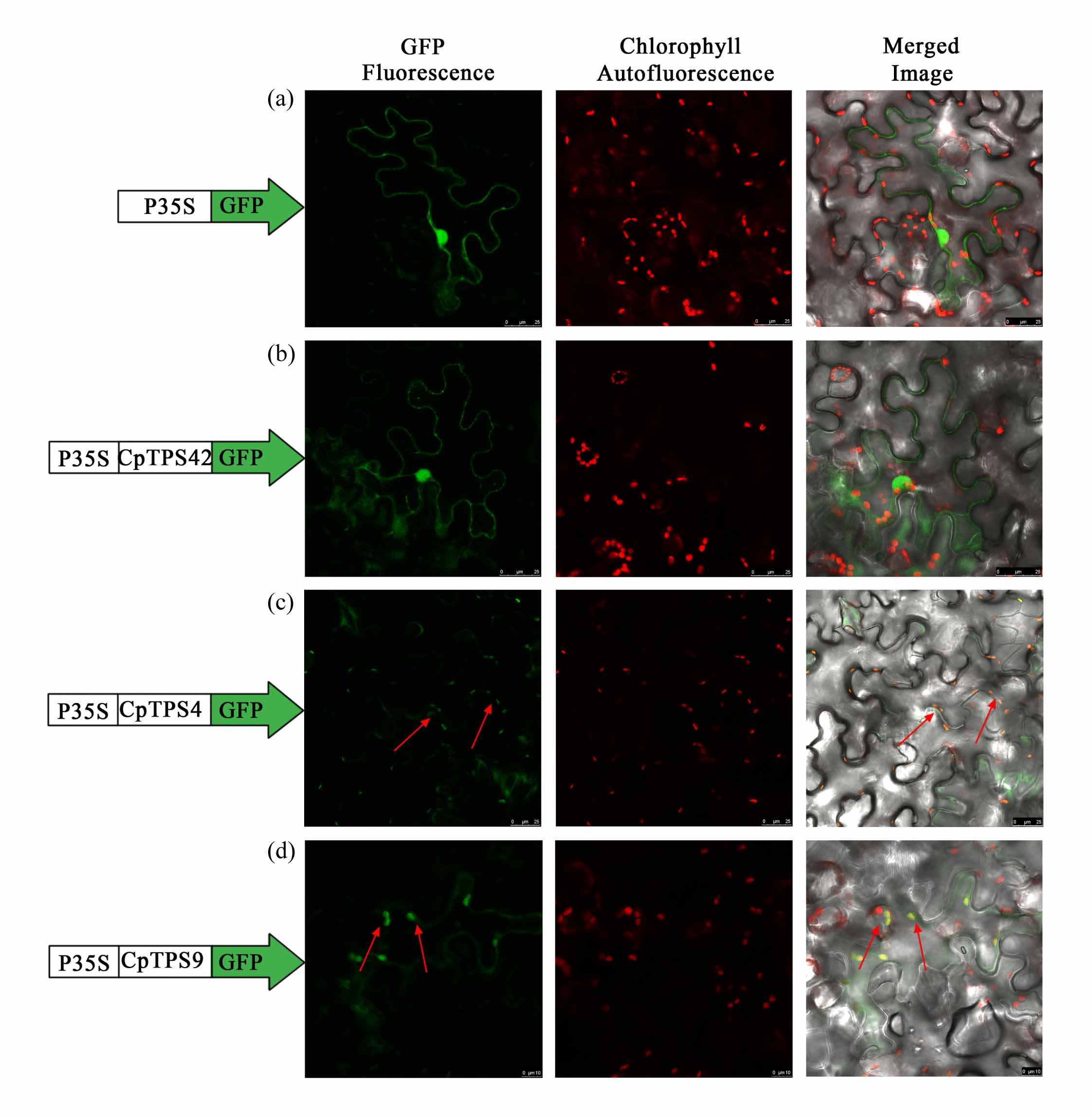
**Fig. S19: Subcellular location of CpTPS proteins.** (a) p35S-GFP control in leaf abaxial surface. (b) Subcellular location of CpTPS42 protein fused with green fluorescent protein (GFP) transiently expressed in *Nicotiana benthamiana* leaves. (c) Subcellular location of CpTPS4 protein fused with green fluorescent protein (GFP) transiently expressed in *N. benthamiana* leaves. (d) Subcellular location of CpTPS9 protein fused with green fluorescent protein (GFP) transiently expressed in *N. benthamiana* leaves. GFP alone and chlorophyll autofluorescence were used as cytosolic and plastidic markers respectively. Arrow indicate chloroplasts. Bars= 5 μm.


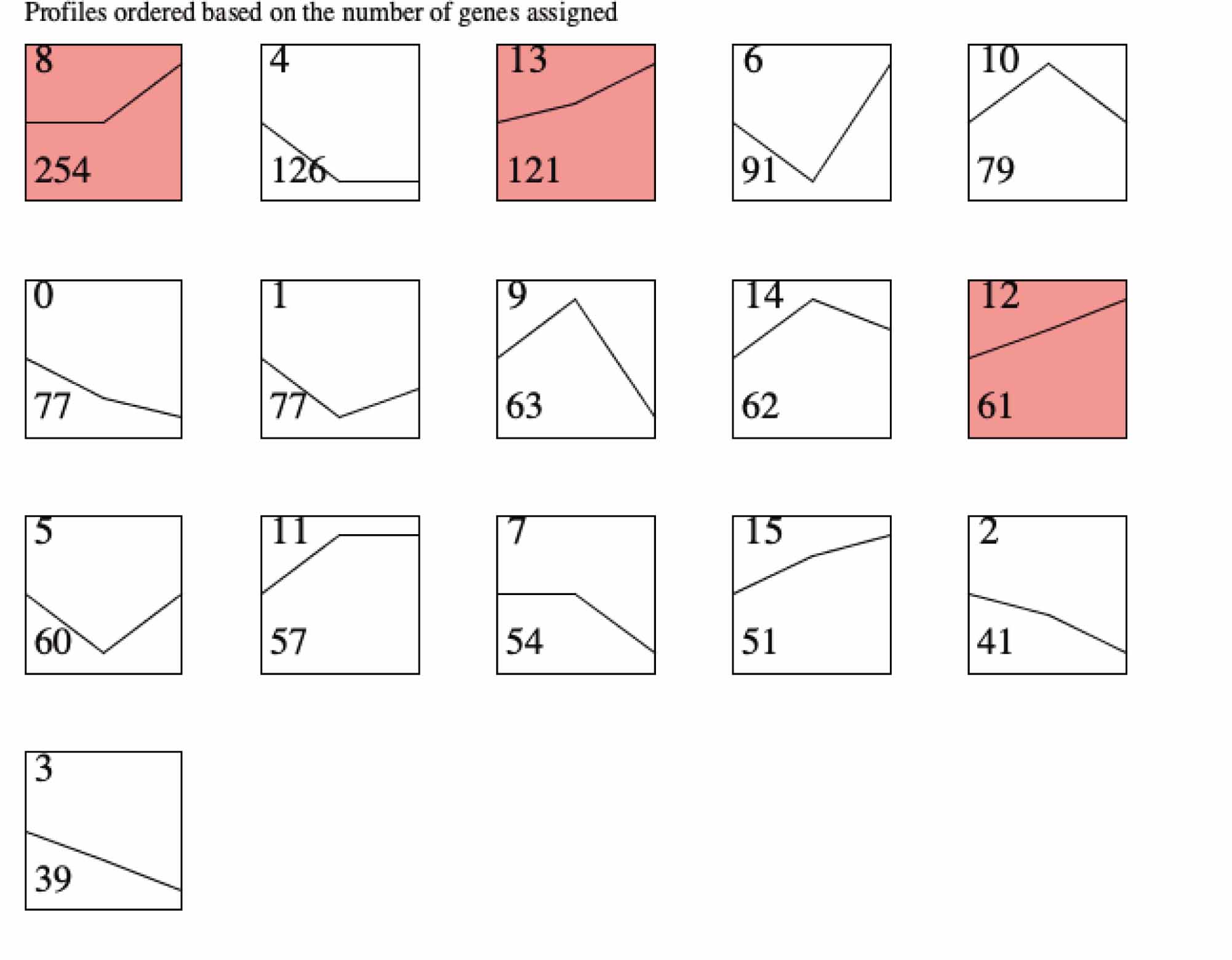


**Fig. S20: Cluster analysis of differentially expressed TFs in three floral developmental stages (S1, S4, S5).**  All differentially expressed TFs were divided into fifteen distinct temporal expression profiles using STEM software. Clusters in color indicate the expression level of TFs is significantly different between different stages (p value ≤ 0.05). The number on the top is a cluster number. The number at the bottom is gene number assigned in each cluster. S1: bud stage; S4: full open flower stage; S5: senescence stage)**.** The list of the genes belonging to each cluster were shown in Supplementary Table 17.


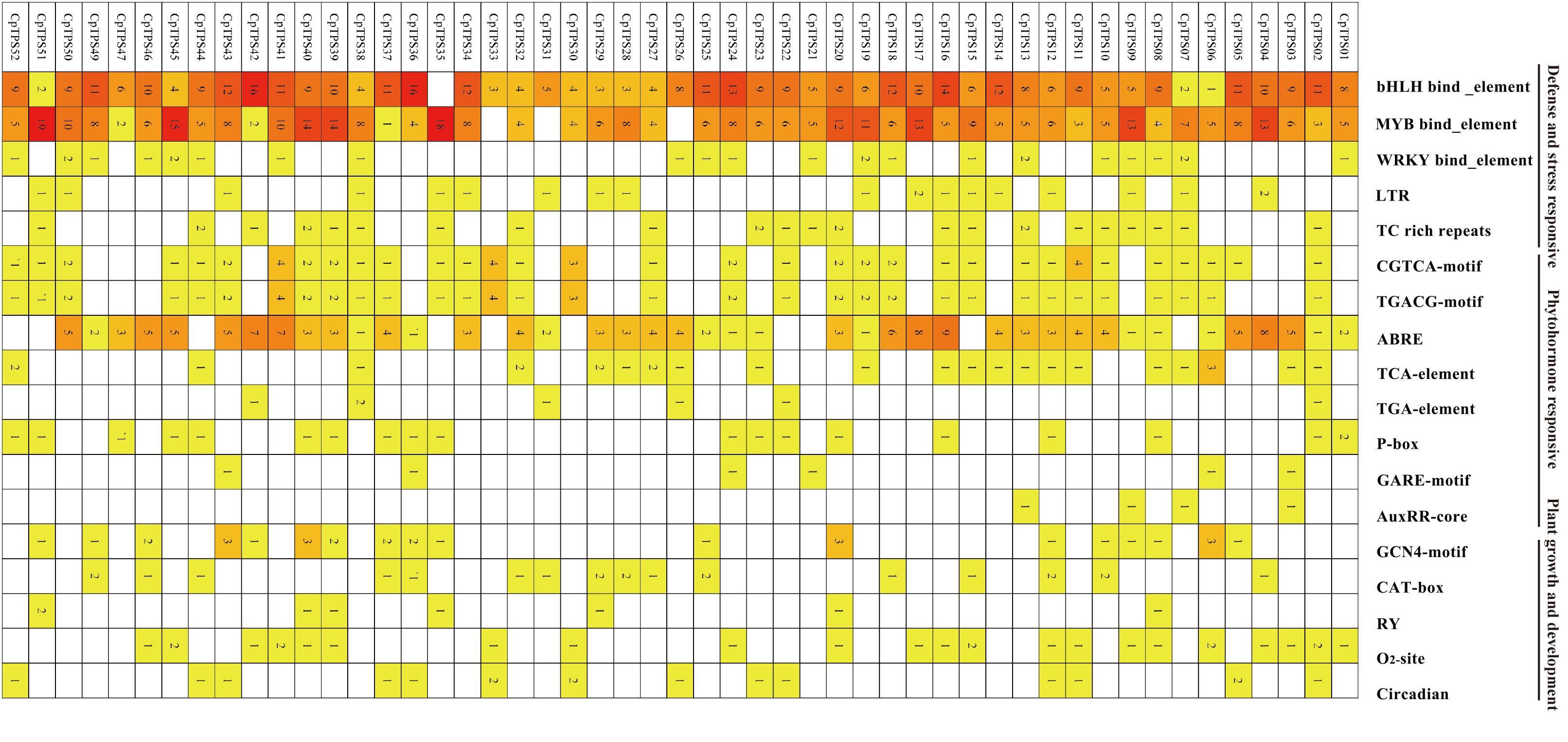


**Fig. S21: Promoter cis-element analysis of 52 *CpTPSs*.** The cis-element types are shown on the right site. The number of each type for each gene was marked.


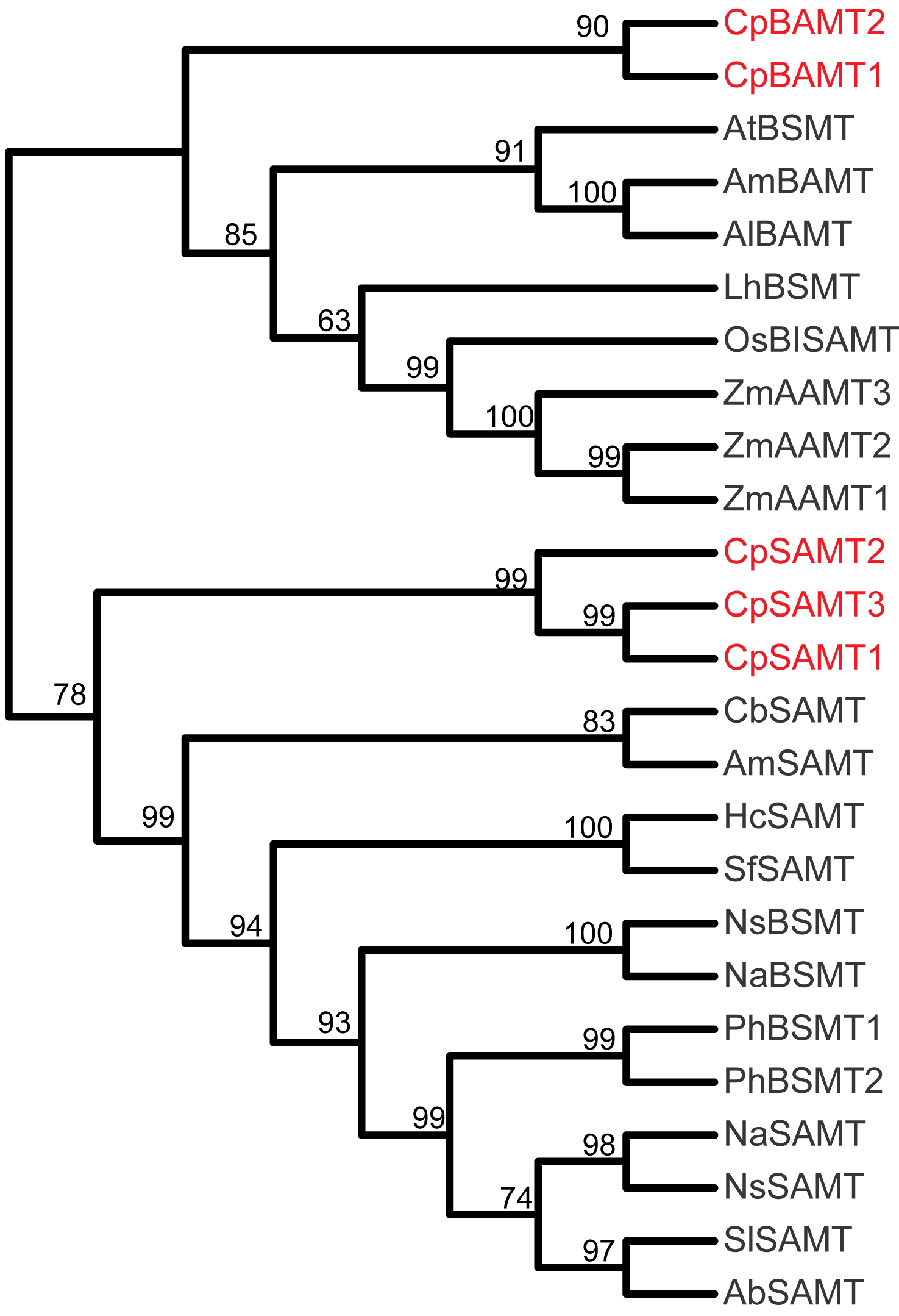


**Fig. S22: Analysis of benzenoid carboxyl methyltransferases (*BCMTs*).** (a) Phylogenetic analysis of benzenoid carboxyl methyltransferases (*BCMTs*) in wintersweet. The phylogenetic tree of *CpBCMTs* with other functional BCMTs using neighbor-joining method. Proteins identified from wintersweet are marked in red. *Ab*, *Atropa belladonna*; *At*, *Arabidopsis thaliana*; *Al*, *Antirrhinum linkianum*; *Am*, *Antirrhinum majus*; *Cb*, *Clarkia breweri*; *Hc*, *Hoya carnosa*; *Lh*, *Lilium hybrid*; *Na, Nicotiana alata*; *Ns*, *Nicotiana suaveolens*; *Os*, *Oryza sativa*; *Ph*, *Petunia hybrid*; *Sf*, *Stephanotis floribunda*; *Sl*, *Solanum lycopersium*; *Zm*, *Zea mays*.

**
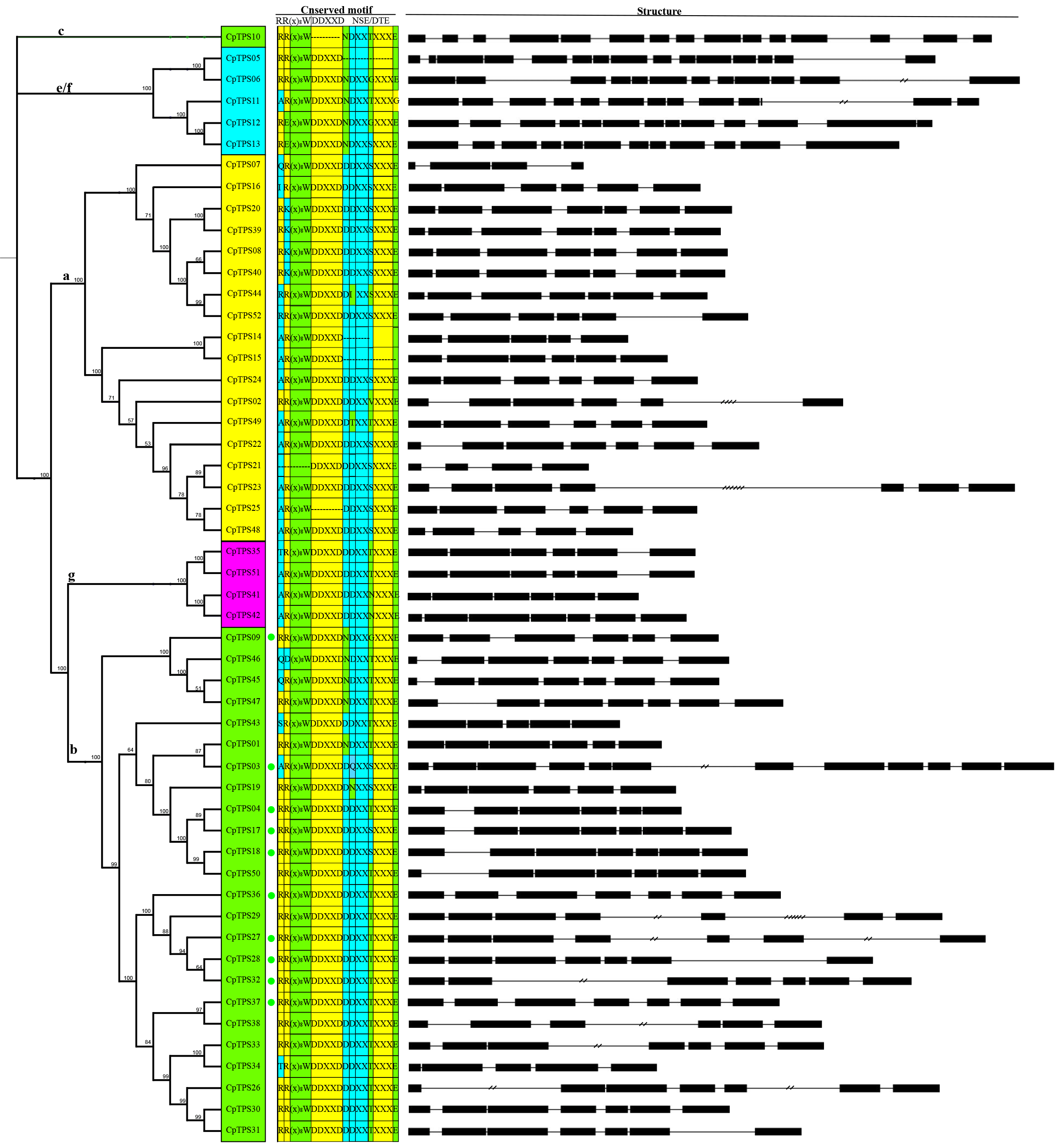
**

**Fig. S23: Gene structure and classification of putative *CpTPSs***. Exon-intron structure were predicted and curated for the putative intact CpTPS gene models. Black squares and black line represent at scale protein coding exons and introns respectively. The black line with two slash indicated the lenth of intron is half of its real scale and black line with four slash indicated the lenth of intron is two times shorten than its real scale to make the image more regular. Green circles indicate the prediction of an N-terminal plastidial targeting peptide. The conserved motifs RR(x)_8_W, DDxxD, NSE/DTE are represented by green, yellow and cyan boxes respectively. Genes are clusted into subfamilies: a,b,c,e/f,g, based on pylogenetic analyses using amino acid sequences.


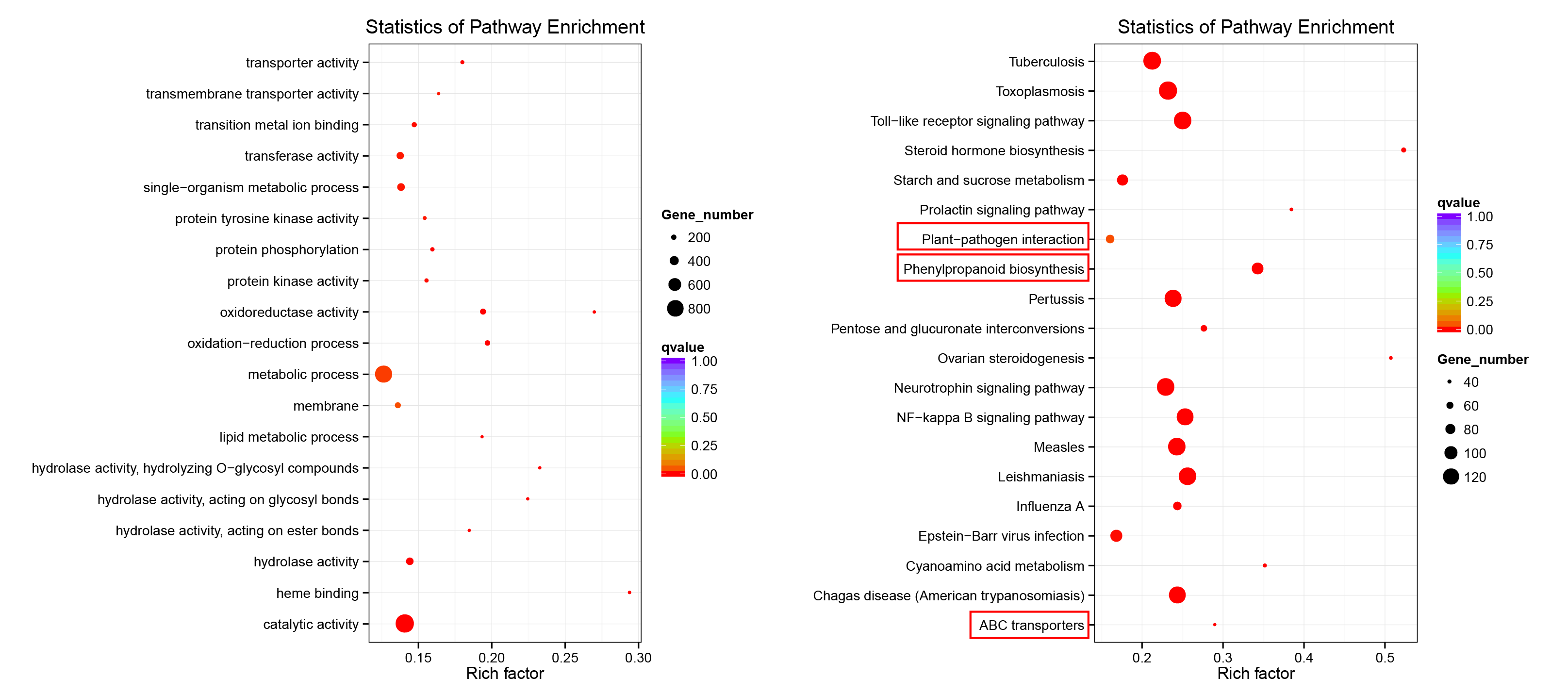


**Fig. S24:** **Annotation of the Tandem duplicated genes.** (a) KEGG pathway enrichment distribution of the tandem duplicated genes. (b) GO functional enrichment of the tandem duplicated genes.
