# Supplementary material for: The chromosome-level wintersweet (Chimonanthus praecox) genome provides insights into floral scent biosynthesis and flowering in winter
Source: Genome Biol. 2020 Aug 10;21:200. doi: 10.1186/s13059-020-02088-y (PMC7419205; doi:10.1186/s13059-020-02088-y)
Supplement: Supplementary file 3 — Additional file 3: Supplementary Note 1. Estimation of Genome Size and Chromosome Number Assessment; Identification of Orthologs of Flowering Time Genes; Identification of MADS-box Genes; Genes Expression Analysis. Supplementary Note 2. Headspace Collection and GC–MS Analyses of Floral Volatiles; Changes of volatile compounds during flower development; Phylogenetic and Structural Analysis of TPS Family; Subcellular Location of the fused CpTPS4/9/42-fused green Fluorescent Protein; Construction of CpTPS4 Overexpressed Tobacco Plants, Analysis of Transcription of Factors; Analysis of the genes involved in terpene and benzenoid biosynthesis transport. Supplementary Note 3. Tandem duplication analysis; The order of speciation and WGD event in Liriodendron and wintersweet lineage. [file 13059_2020_2088_MOESM3_ESM.doc]

**The chromosome-level wintersweet (*Chimonanthus praecox*) genome provides insights into floral scent biosynthesis and flowering in winter**

**Authors:**

Junzhong Shang^1,#^, Jingpu Tian^3, #^, Huihui Cheng^6,#^, Qiaomu Yan^1^, Lai Li^1^, Abbas Jamal^1^, Zhongping Xu^4,5^, Lin Xiang^1^, Christopher A Saski^7^, Shuangxia Jin^4,5^*, Kaige Zhao^1^*, Xiuqun Liu^1^*, Longqing Chen ^2^*

**Authors affiliations:**

^1^Key Laboratory of Horticultural Plant Biology, Ministry of Education, Huazhong Agricultural University, Wuhan, Hubei, 430070, P. R. China

^2^Southwest Engineering Technology and Research Center of Landscape Architecture, State Forestry Administration, Southwest Forestry University, Kunming, Yunnan, 650224, P. R. China

^3^School of Architecture and Art Design，Hunan University of Science and Technology，Xiangtan, Hunan, 411201, P. R. China

^4^National Key Laboratory of Crop Genetic Improvement, Huazhong Agricultural University, Wuhan, Hubei, 430070, P. R. China

^5^Xinjiang Production and Construction Corps Key Laboratory of Protection and Utilization of Biological Resources in Tarim Basin, Tarim University, Alaer, Xinjiang 843300, China

^6^Novogene Bioinformatics Institute, Beijing, 100083, P. R. China

^7^Department of Plant & Environmental Science, Clemson University, SC 29631 USA

**Supplementary Note 1**

**Estimation of Genome Size and Chromosome Number Assessment**

We estimated the genome size of *Chimonanthus praecox* and *Calycanthus chinensis* by using flow cytometry. In brief, the fresh young leaves of *C. praecox* were chopped up with a sharp blade and then soaked in WPB lysis buffer (200 mM Tris-HCl, 4 mM MgCl_2_·6H_2_O, 86 mM NaCl, 10 mM sodium metabisulfite, 1% [v/v] Triton X-100 [pH 7.5]) for 3 min. Samples were then filtered through 25-μm nylon net and the filtrate was transferred into 2ml centrifuge tube. The plant cell nuclei in the tube were stained by adding 50 μl DAPI buffer (50μg/ml) and RNAse in the dark for 10 min. The relative genome size was tested by flow cytometer (BD FACSAria IV) using *Oryza sativa* ‘Nipponbare’ as an external standard. Using this method, we identify the genome size of *C. praecox* and *C. chinensis* as 805.88 Mb and 838.38 Mb respectively. In addition, we further evaluated the genome size by analyzing the K-mer frequency [1]. We selected high-quality pair-end reads from *C. praecox* and *C. chinensis* and generated 17-mer frequency information based on the k-mer analysis. This analysis gave us an estimated genome size of 778.71 Mb (*C. praecox*) and 827.03 Mb (*C. chinensis*), respectively. Fresh young leaves from current year-old branches were used to examine the chromosome number according to the protocol described by Suen et al. (1997) [2]. The stained samples were observed under microscope (BX61, Olympus, Tokyo, Japan) (Additional file 2: Figure S3).

**Identification of Orthologs of Flowering Time Genes**

We used the database of flowering time genes in *Arabidopsis thaliana*, FLOR-ID [3], to identify the corresponding orthologs in *C. praecox*. This database consist of 295 protein-coding genes, the protein sequences of which were firstly input into the PfamScan (https://www.ebi.ac.uk/Tools/pfa/pfamscan/) to identify their functional domains and classifications with an E-value cutoff of 10^–5^. The flowering-time genes were then divided into domain-containing genes and non-domain-containing genes. The Hidden Markov Model (HMM) profiles of functional domain-containing genes were downloaded from the HMMER web server (<https://www.ebi.ac.uk/Tools/hmmer/>) and used as queries search against the proteome of *C. praecox* using the HMMER [4] to determine the candidate genes containing corresponding functional domains with a filtered parameter (E-value < 10^-5^, identity ≥ 50%, and coverage ≥ 30%). For larger-size gene family, the genes were determined by combining local blast reports and phylogenetic analysis to distinguish the orthologs of corresponding genes. The phylogenetic tree was constructed with fastTree (<http://www.microbesonline.org/fasttree/>) using the amino acid sequences alignments generated by MUSCLE [5]. For the flowering-time genes without functional domains in *A. thaliana*, their protein sequences were used as queries in a reciprocal BLASTP analysis to search against the wintersweet proteome database, at the threshold and minimum alignment coverage parameters as described above.

**Identification of MADS-Box Genes**

MADS-Box family genes from Arabidopsis and rice were downloaded from TAIR website (<http://www.arabidopsis.org/>) and the Rice Genome Annotation Project (<http://rice.plantbiology.msu.edu/>) respectively. The amino acids of these genes were used as query sequences by blastp searches (E-value < 10^-5^) against the proteome of *C. praecox* to predict the MADS-Box family genes in *C. praecox*. The resulting sequences were screened by the Hidden Markov Model (HMM) profile for the MADS-box domain (Pfam domain: PF00319) using HMM search tool to ensure the completeness of MADS-box domain. These two methods mentioned above were intersected to guarantee the appropriate selection of genes. The protein sequences of the selected genes and MADS-box genes from Arabidopsis and rice were aligned using ClustalX software package with default parameters. A phylogenetic tree was constructed with aligned MADS-box protein sequences by using the neighbour-joining (NJ) method with 1000 iterations for the bootstrap values.

**Genes Expression Analysis**

One microgram of total RNA was reverse transcribed into cDNA using the TransScript II One-Step gDNA Removal and cDNA Synthesis SuperMix (TransGen Biotech, BeiJing China), according to the manufacturer’s protocol. Ten folds diluted cDNA was used as the template for subsequent qRT-PCR analysis using the SYBR® Premix Ex Taq™ II mix (Takara Biotechnology Co., Dalian, China) on Applied Biosystems 7500 Fast Real-Time PCR platform, with primer sequences listed in (Additional file 1: Table S22). Three biological replicates were tested, and reactions were carried out in triplicate. Relative gene expression was normalized against the expression of the -actin gene and analyzed using the 2^–ΔΔCT^ method [6].

**Supplementary Note 2**

**Headspace Collection and GC–MS Analyses of Floral Volatiles**

To analyze the floral scent composition of wintersweet flowers, the volatile compounds were collected from fresh flowers of five stages (Additional file 2: Figure S18a) using a headspace solid-phase microextraction (HS-SPME) device as described previously [7]. The identification of the VOCs was based on the comparison of the obtained mass spectra with the reference library NIST MS 2008 using RI value. Some compounds including linalool, ocimene, benzyl alcohol, benzyl acetate and methyl salicylate (Sigma-Aldrich) were confirmed by using the Kovats retention index or GC retention data with those of authentic standards. Quantification was based on peak areas and the quantity of the internal standard using the Thermo Scientific Xcalibur Data Analysis Application.

**Changes of volatile compounds during flower development**

The linalool, methyl salicylate and β-ocimene compounds were hardly detectable at bud stage, the emission of which reached up to the maximum at full open flower stage and declined thereafter (Additional file 2: Figure S18b-c,e). The emission levels of benzyl acetate compounds were low at bud stage and increase significantly when anthesis, while the emissions of which remain high in the process of senescence (Additional file 2: Figure S18d). These results indicated that all floral scents mentioned above were controlled

**Phylogenetic and Structural Analysis of TPS Family**

Phylogenetic analysis of TPS from four species revealed that all CpTPS genes were clustered into five of seven subfamilies described for land plants (Fig.7a). The majority of CpTPS genes were placed in the TPS-a (18) and TPS-b (24) subfamilies. Protein subcellular location prediction of the CpTPS genes indicated that ten genes in TPS-b subfamily have plastid transit peptides which is essential for plastid location of monoterpene synthases. In the TPS-g subfamily, there are four members which are probably to encode acylic monoterpene synthases. The CpTPS genes belonging to TPS-c (1) and TPS-e/f (5) subfamilies probably encode diterpene synthases, such as monofunctional copal diphosphate synthase (CPS) and kaurene synthase (KS). These genes enzymes are supposed to be eudicot specific and responsible for the formation of diterpenoids. The intron number of CpTPS genes in TPS-c and TPS-e/f subfamilies vary from 12 to 15, which is more than that in TPS-a, TPS-b and TPS-g ranging from 5 to 6. The average amino acids length of CpTPSs (782aa) in TPS-c and TPS-e/f subfamilies is longer than that (454aa) in TPS-a, TPS-b and TPS-g (Additional file 2: Table S19). These results suggested that CpTPS genes in TPS-c and TPS-e/f subfamilies have a more complex gene structure compared with those in other subfamilies. The intron number of *CpTPSs* in TPS-c and TPS-e/f subfamilies vary from 12 to 15, which is more than that in TPS-a, TPS-b and TPS-g ranging from 5 to 6. The average amino acids length of CpTPSs (782aa) in TPS-c and TPS-e/f subfamilies is longer than that (454aa) in TPS-a, TPS-b and TPS-g (Additional file 2: Figure S23). These results suggested that CpTPS genes in TPS-c and TPS-e/f subfamilies have a more complex gene structure compared with those in other subfamilies.

**Subcellular Location of the fused *CpTPS4/9/42*-fused CsLIS/NES-green Fluorescent Protein**

The coding sequences of TPS genes (*CpTPS4*, *CpTPS9*, *CpTPS42*) were subcloned in-frame and upstream of the green fluorescent protein (GFP) in the cloning sites EcoRI and XbaI of p35S-GFP vector. The transient expression of CpTPS proteins in *Nicotiana benthamiana* was performed following the Sparkes method [8]. GFP fluorescent signal was observed 3 days after the infiltration using a Leica TCS SP8 confocal laser scanning microscope with excitation wavelengths of GFP and chlorophyll fluorescence at 488 and 543 nm, respectively. Triple dichroic filter was used to collect fluorescence (500-535 nm for green and 555-700 nm for red).

**Construction of *CpTPS4* Overexpressed Tobacco Plants**

Coding sequences of *CpTPS4* was subcloned into the BamH I and Sal I sites of pCAMBIA2300s vector under the control of the Cauliflower mosaic virus 35S promoter (Cambia, Canberra, Australia). The constructed vector was then introduced into *Agrobacterium tumefaciens* (EH105). *CpTPS4* overexpression transgenic plants as well as control plants carrying the empty pCAMBIA2300s vector were generated by standard Agrobacterium-mediated tobacco leaf disc transformation. Transgenic overexpression plants grown under the same conditions were then evaluated for plant volatile terpene content.

**Analysis of Transcription of Factors**

To gain the knowledge about the expression profiles of differentially expressed transcription factors (TFs) thorough three developmental stages (S1, S4 and S5), the short time-series expression miner (STEM) (http://www.cs.cmu.edu/~jernst/stem/) [9] was used to cluster differentially expressed TFs. Expression profiles were analyzed based on STEM clustering algorithm.

Orchestrated formation of the floral volatiles from several independent pathways not only depends on the functional biosynthetic enzymes but also requires the involvement of transcription factors. In previous studies, several TFs involved in the regulation of terpene biosynthesis have been isolated and characterized, while only two TFs regulating floral terpene biosynthesis have been studied, one *AtMYC2* in Arabidopsis [10] and one *PbbHLH4* in orchids [11]. Like in *Hedychium coronarium*, the 99 candidate TFs, whose expression was similar to the terpene emission profiles, were found to predominantly distribute in the class of bHLH and MYB TFs. These genes might play a critical role in regulating the formation of floral volatiles. The spatiotemporal expression patterns of function genes depend on variable cis-regulatory elements in their promoters. The expansion of bHLH and MYB binding site in the *CpTPSs* promoters further support the potential regulation of bHLH and MYB TFs on expression of *CpTPSs* genes. Further study on the involvement of TFs in floral scent formation using transient system such as virus silencing and over-expression, in combination with metabolite analysis, will be valuable and informative in the modifying floral scent traits of wintersweet in the future.

**Analysis of the genes involved in terpene and benzenoid biosynthesis transport**

Apart from volatile biosynthesis, their transportation between different subcellular compartments or cells is also the critical step for the final emission. The volatile transportation is biologically mediated by the transportors, such as plastidial cationic amino-acid transporter CAT [12], chloroplast envelope-located EDS5 [13], peroxisomal membrane-located PXA [14] and plasma membrane-located ABC transportors [15]. We performed local BLASTP analysis using characterized genes in petunia as a query and identified 14 *CpCATs*, two *CpPXAs*, one *CpEDS5* and 132 CpABC transporters respectively in wintersweet genome (Additional file 1: Table S23).

**Supplementary Note 3**

**Tandem duplication analysis**

To investigate tandem duplication events during the evolutionary course of wintersweet, we first searched for genome-wide duplicated genes and assigned them into four different modes with MCScanX analysis (Additional file 2: Figure S11). A total of 1,173 tandem gene pairs occupying 9.96% of the assembly were identified and assigned to 963 clusters with 11 genes in the longest cluster (Additional file 1: Table S24). Among the top three long clusters, the Leucine-rich repeat (LRR) genes are significantly enriched. Functional enrichment analysis by gene ontology (GO) terms and Kyoto Encyclopedia of Genes and Genomes (KEGG) analyses revealed that most genes were grouped into metabolic process and catalytic activity categories (Additional file 2: Figure S24a) and the genes involved in the phenylpropanoid biosynthesis pathway were found to be significantly enriched (Additional file 2: Figure S24b).

**The order of speciation and WGD event in *Liriodendron* and wintersweet lineage**

In order to find more evidence to verify the order between specification and duplication events shown by Ks peak distribution, the genes models of *Amborella trichopoda* and two Magnolia species (*Liriodendron* and wintersweet) were clustered using orthomcl. Then downstream program of MCScanX was employed to pick Magnolia genes belonging to blocks produced by duplication events. The orthogroups from syntenic blocks that contain one *Amborella trichopoda* gene, at least two wintersweet orthologs, and at least two *Liriodendron* orthologs were extracted. The CDS and protein sequences of these orthogroup were used to construct the ML phylogenetic trees by PhyML [16]. All possible topologies were summarized and utilized for the investigate the order between specification and duplication events in wintersweet and Liriodendron [17]. Based on these gene trees, we proposed two models: One possible speciation hypothesis (Model B), in which WGD happened before the speciation between wiuntersweet and *Liriodendron* (Additional file 2: Figure S14a); the alternative speciation scenario (Model A), in which WGD event occurred independently after the speciation between wiuntersweet and *Liriodendron* (Additional file 2: Figure S14b). If model A outperforms, orthologous gene pairs from Laurales and Liriodendron, respectively, would be grouped together; otherwise, paralogous genes of Laurales and Liriodendron would be grouped separately. In addition, Wilcox test was used to test the above two hypotheses. The results of these analyses indicated that the number of gene trees supporting Model B was significantly higher than that supporting Model A, suggesting wintersweet and *Liriodendron* experienced a WGD event respectively after their divergence from a common ancestor (Additional file 2: Figure S14).

**Reference**

1.Liu B, Shi Y, Yuan J, Hu X, Zhang H, Li N, et al. Estimation of genomic characteristics by analyzing k-mer frequency in de novo genome projects. arXiv preprint. arXiv:1308.2012.

2.Suen DF, Wang CK, Lin RF, Kao YY, Lee FM, Chen CC. Assignment of DNA markers to *Nicotiana sylvestris* chromosomes using monosomic alien addition lines. Theoretical and Applied Genetics. 1997;94:331-7.

3.Bouché F, Lobet G, Tocquin P, Périlleuxe C. FLOR-ID: an interactive database of flowering-time gene networks in *Arabidopsis thaliana*. Nucleic acids research. 2015;44:D1167-71.

4.Wheeler TJ, Eddy SR. nhmmer: DNA homology search with profile HMMs. Bioinformatics. 2013;29:2487-9

5.Edgar RC. MUSCLE: multiple sequence alignment with high accuracy and high throughput. Nucleic acids research. 2004;32:1792-7.

6.Livak KJ, Schmittgen TD. Analysis of relative gene expression data using real-time quantitative PCR and the 2^− ΔΔCT^ method. Methods. 2001;25:402-8.

7.Tian JP, Ma ZY, Zhao KG, Zhang J, Xiang L, Chen LQ. Transcriptomic and proteomic approaches to explore the differences in monoterpene and benzenoid biosynthesis between scented and unscented genotypes of wintersweet. Physiologia plantarum. 2019;166:478-93.

8.Sparkes IA, Runions J, Kearns A, Hawes C. Rapid, transient expression of fluorescent fusion proteins in tobacco plants and generation of stably transformed plants. Nature protocols. 2006;1:2019.

9.Ernst J, Bar-Joseph Z. STEM: a tool for the analysis of short time series gene expression data. BMC bioinformatics. 2006;7:191.

10.Hong GJ, Xue XY, Mao YB, Wang LJ, Chen XY V. *Arabidopsis* MYC2 Interacts with DELLA Proteins in Regulating Sesquiterpene Synthase Gene Expression. The Plant Cell. 2012;24:2635-2648.

11.Chuang YC, Hung YC, Tsai WC, Chen WH, Chen HH. *PbbHLH4* regulates floral monoterpene biosynthesis in *Phalaenopsis orchids*. Journal of experimental botany. 2018;69:4363-77.

12.Lynch JH, Orlova I, Zhao CS, Guo LY, Jaini R, Maeda H, et al. Multifaceted plant responses to circumvent Phe hyperaccumulation by downregulation of flux through the shikimate pathway and by vacuolar Phe sequestration. The Plant Journal. 2017;92:939-50.

13.Rekhter D, Lüdke D, Ding Y, Feussner K, Zienkiewicz K, Lipka V, Wiermer M, Zhang YL, Feussner L. Isochorismate-derived biosynthesis of the plant stress hormone salicylic acid. Science. 2019;365:498-502.

14.Widhalm JR, Dudareva N. A familiar ring to it: biosynthesis of plant benzoic acids. Molecular plant. 2015;8:83-97.

15.Adebesin F, Widhalm JR, Boachon B, Lefèvre F, Pierman B, Lynch JH, et al. Emission of volatile organic compounds from petunia flowers is facilitated by an ABC transporter. Science. 2017;356:1386-8.

16.Guindon S, Dufayard JF, Lefort V, Anisimova M, Hordijk W, Gascuel O. New Algorithms and Methods to Estimate Maximum-Likelihood Phylogenies: Assessing the Performance of PhyML 3.0. Systematic Biology. 2010;59:307-21.

17.Sun GL, Xu YX, Liu H, Sun T, Zhang JX, [Hettenhausen](https://www.nature.com/articles/s41467-018-04721-8#auth-6) C, et al. Large-scale gene losses underlie the genome evolution of parasitic plant *Cuscuta australis*. Nature Communication. 2018;9:2683.
