# Supplementary material for: The chromosome-level wintersweet (Chimonanthus praecox) genome provides insights into floral scent biosynthesis and flowering in winter
Source: Genome Biol. 2020 Aug 10;21:200. doi: 10.1186/s13059-020-02088-y (PMC7419205; doi:10.1186/s13059-020-02088-y)
Supplement: Supplementary file 4 — Additional file 4. Review history. [file 13059_2020_2088_MOESM4_ESM.docx]

Review History

**First round of review**

**Reviewer 1**

**Are you able to assess all statistics in the manuscript, including the appropriateness of statistical tests used?**

Yes, and I have assessed the statistics in my report.

**Comments to author:**

Please consider the following questions:

- Are the methods appropriate to the aims of the study, are they well described, and are necessary controls included? If not, please specify what is required.

The inference of whole-genome duplication part is not sufficient whether the laurels and magnoliales shared a whole-genome duplication or not. The evolutionary dating here is not enough to support their inference. As to the authors, time of the more ancient WGD event at around occurred 89.4 and 134.1 million years ago, being close to that in close to that in Liriodendron chinensis (116 MYA), and the divergence time of wintersweet and L. chinensis was near 120.6 MYA. Together with the involved synthetic analysis, the approaches involved here have no statistical power to let me ease about the conclusion. I have to suggest the authors to perform analysis here, possibly involving evolutionary dating with cross-genome collinear genes, and with phylogenetic analysis with collinear genes. The authors may refer to a publications by Wang J. et al on Mol Biol Evol. 2018 Jan; 35(1): 16-26., or Wang et al. on Plant Physiology!, January 2019, Vol. 179, pp. 209-219.

As to the inference of ancestral chromosomes and karyotypes, I do not find any methods that could support their analysis. Hope the authors could provide details of adopted methods. If not, may have to remove this part from the present manuscript.

- Are the conclusions adequately supported by the data shown? If not, please explain

The authors suggested that Magnoliids are sister to eudicots, and used trees to support their inference. However, it seems that the chloroplast tree does not agree with the trees constructed with nuclear genes. Involving more plants cannot provide stronger evidence. The authors have to explain this and may find other approaches, such as gene collinearity, to support their conclusion.

- Are sufficient details provided to allow replication and comparison with related analyses that may have been performed? If not, please specify what is required.

At least for the chromosome evolution and karyotype inference part, readers could not replicate the analysis.

- Does the work represent a significant advance over previously published studies?

Yes.

- Is the paper of broad interest to others in the field, or of outstanding interest to a broad audience of biologists?

Yes.

I have listed my major concerns above. Besides, the present manuscript has not been carefully prepared, and there is a lot room to improve. The structure of main text has to be improved. The section 2.4. and 2.5 have been misplaced. The section 2.4 may be merged with the gene annotation part above, which repeated analysis of GO and KEGG. Besides, the English needs a lot of improvement, and may have to be edited by English-native speakers and experts before resubmission.

As to the sequenced plant, any subspecies or cultivate name of the sequenced plant, which helps identify.

Background: any data of economic value, production, annually, and any data of medical use annually?

Lines 73-79: the sentences should be reorganized as to their logic.

Line 84: remove s in perfumes

Line 84: Apart from industrial utilization as spices agents, for wintersweet itself, the floral scent also functions: inconsistent subjects

Line 87: Despite the high economical and ecological value of floral scent in wintersweet, the genetic molecular mechanisms underlying the biosynthesis and regulation of the floral volatiles are less .... I do not think it is the scent of the economic value, and the sentence is not good in grammar.

Line 91: the sentence is also not good in grammar.

There are tons of grammatical problems.Nearly each sentence needs editing.

Line 147: software? pilon may be in uppercase. at least the first letter. and to be consistent to the other software citations.

Line 154: how do authors know that super-scaffolds are accurately clustered ???

Line 174: collected. past tense for human work description.

Line 204: 98% of all??

Line 208: remove "which is"

Line 217: the sentence is not right in meaning.

Line 219: which four species??

Line 217-224: the description is not clear to me.

Line 229: search for duplicated genes not for duplication.

Line 233-239: rewrite the sentences logically.

Line 245 and below: why 4Dtv and Ks are all used here, how about the evolutionary rate adopted???

Line 264: a 4:4 ratio cannot support whether they shared the whole-genome duplication or not, and competing models should be considered: sharing 2, sharing 1 and each having 1, each having 2 models should be considered. The following timing analysis is also not enough to support the conclusion.

Line 294: it is not wintersweet but magnolias to be sister to eudicots.

Line 323: to identify; too many identify used here

Line 326: remove that.

**Reviewer 2**

**Are you able to assess all statistics in the manuscript, including the appropriateness of statistical tests used?**

Yes, and I have assessed the statistics in my report.

**Comments to author:**

In this study, Shang et al. report the chromosome-level genome of wintersweet (Chimonanthus praecox). One of the key messages is the placement of magnolids in the pylogenetic tree. In addition, the authors also investigated the genes putatively associated with cold tolerance, flowering time, as well as secondary metabolite.

I appreciate the amount of works done in this study, but on the other hand, I feel a general lack of central message among these works. The manuscript could benefit from a re-write and re-organization of the contents. Specifically, for traits of focus such as cold tolerance, flowering time, and metabolite compounds (sections 2.7, 2.8, and 2.9), the authors chose to investigate the phylogeny and expression patterns of genes previously reported to be involved with cold tolerance in other species. However, while the results suggest these genes in wintersweet have consistent functions with their homologs in other species, this does not explain why wintersweet is particularly different in these traits from other species as the authors suggested. The Discussion section is also a re-description of Results and does not give a deeper meaning to those observations. I do appreciate the amount of works done and observations made, and I think it would be great to write this paper beyond the scope of observation and hypotheses generation.

The phylogenetic relationship among magnolids, eudicots, and monocots is one key information in this paper, but I do not see bootstrap value for Fig. 3a.

Please provide more details about Fig. 7c: Which plant species was used for transgenic experiments? How do the results translate into the biology of the focal species, if the experiments were done using Arabidopsis or tobacco?

I do not find the information as to what is the sample size of experiments in Figure 8.

The order in supplementary figures are wrong. Why are there two Fig S12 in front of S11, but no Fig S13?

Fig. 1 legend does not match the figure. Track "a" represents chromosomes in the figure but is denoted "626 flowering-time genes" in the legend. Also check other tracks.

Line 919: Please spell out 4DTv

Fig. 2b: The description is not clear. Is this showing the same Amborella region duplicated into 4 copies in Chimonanthus, or the rearrangement breaking a chromosomal chunk into 4 parts? Why picking the two specific regions, but not others, to highlight?

Fig. 2c: It would be great if the chromosome could be ordered as "number" not as "text"

Fig. 5a: It is unclear what is the purpose of this figure. For example, what is the staining used, and why the outer tissues have darker staining in panel 9?

Fig. 5c: What are the developmental stages?

Fig. 5b, 7a, S14a, S19, S21: What are the methods to generate the phylogenetic tree? Using DNA or amino acid sequences? How are the roots determined? What are the bootstrap support of branches? For Fig. 7a specifically, what are the different clades and differently colored external nodes?

Fig 7b: Please label which row represent which stage.

Fig. 7c: The panel label for "c" is missing. Is this a transgenic in this plant or in Arabidopsis?

Fig. S17: The first two sentences are repeating. Colored cluster are significant for what? What is the figure testing? This figure needs a lot more clarification.

Fig. S20: There are no "a" and "b" notation in the figure. What's the sample size of the expression test?

Line 85-87: Please add citation showing the scent has protective function against florivores and pathogens

Line 118-119: "small sample sizes of angiosperm species and inadequate sampling of taxa" - I would suggest putting in Discussion or Results, where the authors' result could support this.

Line 226-228: These sentences belong to Introduction.

Line 420: The citation should be in number.

Line 554: Define "the most advanced species"

**Authors Response**

**Point-by-point responses to the reviewers’ comments:**

*We sincerely appreciate the editors and reviewers for their thoughtful comments and recommendations on our manuscript. Those comments are very helpful for revising and improving our paper. We have studied the comments carefully and have revised the manuscript accordingly. We hope this revised manuscript will meet the journal’s high standards. The main amendments are highlighted in red font in the revised manuscript and the response to the reviewers’ comments are as follows:*

Point-by-point responses to the reviewers’ comments:

Reviewer #1:

Question: The inference of whole-genome duplication part is not sufficient whether the laurels and magnoliales shared a whole-genome duplication or not. The evolutionary dating here is not enough to support their inference. As to the authors, time of the more ancient WGD event at around occurred 89.4 and 134.1 million years ago, being close to that in close to that in Liriodendron chinensis (116 MYA), and the divergence time of wintersweet and L. chinensis was near 120.6 MYA. Together with the involved synthetic analysis, the approaches involved here have no statistical power to let me ease about the conclusion. I have to suggest the authors to perform analysis here, possibly involving, possibly involving evolutionary dating with cross-genome collinear genes, and with phylogenetic analysis with collinear genes. The authors may refer to a publications by on Mol Biol Evol. 2018 Jan; 35(1): 16-26., or y, January 2019, Vol. 179, pp. 209-219.

*Reply: We are grateful for this comment, and agree with the reviewer’s comments that the evidence we provided for the inference of the whole-genome duplication part were not sufficient. Following the reviewer’s suggestion, we have referred to several previous studies (Wang et al., 2018 Mol Biol Evol; Wang et al., 2019 Plant Physiology) and followed the approaches used in these papers, to analyze whether the Laurels and Magnoliales shared a whole-genome duplication or not. The major steps and data are as follows:*

*Firstly, We performed evolutionary dating analyses with both intra and inter-genomic collinear genes and found that the collinear genes between wintersweet and Liriodendron chinensis have various evolutionary rates (Additional file 2: Figure S13). After correction for the evolutionary rate (Yang et al., 2020 Nature Plants), the two polyploidization events in the genome of wintersweet (representative of Laurels) were estimated to occur at approximately 77.8 million and 112.2 million years ago (Ma), respectively; the polyploidization event in Liriodendron chinensis (representative of Magnoliales) was estimated to occur approximately 95.8Ma. Considering the divergence time of wintersweet and L. chinensis about 120.6 Ma, we proposed that the whole-genome duplication seem not shared by Laurels and Magnoliales (Figure 3c). In addition, we also conducted a phylogenetic analysis using collinear genes with reference to Sun et al., 2018 Nat Commun. The results also supported that the ancient whole-genome duplication in wintersweet occurred after the divergence between laurels and magnoliales (Additional file 2: Figure S14).*

*Here are four references cited in the revised manuscript:*

*Wang JP, Sun PC, Li YX, Liu YZ, Yang NS, Yu JG, et al. An Overlooked Paleotetraploidization in Cucurbitaceae. Mol Biol Evol. 2018;35:16-26.*

*Wang JP, Yuan JQ, Yu JG, Meng FB, Sun PC, Li YX, Yang NS, et al. Recursive Paleohexaploidization Shaped the Durian Genome. Plant Physiology. 2019; 179:209-19*

*Yang YZ, Sun PC, Lv L, Wang DL, Ru DF, Li Y, et al. Prickly waterlily and rigid hornwort genomes shed light on early angiosperm evolution. Nat plants. 2020;6:215–22.*

*Sun GL, Xu YX, Liu H. et al. Large-scale gene losses underlie the genome evolution of parasitic plant Cuscuta australis. Nat Commun. 2018; 9:2683.*

Question: As to the inference of ancestral chromosomes and karyotypes, I do not find any methods that could support their analysis. Hope the authors could provide details of adopted methods. If not, may have to remove this part from the present manuscript.

*Reply: To better explain the methods used for the inference of ancestral chromosomes and karyotypes, we have now provided more detailed explanations in the ‘Materials and Methods’ (‘5.8.Ancestral Genome Reconstruction’). An illustrative example of this approach can be found in the ancestor genome rescontruction literature (Murat et al., 2015 Genome Biology and Evolution; Murat et al., 2017 Nature Genetics). In addition, we also provided a modified pipeline for ancestral genome reconstruction (Additional file 2: Figure S15)*

*Here are the full titles for these two publications:*

*Murat F, Zhang RZ, Guizard S, Gavranović H, Flores R, Steinbach D, Quesneville H, Tannier E, Salse J, et al. Karyotype and Gene Order Evolution from Reconstructed Extinct Ancestors Highlight Contrasts in Genome Plasticity of Modern Rosid Crops. Genome Biology and Evolution. 2015;7:735–49.*

*Murat F, Armero A, Pont C, Klopp C, Salse J. Reconstructing the genome of the most recent common ancestor of flowering plants. Nature Genetics. 2017;49:490-6.*

Question: The authors suggested that Magnoliids are sister to eudicots, and used trees to support their inference. However, it seems that the chloroplast tree does not agree with the trees constructed with nuclear genes. Involving more plants cannot provide stronger evidence. The authors have to explain this and may find other approaches, such as gene collinearity, to support their conclusion.

*Reply: The phylogenetic incongruence between nuclear and organellar genomes has been recently reported in angiosperms. In comparison with nuclear genes, the plastid genes are uniparentally inherited and may recover different deep relationships resulting from incomplete lineage sorting (ILS) and hybridization (Yang et al., 2020 Nature Plants), which might potentially introduce biases and errors to phylogenetic reconstruction.*

*As sparse taxon sampling could result in conflicting conclusion, we increased our taxon samples in the nuclear phylogeny by adding more plants taxa. This analysis revealed the same relationships among Magnoliids, monocots and eudicots; suggesting that our results are robust with additional taxa sampling.*

*In the previous version of the manuscript, we constructed a phylogenetic tree based on the concatenated single-copy gene family alignments. While the concatenation method assumes that all genes have the same or similar evolutionary histories and implicitly ignore several complicated evolutionary realities, including gene tree conflict due to ILS and hybridization, which could appear more frequently when rapid radiations occur. Therefore, we applied the coalescent-based approaches that could incorporate (e.g.) individual gene–tree information to construct phylogenetic trees. The coalescent-based species trees were topologically identical to the one described above (Additional file 2: Figure S8), suggesting that our findings are reliable. To avoid potential errors in orthology inference, we also used SonicParanoid (Cosentino et al., 2019 Bioinformatics) to extract single-copy genes (SSCGs) and conducted concatenation and coalescent analyses based on these amino acid sequences of SSCGs. The species trees inferred from these analyses also supported the topology describe aboved. To summarize, we believe the phylogenetic relationship proposed in our study is relatively accurate under the current dataset.*

*Here are the full titles for these two publications:*

*Yang YZ, Sun PC, Lv L, Wang DL, Ru DF, Li Y, et al. Prickly waterlily and rigid hornwort genomes shed light on early angiosperm evolution. Nat plants. 2020;6:215–22.*

*Cosentino S, Iwasaki W. SonicParanoid: fast, accurate and easy orthology inference. Bioinformatics. 2019;35:149–51.*

Question: I have listed my major concerns above. Besides, the present manuscript has not been carefully prepared, and there is a lot room to improve. The structure of main text has to be improved. The section 2.4. and 2.5 have been misplaced. The section 2.4 may be merged with the gene annotation part above, which repeated analysis of GO and KEGG. Besides, the English needs a lot of improvement, and may have to be edited by English-native speakers and experts before resubmission.

*Reply: We apologize for the mistakes made in the initial manuscript. According to your suggestion, we invited native two English speakers to revise the manuscript thoroughly. We have also reorganized the structure of the main text in the revised version of our manuscript.*

Question: As to the sequenced plant, any subspecies or cultivate name of the sequenced plant, which helps identify.

*Reply: Thanks for your comment. There are several varieties of wintersweet. The plant material used in the report was generally considered to be Chimonanthus praecox var. intermedius. According to our previous morphological and molecular study, the so-called Chimonanthus praecox var. intermedius includes different genotypes, which are morphologically similar but genetically distinct. To avoid confusion, we use ‘H29’ in the study. This genotype is characterized by narrow, pale yellow middle tepals and dark red inner tepals.*

Question: Background: any data of economic value, production, annually, and any data of medical use annually?

*Reply: Wintersweet has great economic value. The flowers contain 0.5-0.6% essential oils, which are widely used for essential oils extraction [1]. For the medical use, the flowers are used in the treatment of thirst and depression whilst the essential oil is used to treat colds [2]. The leaves and roots can be used in the treatment of contusions, cuts, haemorrhages, strains, lumbago, rheumatism, numbness and colds [3]. Its annual production value reaches 100 millon dollars.*

*1. Feng JQ (2007) New Zealand flower industry–with special reference to wintersweet introduction and commercialization. Journal of Beijing Forestry University (suppl 1): 4–8.*

*2.Zhao Y, Zhang Y, Wang ZZ. Chemical composition and biological activities of essential oil from flower of Chimonanthus praecox (L.) Link. Lishizhen Medicine and Material Medical Research. 2010;21:622-625.*

*3.Usher G. Dictionary of Plants Used by Man. Constable. Macmillan Pub Co. 1974.*

Question: Lines 73-79: the sentences should be reorganized as to their logic.

*Reply: We have reorganized the logic of these sentences in revised version (lines 75-81).*

Question: Line 84: remove s in perfumes

*Reply: Thanks for your suggestion. We have made this change in the revised version.*

Question: Line 84: Apart from industrial utilization as spices agents, for wintersweet itself, the floral scent also functions: inconsistent subjects

*Reply: We have re-written this sentence as “Apart from broad industrial applications, the floral scent also functions in attracting and guiding pollinators to ensure its reproductive success, in addition to protecting the vulnerable reproductive organs from florivores and pathogens”.*

Question: Line 87: Despite the high economical and ecological value of floral scent in wintersweet, the genetic molecular mechanisms underlying the biosynthesis and regulation of the floral volatiles are less .... I do not think it is the scent of the economic value, and the sentence is not good in grammar.

*Reply: We agree with the reviewer. Here, the economical value of the floral scent is not suitable, we replaced the word ‘economical’ with ‘ornamental’ and modified the sentence in revised manuscript (lines 90-92).*

Question: Line 91: the sentence is also not good in grammar.

*Reply: We have fixed the grammatical errors and re-wrote this sentence in the revised manuscript (lines 92-94).*

Question: There are tons of grammatical problems. Nearly each sentence needs editing.

*Reply: Sorry for these typos in the initial manuscript. We have edited the sentences in the revised version.*

Question: Line 147: software? pilon may be in uppercase. at least the first letter. and to be consistent to the other software citations.

*Reply: pilon is the name of software. We have changed the words as suggested and added relevant citations.*

Question: Line 154: how do authors know that super-scaffolds are accurately clustered ???

*Reply: The HiC based heatmap was used as a criterion to measure the accuracy of super-scaffolds. We have included the Hi-C map in the Additional file 2: Figure S2. In this figure, continuous interactions between neighboring contigs/scaffolds were observed suggesting accurately clustered super-scaffolds.*

Question: Line 174: collected. past tense for human work description.

*Reply: Thanks. We have changed the word as suggested.*

Question: Line 204: 98% of all??

*Reply: Thanks. This phrase means 98% of TEs in the genic region. We have modified this phrase in the revised version (lines225).*

Question: Line 208: remove "which is"

*Reply: Thanks. We have removed these words.*

Question: Line 217: the sentence is not right in meaning.

*Reply: We have fixed this error in the revised version (lines 236-243).*

Question: Line 219: which four species??

*Reply: Here the four species are wintersweet (Chimonanthus praecox), Calycanthus chinensis, Liriodendron chinensis and Cinnamomum kanehirae. The full name of these four species is included in the revised version.*

Question: Line 217-224: the description is not clear to me.

*Reply: Sorry for this inconvenience. We have revised the sentences and hope it will give easy understanding (lines 236-244).*

Question: Line 229: search for duplicated genes not for duplication.

*Reply: Thanks. We have changed the words as suggested.*

Question: Line 233-239: rewrite the sentences logically.

*Reply: Thanks, as suggested, we have rewritten these sentences logically in the revised version (Additional file 3: lines 185-191).*

Question: Line 245 and below: why 4Dtv and Ks are all used here, how about the evolutionary rate adopted???

*Reply: In the revised manuscript, we have removed the 4Dtv. The evolutionary rate was obtained from previous study (Yang et al., 2020 Nature Plants).*

*Yang YZ, Sun PC, Lv L, Wang DL, Ru DF, Li Y, et al. Prickly waterlily and rigid hornwort genomes shed light on early angiosperm evolution. Nat plants. 2020;6:215–22.*

Question: Line 264: a 4:4 ratio cannot support whether they shared the whole-genome duplication or not, and competing models should be considered: sharing 2, sharing 1 and each having 1, each having 2 models should be considered. The following timing analysis is also not enough to support the conclusion.

*Reply: We thank you for pointing out this and agree with your suggestion that the synthetic analysis together with the timing analysis the in the initial manuscript is not sufficient to support the conclusion. Following your suggestions, we have redone this part and please referred to the reply above for the inference of whole-genome duplication part.*

Question: Line 294: it is not wintersweet but magnolias to be sister to eudicots.

*Reply: Thanks, as suggested, we have modified ‘wintersweet’ to ‘Magnolias’ as suggested.*

Question: Line 323: to identify; too many identify used here

*Reply: Thanks, as suggested, we have changed the words as suggested.*

Question: Line 326: remove that.

*Reply: Thanks, as suggested, we have removed this word.*

Reviewer #2:

-In this study, Shang et al. report the chromosome-level genome of wintersweet (Chimonanthus praecox). One of the key messages is the placement of magnolids in the pylogenetic tree. In addition, the authors also investigated the genes putatively associated with cold tolerance, flowering time, as well as secondary metabolite.

I appreciate the amount of works done in this study, but on the other hand, I feel a general lack of central message among these works. The manuscript could benefit from a re-write and re-organization of the contents. Specifically, for traits of focus such as cold tolerance, flowering time, and metabolite compounds (sections 2.7, 2.8, and 2.9), the authors chose to investigate the phylogeny and expression patterns of genes previously reported to be involved with cold tolerance in other species. However, while the results suggest these genes in wintersweet have consistent functions with their homologs in other species, this does not explain why wintersweet is particularly different in these traits from other species as the authors suggested. The Discussion section is also a re-description of Results and does not give a deeper meaning to those observations. I do appreciate the amount of works done and observations made, and I think it would be great to write this paper beyond the scope of observation and hypotheses generation.

*Reply: Thanks. It’s really a critical comment that will substantially improve our research. We thank the reviewer for the valuable suggestions. To make the manuscript more focused and concise, we have re-organized the ‘Results’ section [especially subsections “2.6 Genetic basis of floral transition, floral organs specification and early blooming in winter” (line 347-407); “2.7 Genetic basis of strong cold resistance” (line 408-420); “2.8 Evolution of terpene biosynthesis and regulation-related genes” (line 421-482); “2.9 Evolution of benzenoids/phenylpropanoids biosynthesis-related genes” (line 483-516)]. Furthermore, we also added additional analyses in section 2.6 and 2.7 in the revised manuscript) and re-draw several pictures (Figure. 2, 3, 4, 5, 6). In addition, we also revised the subsections “2.4 Phylogenomic placement of magnoliids sister to eudicots” (line 245-290) and “2.5 Whole-genome Duplication and Genome Evolution Analysis” (line 291-346) as suggested by the reviewer 1. For the discussion part, we have rewritten it according to the revision of the results, and gave a deeper meaning and novel insights to the observations instead of re-description of the results.*

-Question: The phylogenetic relationship among magnolids, eudicots, and monocots is one key information in this paper, but I do not see bootstrap value for Fig. 3a.

*Reply: Sorry for this negligence We have added the bootstrap value in the revised Fig. 2a.*

Question: Please provide more details about Fig. 7c: Which plant species was used for transgenic experiments? How do the results translate into the biology of the focal species, if the experiments were done using Arabidopsis or tobacco?

*Reply: Thanks for your suggestion. We used tobacco plants for transgenic experiments. From the transgenic experiments, we suggested that the CpTPS4 plays major role in production of linalool, the major component of the floral scent in wintersweet, which support the hypothesis that CpTPS4 is involved in the biosynthesis of scent in focal species-wintersweet here. We have added more details into the legend of Fig. 5 in the revised version of the manuscript.*

Question: I do not find the information as to what is the sample size of experiments in Figure 8.

*Reply: Sorry for this inconvenience. We conducted these experiments with three biological replicates. This information has been added to this legend. In the revised version, this figure is included in Figure5.*

Question: The order in supplementary figures are wrong. Why are there two Fig S12 in front of S11, but no Fig S13?

*Reply: We are sorry for the typo. We have corrected the order and added the Fig S13.*

Question: Fig. 1 legend does not match the figure. Track "a" represents chromosomes in the figure but is denoted "626 flowering-time genes" in the legend. Also check other tracks.

*Reply: Thanks. As suggestion, we have checked all the tracks and adjusted the Fig. 1 legend.*

Question: Line 919: Please spell out 4DTv

*Reply: Thanks, follow the advice of another reviewer, we have changed the method to analyze the WGD event and removed the 4DTv.*

Question: Fig. 2b: The description is not clear. Is this showing the same Amborella region duplicated into 4 copies in Chimonanthus, or the rearrangement breaking a chromosomal chunk into 4 parts? Why picking the two specific regions, but not others, to highlight?

*Reply: We apologized for this confusing description. It has been firmly established that there is no lineage-specific polyploidy events in Amborella (Albert et al., 2013 Science）. This figure derived from a comparative genomic analysis of wintersweet with Amborella which shows that some regions in Amborella have four corresponding “copies” in wintersweet which resulted from two whole genome duplication event. The two regions picked are the representatives and used to display the syntenic relationship between wintersweet and Amborella. We have now provided a clear description to address this in the legend of Fig. 3a in the revised version of manuscript.*

*Albert VA, Barbazuk WB, Der JP, Leebens-Mack J, Ma H, Palmer JD, et al. The Amborella genome and the evolution of flowering plants. Science. 2013;342: 1241089.*

Question: Fig. 2c: It would be great if the chromosome could be ordered as "number" not as "text"

*Reply: Thanks. As suggestion, we have ordered the chromosomes in number in Figure 3b in the revised manuscript.*

Question: Fig. 5a: It is unclear what is the purpose of this figure. For example, what is the staining used, and why the outer tissues have darker staining in panel 9?

*Reply: Thanks. This figure represents the key developmental stages of the floral bud which are also used as the reference for transcriptome construction. The staining used here was 1% safranine and 0.5% fast green. Under light microscope, it is common that some parts look darker because they absorb more dye. In the revised manuscript, this Figure is included in Figure4.*

Question: Fig. 5c: What are the developmental stages?

*Reply: According to the morphology of the floral bud, we divided the developmental stages into 9 key stages as shown in Figure4a (in the revised manuscript) including 1,undifferentiated flower bud (FBS1); 2, flower primordium formation (FBS2); 3, tepal primordium formation (FBS3); 4, stamen primordium formation (FBS4); 5, pistil primordium formation (FBS5); 6, flower organ development and differentiation stage (FBS6); 7, slow developmental stage (FBS7); 8, ovule appear (FBS8); 9, pollen formation (FBS9).*

Question: Fig. 5b, 7a, S14a, S19, S21: What are the methods to generate the phylogenetic tree? Using DNA or amino acid sequences? How are the roots determined? What are the bootstrap support of branches? For Fig. 7a specifically, what are the different clades and differently colored external nodes?

*Reply: Thanks. These phylogenetic trees were all constructed by MegaX using the amino acid sequences alignments generated by ClustalX. The detailed procedure is mentioned in the “Method” section (line834-836). The roots were determined according to the different subfamilies which the genes belong to. In Fig. 7a, the different clades represent the subfamilies of the TPS gene family and the differently colored external nodes represent different species. We have now added this information in the legend of Fig. 5 in the revised manuscript.*

Question: Fig. 7b: Please label which row represent which stage.

*Reply: Thanks for your suggestion. We have added the required modifications in the revised version. In the revised manuscript, this Figure is included in Figure5.*

Question: Fig. 7c: The panel label for "c" is missing. Is this a transgenic in this plant or in Arabidopsis?

*Reply: We are sorry for overlooking the labeling of the Figure. We have now added the notion "c" and the transgenic plant is tobacco. In the revised manuscript, this Figure is included in Figure5.*

Question: Fig. S17: The first two sentences are repeating. Colored cluster are significant for what? What is the figure testing? This figure needs a lot more clarification.

*Reply: Thanks. As suggested, we have removed the repeated sentence and added more detailed description in the legend. Clusters in color indicate the expression level of TFs is significantly different between different stages (p value ≤ 0.05). This figure represents the differently expressed TFs. In the revised manuscript, this Figure is included in supplementary Figure S19.*

Question: Fig. S20: There are no "a" and "b" notation in the figure. What's the sample size of the expression test?

*Reply: Thanks. We have now added the notation in the Fig. S20. This expression test was carried out with three biological replicates. This figure has been included in Figure 4c as a whole in the revised manuscript.*

Question: Line 85-87: Please add citation showing the scent has protective function against florivores and pathogens

*Reply: Thanks. We have added a reference at the required place (line 90).*

Question: Line 118-119: "small sample sizes of angiosperm species and inadequate sampling of taxa" - I would suggest putting in Discussion or Results, where the authors' result could support this.

*Reply: Thanks. We have moved these sentences to Results (line 279-280).*

Question: Line 226-228: These sentences belong to Introduction.

*Reply: Thanks. We have removed these sentences.*

Question: Line 420: The citation should be in number.

*Reply: Thanks. We have made this change in the revised version.*

Question: Line 554: Define "the most advanced species"

*Reply: Because this description is not sufficiently supported, we have now deleted the statement.*

**Second round of review**

**Reviewer 1**

May remove "also" in line 308.

Line 563: "blooms" to "bloom"

Line 1291: a blank is needed between two words. Please check the references to ensure consistency.

**Reviewer 2**

Main comments

In this version, Shang et al. have revised the manuscript. While some changes were made, I am afraid the magnitude of changes may not be sufficient.

The main logic in this revision remains the same: The authors seem to have a prior belief of what is "special" about this species and then looked for homologs that were reported to be associated with these traits in other species, finally made a description about their expression patterns and location in the wintersweet genome. The same logic is used for Fig4 (flowering time), Fig5 (terpene), and Fig6 (benzenoids/phenylpropanoids).

As mentioned in the previous review, these were merely observed patterns that do not explain why wintersweet is particularly unique in flowering time or secondary metabolites. The claim of gene function, if true, is at best showing wintersweet homologs have consistent function with Arabidopsis, without providing much novelty or explaining the main claim of this paper (wintersweet is special in its winter flowering and secondary metabolites).

For example, what do Fig 4, Fig 5, and Fig 6 tell us? These graphs describe known biosynthetic pathways and place the known genes' homologs in wintersweet genome, but it is unclear whether showing the expression patterns of these homologs in different developmental stages reveal anything. Does higher/lower expression of some homologs of some genes in some developmental stages result in the particular phenotypes of wintersweet? I do not find such information in the manuscript. In other words, these graphs could be placed in the genome paper of any other species, even those without particular flowering and secondary metabolite patterns.

Other comments

The authors have deposited their assembled genome in NCBI, but please also make sure to deposit the original read data (long-read, short-read, HiC short-read, and RNAseq reads) under the same bioproject. Please also provide the NCBI "reviewer link" of the bioproject for the reviewers to confirm all data have been deposited (and labeled correctly) and will go public once the paper is published.

About the bootstrap value for Figure 2a: The figure resolution is just too bad and the font too small to let me see the values, especially the most important branch grouping Magnoliids and Eudicots.

Line 320-322: I do not see how Fig 3c reports the two time points.

Line 359-363: Given the high difference in gene content and long divergence time, how can one be sure that the identified homologs have the same function in wintersweet as in Arabidopsis?

Line 838-840: The tree reconstruction method is still not clear. Using protein sequence, so I would assume that is a neighbor-joining tree. Such distance-based tree is not a good choice to deal with more ancient relationship such as the history of gene families that predate the divergence of monocots, eudicots, and Magnoliids.

In general, I feel the figure legends are even more error-prone than the previous version.

Line 870: 5Mb

Line 883: 1Mb "intervals" ?

Line 885: Blue numbers denote divergence time. Those in the brackets are what? 95% CI ?

Line 888: Is it the proportion of genes or "gene families" undergoing gain and loss? The use of red/green is very colorblind unfriendly.

Line 889: Numbers "below"

Line 890-891: My understanding is that only 1-to-1 genes are orthologs. Multi-copy ones are only homologs. What is a "unique ortholog"?

Line 892: Do the numbers represent genes or gene families? I am not aware of this term "orthologous gene family".

Line 894-895: There is parenthesis in this figure at all.

Line 900: I would suggest to change all red vs. green colors in all figures.

Figure 3b: This generally feels a bit cherry-picking. The authors picked the regions matching the expected 4-4 pattern to highlight. In the meantime, there are many 1-1 regions in the dotplot between Chimonanthus and Cinnamomum. Why couldn't one label those 1-1 regions and say there's no whole-genome duplication? If this figure is the only support for the claim of a 4-4 relationship, I think we would need something more solid to support this claim.

Figure 3c: The legend on the upper right hand side is very difficult to see. My understanding is that "ortholog" refers to strict one-to-one relationship between genomes. When one compares orthologs between genomes, there would only be one peak reflecting the speciation time. Why are there generally two peaks for the solid line representing orthologs?

Line 907: The duplication events were shown by 3 dots of different colors, not "red colored dots"...

Line 908-910: Rephrase to make it more clear.

Line 911-912: The figure title does not reflect the content of these panels at all.

Line 922-923: But the figure says the opposite: green is down-regulated, and yellow-green is up-regulated. There is no orange.

Figure 4b: Where does this network comes from? Is this based on the flowering time network of Arabidopsis? How much evidence does the authors have to be certain that the flowering time pathway is similar to Arabidopsis? Given the multiple rounds of WGD and gene-specific duplication and loss between Arabidopsis and wintersweet, how many more homologs do wintersweet have? It is a bit difficult to believe the same flowering time pathway can be directly applied to wintersweet.

Line 923: According to the graph, lost genes are in white boxes, not "dotted boxes".

Line 927-928: But for the most famous MADS gene, FLC, all circles are green. I believe Arabidopsis thaliana has only one FLC. Fig4c also shows Arabidopsis has 6 copies of SOC1? I do not think so.

Line 929: I do not see the +- SE in the figure?

Line 940-941: This is a very strange sentence. The genes in green and WHAT were generated by WGD and tandem duplication events, respectively?

Figure 5a: Where does the pathway information come from? Also Arabidopsis? Here the authors have correctly labeled duplicated homologs and shown their expression patterns separately. This further makes me wonder whether Figure 4b is correct: non of those flowering time genes were duplicated in wintersweet?

Figure 5b: This phylogenetic tree looks very strange to me. In several cases (clades b, g, and part of a), Arabidopsis is the outgroup, and C. praecox, S. bicolor, and C. chinensis genes are all thoroughly mixed together. This seems to suggest Eudicot is more distant from Magnoliids and Monocots. How is the root determined? Who is the outgroup?

Line 944: Again I do not see any +- SE information in Figure 5c. The figure shows the pattern of one replicate, right? Or the averaged signal of 3 replicates?

Figure 5d: How many CpTPS genes copies are there, and why only showing copy 1-20?

Figure 5, e & f: legend and figure are opposite.

Figure 6a: What are the genes boxed by red and green boxes? And what are the non-boxed genes?

Figure 6b, left panel: What are the grey bands connecting between chromosomes?

**Authors Response**

**Point-by-point responses to the reviewers’ comments:**

*We sincerely appreciate the editors and reviewers for their thoughtful comments and recommendations on our manuscript. Those comments are very helpful for improving our paper. We have studied the comments carefully and revised the manuscript throughly. We believed that most major concerns raised by the reviewers have been addressed and hope this revised manuscript will meet the journal’s high standards. The main amendments are highlighted in red font in the revised manuscript and the Reply to the reviewers’ comments are as follows:*

Point-by-point Replys to the reviewers’ comments:

Reviewer #1:

Question: May remove "also" in line 308.

*Reply: Thanks. We have removed this word.*

Question: Line 563: "blooms" to "bloom"

*Reply: Thanks. We have changed the word as suggested.*

Question: Line 1291: a blank is needed between two words. Please check the references to ensure consistency.

*Reply: Thanks. We have added a blank between the two words (‘Reconstructed’ and ‘Extinctand’). We also have checked the references throughly.*

Reviewer #2: Main comments

In this version, Shang et al. have revised the manuscript. While some changes were made, I am afraid the magnitude of changes may not be sufficient.

The main logic in this revision remains the same: The authors seem to have a prior belief of what is "special" about this species and then looked for homologs that were reported to be associated with these traits in other species, finally made a description about their expression patterns and location in the wintersweet genome. The same logic is used for Fig4 (flowering time), Fig5 (terpene), and Fig6 (benzenoids/phenylpropanoids).

As mentioned in the previous review, these were merely observed patterns that do not explain why wintersweet is particularly unique in flowering time or secondary metabolites. The claim of gene function, if true, is at best showing wintersweet homologs have consistent function with Arabidopsis, without providing much novelty or explaining the main claim of this paper (wintersweet is special in its winter flowering and secondary metabolites).

For example, what do Fig 4, Fig 5, and Fig 6 tell us? These graphs describe known biosynthetic pathways and place the known genes' homologs in wintersweet genome, but it is unclear whether showing the expression patterns of these homologs in different developmental stages reveal anything. Does higher/lower expression of some homologs of some genes in some developmental stages result in the particular phenotypes of wintersweet? I do not find such information in the manuscript. In other words, these graphs could be placed in the genome paper of any other species, even those without particular flowering and secondary metabolite patterns

*Reply: We are thankful to the reviewer for his critical comments and regret the confusing description of the logic. In the following part, we would like to introduce the novelty and newly added data in our present work as well as the explanation for the novelty in the content of Fig 4, Fig 5, and Fig 6 compared to the previous publications.*

*Wintersweet (Chimonanthus praecox) is a popular ornamental plant known for its fragrant aroma and winter-flowering properties. As a representative of the Magnoliidae, it also has a vital significance in the evolutionary position. In our present work, we present a high–quality genome sequence of wintersweet at chromosome-level using integrated approaches (Illumina HiSeq, 10X Genomics, PacBio SMRT sequencing and Hi-C) and a draft genome assembly of Calycanthus chinensis. Taking advantage of these two genomes, we performed phylogenetic analyses and provided a solid topology supporting that Magnoliids and eudicots are sister to monocots. Whole-genome duplication (WGD) signatures revealed 2 major duplication events in the evolutionary history of the wintersweet genome, with an ancient one shared by Laurales, and a more recent one shared by the Calycantaceae. WGD and tandem duplication events had significant impacts on the copy numbers of genes related to terpene and benzenoids/phenylpropanoids (the main floral scent volatiles) biosynthesis, especially the terpene synthase (TPS) and benzyl alcohol acetyltransferase (BEAT) genes. Muti-omics analyses revealed that the strong fragrance of wintersweet can be attributed to a species-specific expansion and unique high-level expression of genes in the TPS-b subfamily and BEAT genes. In addition, an integrative analysis that combines cytology with genome and transcriptome data revealed major developmental biology of wintersweet, such as floral transition in spring, floral organs specification, low temperature-mediated floral bud break, early blooming in winter, and strong cold tolerance. Our study not only provides insights into wintersweet-specific biology and will provide a fundamental resource for functional genomics research and molecular breeding of wintersweet.*

*Our present work is mainly based on high–quality genome data. For the biological aspects we also integrated the transcriptome/metabolom data to identify some candidate genes which are associated with the two special traits (flowering and fragrance). In addition to obtain the expression patterns information about this candidate genes, from the genome-scale, we also investigated the evolution of these genes and try to find some molecular evidences such as gene expansion and loss by comparative genomics analyses which cause dosage changes and influence the flowering process. Our main novelty or insight for the special trait of wintersweet are as follows:*

*(1) For the flowering part:*

*Flowering is a complex developmental process that involves floral initiation, meristem specification, floral organs specification, floral bud dormancy and release, and blooming. Wintersweet is one of the perennial trees that bloom in the deep winter, however, its flower bud initiation and floral transition complete in April. This is an very unique flowering model compared to Arabidopsis and rice, and there has no any report regarding its molecular mechanism based on the genome data. To investigate the mechanism underlying the whole process that may influence the final flowering time, we performed a systematic study on the floral ontogeny and developmental patterns by paraffin sections through microscopic observation. Based on the phenotypic changes, we divided the whole process into 11 stages and generated 33 transcriptomes for corresponding stages from the time of floral initiation to maturation (Figure 4a and Additional file 2: Figure S16) and we believe this part is one of the major novelties of this report and was not reported in any other model plant species. Based on the division of these 11 stages, we performed the transcriptome and phenotypic analyses and uncovered the specific molecular mechanism underlying the critical flower developmental stages in wintersweet. In fact, we have identified plenty of differentially expressed genes among different stages by comparative transcriptome analysis. Then, we mainly focused on the homologous genes which are associated with the corresponding phenotype and have been reported to have conserved function across different plant lineages (not limited to Arabidopsis) (Figure 4b and Additional file 1: Table S13). Our analysis showed that the endogenous hormone (such as gibberellin) and environmental factor (photoperiod) play a major role in the switch from vegetative to reproductive growth; moreover, the slow growth of flower organ in summer is due to low expression levels of cell division-related genes which are regulated by Heat stress transcriptional factors (HSFs) (Figure 4b and Additional file 1: Table S16). In addition, the key components of the genetic network (e.g. SVP, FT) for seasonal temperature-mediated control of vegetable bud break in wintersweet displayed a similar response to the same environmental cues (low temperature) as those in model plant hybrid aspen (Additional file 2: Figure S16), leading us to the hypothesis that wintersweet potentially harness the similar genetic pathway in floral bud break control.*

*To better understand the dynamic of MADS-box genes evolution in wintersweet and to facilitate future research on this important gene family which has a conserved function in floral organ specification and flowering time regulation, we provided a detailed overview of the number, phylogeny and expression of MIKC-type MADS-box genes in wintersweet genome. We found that the number of AGL6 subfamily is greater than that in rice and Arabidopsis and gene loss in the FLC subfamily (Figure 4c). AGL6 and FLC have been reported to serves as a flowering promoter and repressor respectively (Ruelens et al., 2013 Nature Communication; Wang et al., 2011 Journal of Plant Growth Regulation). Therefore, we hypothesize that selective expansion and contraction in those subfamilies markedly affect the flowering time. Further, large scale RNA-seq analyses and RT-PCR analyses provided insights into expression patterns of homologous genes at different development stages and different floral organs; building a rich resource for more detailed analyses. The broad expression of the B-class genes eventually lead to the petaloid perianth formation (Figure 4c).*

*(2) For the floral scent part:*

*Different from most model plants in which the floral scent mainly consist of either terpene (such as in Arabidopsis) or benzenoids/phenylpropanoids (such as in Petunia), the flower of wintersweet possesses both of these two classes of compounds with linalool and benzyl acetate as the characteristic components. Based on the genomic, transcriptomic and metabolomic data, we performed a comprehensive investigation of the formation, regulation and transportation of the floral scent. Firstly, we utilized complementary tools to discover metabolic genes encoded all known enzymic steps in the volatile terpenes and benzenoids biosynthesis and transport (Figure 5a and 6a), after that the evolution of these genes was also analyzed. We provided evidence that WGD and tandem duplication events had major impacts on the copy numbers of genes involved in terpene and benzenoids/phenylpropanoids biosynthesis, particularly TPS and BEAT genes (Figure 5a,b,d and 6b), which are critical for the production of principal components of floral scent (linalool and benzyl acetate). The expression level of biosynthesis genes are usually consistent with the production of the secondary metabolites, so we combined the expression patterns of the functional genes involved in the biosynthesis pathway of the characteristic aroma with the metabolic data at different flower developmental stages and tried to find the candidate genes responsible for the production of characteristic aroma (linalool and benzyl acetate) of wintersweet (Figure 5c and 6c). Using this approach, we succeed in identifying the CpTPS4 gene which is responsible for the linalool (main component of the floral scent) production (Figure 5e and 5f). Furthermore, co-linear and phylogeny analysis of the TPS family revealed significantly expanded in the TPS-b subfamily and several genes are derived from the tandem duplication (Figure 5d). These data are unique in wintersweet and not shown in any model plants species before.*

*The CpTPS4 gene has three copies, all of which were highly expressed and found to be arranged in cluster. As reported in a recent publication (Salamov et al., 2010 Genome Research), the tandem arrangement could contribute to increase the transcript abundance of the tandem duplicates. Therefore, the mass linalool production in wintersweet contributed to the tandem duplication. Based on these results, we propose that selective expansion of the TPS gene family through remarkable duplication, gene cluster formation, gene functional differentiation, and gene regulation divergence dramatically contribute to the abundant characteristic aroma formation in wintersweet. These results are also highly specific in the wintersweet genome and there has very limited information in model plant species.*

*In summary, our present work builds an array of resources to understand the important biological traits of wintersweet. We have performed a comprehensive investigation of the whole progress of flowering and provided multiple dimensions evidence for the explanation in the unique flowering time which lay a foundation for further functional genomics research in wintersweet. In addition, for the special trait in strong fragrance, the molecular mechanism which resulted in the abundant characteristic aroma formation (linalool) has been uncovered for the first time in wintersweet. Based on these data, we do believe this report provides considerable novel insights for the flowering time and secondary metabolite of scent of the flowers.*

*We sincerely hope that you can understand and accept our response.*

*Salamov AA, Solovyev VV. Ab initio gene finding in Drosophila genomic DNA. Genome Research. 2000;10:516-22.*

*Wang BG, Zhang Q, Wang LG, Duan K, Pan AH, Tang XM, et al. The AGL6-like Gene CpAGL6, a Potential Regulator of Floral Time and Organ Identity in Wintersweet (Chimonanthus praecox). Journal of Plant Growth Regulation. 2011;30: 343-52.*

*Ruelens P, De Maagd RA, Proost S, Theien G, Geuten K, Kaufmann K. FLOWERING LOCUS C in monocots and the tandem origin of angiosperm-specific MADS-box genes. Nature Communications. 2013;4:2280.*

Question: The authors have deposited their assembled genome in NCBI, but please also make sure to deposit the original read data (long-read, short-read, HiC short-read, and RNAseq reads) under the same bioproject. Please also provide the NCBI "reviewer link" of the bioproject for the reviewers to confirm all data have been deposited (and labeled correctly) and will go public once the paper is published.

*Reply: All the data described and discussed in this manuscript, including whole-genome assembly data (long-read, short-read, HiC short-read), and transcriptome data of different flower developmental stages have now been submitted to the National Center for Biotechnology Information (NCBI) under accession codes PRJNA600650.*

*The reviewer links are as follows: (https://dataview.ncbi.nlm.nih.gov/object/PRJNA600650?reviewer=osj7bv4e6jtakiqetakqdfd0va,https://dataview.ncbi.nlm.nih.gov/object/PRJNA600650?reviewer=osj7bv4e6jtakiqetakqdfd0va).*

Question: About the bootstrap value for Figure 2a: The figure resolution is just too bad and the font too small to let me see the values, especially the most important branch grouping Magnoliids and Eudicots.

*Reply: Thanks. We have adjusted the font size and the figure resolution as suggested in the revised manuscript. The bootstrap value for the branch grouping Magnoliids and Eudicots is 93%.*

Question: Line 320-322: I do not see how Fig 3c reports the two time points.

*Reply: Thanks. The two-time points were calculated using the synonymous substitutions per site per year (r = 4.21×10-9) and the Ks value in the peak as shown in Figure 3c. The calculating formula used here is WGD time T = Ks / 2r.*

*We have rewritten the sentence in the revised version (lines 320-323).*

Question: Line 359-363: Given the high difference in gene content and long divergence time, how can one be sure that the identified homologs have the same function in wintersweet as in Arabidopsis?

*Reply: Thanks. The functional characterization of genes in plants always is started from model plants such as Arabidopsis and Populus. Most of the genes have conserved function across land plants. We took advantage of the database of flowering-time gene networks in Arabidopsis to construct a proposed network in wintersweet for further functional validation. The same method is also used in the flowering study in the sunflower genome (Badouin et al., 2020 Nature).*

*Badouin H, Gouzy J, Grassa C J, et al. The sunflower genome provides insights into oil metabolism, flowering and Asterid evolution. Nature, 2017, 546(7656): 148-152.*

Question: Line 838-840: The tree reconstruction method is still not clear. Using protein sequence, so I would assume that is a neighbor-joining tree. Such distance-based tree is not a good choice to deal with more ancient relationship such as the history of gene families that predate the divergence of monocots, eudicots, and Magnoliids.

*Reply: Thanks. As suggested, we have now reconstructed the phylogenetic trees of functional genes by the maximum likelihood method using the amino acid sequences alignments generated by ClustalX. We also adjusted the sentence in the revised version (lines 870-872).*

Question: Line 870: 5Mb

*Reply: Thanks. We have changed the word as suggested.*

Question: Line 883: 1Mb "intervals" ?

*Reply: Yes. Here is "intervals" not "internals". We have changed the word.*

Question: Line 885: Blue numbers denote divergence time. Those in the brackets are what? 95% CI ?

*Reply: Those numbers in brackets are 95% confidence intervals for the time of divergence between different clades. We have added this information to the legend of Figure 2.*

Question: Line 888: Is it the proportion of genes or "gene families" undergoing gain and loss? The use of red/green is very colorblind unfriendly.

*Reply: Thanks. Here is the proportion of "gene families" not "genes" that undergo gain and loss. We have adjusted the word "genes" to "gene families" and the color "red/green" has been changed.*

Question: Line 889: Numbers "below"

*Reply: Thanks. We have changed the word as suggested.*

Question: Line 890-891: My understanding is that only 1-to-1 genes are orthologs. Multi-copy ones are only homologs. What is a "unique ortholog"?

*Reply: Thanks. Yes. The single-copy orthologs include common orthologs with one copy in specific species. Multi-copy orthologs include common orthologs with multiple copy numbers in specific species. Other orthologs include genes from families shared in 2-16 species. Unique include genes of specie special gene family. This classification method was also applied in the Gastrodia elata genome study (Yuan et al., 2018 Nature Communication).*

*Yuan Y, Jin X, Liu J, et al. The Gastrodia elata genome provides insights into plant adaptation to heterotrophy. Nature Communications, 2018, 9(1): 1615-1615.*

Question: Line 892: Do the numbers represent genes or gene families? I am not aware of this term "orthologous gene family".

*Reply: Thanks. We are sorry for this confusing description. The numbers represent gene families not "orthologous gene family". We have now rewritten the description in Figure2 legend (line 935-936).*

Question: Line 894-895: There is parenthesis in this figure at all.

*Reply: Thanks. Yes, there is no parenthesis in the figure. We are sorry for this mistake. Now we have rewritten the description in the legend of Figure2.*

Question: Line 900: I would suggest to change all red vs. green colors.

*Reply: Thanks. We have changed all red vs. green colors in all figures as suggested.*

Question: Figure 3b: This generally feels a bit cherry-picking. The authors picked the regions matching the expected 4-4 pattern to highlight. In the meantime, there are many 1-1 regions in the dotplot between Chimonanthus and Cinnamomum. Why couldn't one label those 1-1 regions and say there's no whole-genome duplication? If this figure is the only support for the claim of a 4-4 relationship, I think we would need something more solid to support this claim.

*Reply: Thanks. The distribution of Ks (synonymous substitution rate) among paralogous genes within the wintersweet genome displayed two clear peak (Figure 3c) suggesting two whole-genome duplication (WGD) events have occurred during wintersweet genome evolution. Intergenomics co-linearity analysis also supported two WGD events, as indicated by a 4:1 syntenic relationship between the wintersweet and the Amborella genome (Figure 3a). To further elucidate the polyploidy of wintersweet genomes, we also performed the comparative genomic analysis between the wintersweet and Cinnamomum genome. Since the Cinnamomum genome has undergone two WGD events (Chaw et al., 2019 Nature plants), the large scale 4-4 regions in dot-plot shown in figure 3b is another support for the two WGD events in wintersweet.*

*Chaw S M, Liu Y C, Wu Y W, et al. Stout camphor tree genome fills gaps in understanding of flowering plant genome evolution. Nature plants, 2019, 5(1): 63-73.*

Question: Figure 3c: The legend on the upper right hand side is very difficult to see. My understanding is that "ortholog" refers to strict one-to-one relationship between genomes. When one compares orthologs between genomes, there would only be one peak reflecting the speciation time. Why are there generally two peaks for the solid line representing orthologous ?

*Reply: Thanks. To make it clear, we have expanded the size of the legend in the Figure3c. We are sorry for the incorrect description. In fact, the solid line represents the paralogous and the dashed line represents the orthologous.*

Question: Line 907: The duplication events were shown by 3 dots of different colors, not "red colored dots"...

*Reply: Thanks. We have adjusted these words as suggested (line 950).*

Question: Line 908-910: Rephrase to make it more clear.

*Reply: Thanks. We have rephrased this sentence (line 951-954).*

Question: Line 911-912: The figure title does not reflect the content of these panels at all.

*Reply: Thanks. We have now changed the title (line955-956).*

Question: Figure 4b: Where does this network comes from? Is this based on the flowering time network of Arabidopsis? How much evidence does the authors have to be certain that the flowering time pathway is similar to Arabidopsis? Given the multiple rounds of WGD and gene-specific duplication and loss between Arabidopsis and wintersweet, how many more homologs do wintersweet have? It is a bit difficult to believe the same flowering time pathway can be directly applied to wintersweet.

*Reply: Based on the flowering-time network of Arabidopsis, we constructed the proposed flowering-time network of wintersweet. The same method also used in the flowering investigation in the sunflower genome (Badouin et al. 2017 Nature). In fact, the function of most genes in the network has been characterized in some non-model plants and reported to have the conserved function as that in Arabidopsis, especially the genes in phytohormone and photoperiod pathway.*

*It is hard to correctly label the duplicated homologs and show their expression patterns separately in figure 4b, so we list the duplicated homologs and their expression level in the supplementary (Additional file 1 Supplementary Table S13). We have removed this Figure 4b in the revised version.*

Question: Line 922-923: But the figure says the opposite: green is down-regulated, and yellow-green is up-regulated. There is no orange.

*Reply: Thanks. We have now removed Figure 4b and the corresponding description.*

Question: Line 923: According to the graph, lost genes are in white boxes, not "dotted boxes".

*Reply: Thanks. We are sorry for this mistake. We have changed the word as suggested.*

Question: Line 927-928: But for the most famous MADS gene, FLC, all circles are green. I believe Arabidopsis thaliana has only one FLC. Fig4c also shows Arabidopsis has 6 copies of SOC1? I do not think so.

*Reply: Based on the similarity of the protein sequences, the MADS-box gene family was divided into several subfamilies. The subfamily is usually named as one of its members’ name whose function has been characterized. Compared with other subfamilies, the members of the same subfamily have higher similarity and tend to have a similar function. The six genes from Arabidopsis clustered in the SOC1 subfamily only means they have higher similarity in the protein sequence. We may not regard them as different copies.*

Question: Line 929: I do not see the +- SE in the figure?

*Reply: Thanks. Since the figure here is the heatmap representing the gene expression patterns of MIKCc, the sentence "Data are presented as means (±SE, n = 3) " is superfluous. We have removed this sentence.*

Question: Line 940-941: This is a very strange sentence. The genes in green and WHAT were generated by WGD and tandem duplication events, respectively?

*Reply: We are sorry for the confusing description. The right sentence is "The genes circled in green were generated by WGD events.*

Question: Figure 5a: Where does the pathway information come from? Also Arabidopsis? Here the authors have correctly labeled duplicated homologs and shown their expression patterns separately. This further makes me wonder whether Figure 4b is correct: non of those flowering time genes were duplicated in wintersweet?

*Reply: Thanks for your valuable comment. Yes. The pathway information comes from Arabidopsis.*

*As we have descriped in the previous reply. It is indeed hard to correctly label the duplicated homologs and show their expression patterns separately in figure 4b, so we list the duplicated homologs and their expression level in the supplementary (Additional file 1 Supplementary Table S13) and removed Figure 4b in the revised version.*

Question: Figure 5b: This phylogenetic tree looks very strange to me. In several cases (clades b, g, and part of a), Arabidopsis is the outgroup, and C. praecox, S. bicolor, and C. chinensis genes are all thoroughly mixed together. This seems to suggest Eudicot is more distant from and Monocots. How is the root determined? Who is the outgroup?

*Reply: The gene phylogeny not following the species phylogeny in some subclades may be mainly due to the different evolutionary rate of genes. The selective expansion in the TPS-b subfamily, suggesting the natural selection of these genes within these subfamilies which may lead to the different evolutionary rate of these genes. This inconsistent between gene phylogeny and species phylogeny was also observed in the TPS phylogenetic tree in the Cinnamomum genome study (Chaw et al., 2019 Nature plant). We have constructed the phylogenetic tree by adding more sequences from different species and obtained a similar result (the figure as follows).*

*It is usually hard to determine the root when multiple subfamilies exist in one family. So, we reconstructed an unrooted tree in the revised version of the manuscript. The genes in different sub-families of TPS genes usually have different functions, such as the genes in TPS-b clade are either monoterpene synthases or isoprene synthases (Chen et al., 2011 Plant Journal). We constructed this tree for subfamily classification and selecting the genes for further functional validation.*

*Chaw S M, Liu Y C, Wu Y W, et al. Stout camphor tree genome fills gaps in understanding of flowering plant genome evolution. Nature plants, 2019, 5(1): 63-73.*

*Chen F, Tholl D, Bohlmann J, et al. The family of terpene synthases in plants: a mid-size family of genes for specialized metabolism that is highly diversified throughout the kingdom. Plant Journal, 2011, 66(1): 212-229*

Question: Line 944: Again I do not see any +- SE information in Figure 5c. The figure shows the pattern of one replicate, right? Or the averaged signal of 3 replicates?

*Reply: This figure represents mass spectrogram of products of recombinant CpTPS4, CpTPS9 and CpTPS42 proteins and shows the pattern of one replicate not the averaged signal of 3 replicates. The 3 replicates have the same signal. So, the sentence "Data are presented as means (±SE, n = 3) " is superfluous. We are sorry for the confusing description and have removed this sentence.*

Question: Figure 5d: How many CpTPS genes copies are there, and why only showing copy 1-20?

*Reply: In total, we have identified 52 TPS genes in the wintersweet genome, among which 20 were expressed in at least one of three developmental stages, with the fragments per kilobase of exon model per million reads mapped (FPKM) more than one. The remaining TPS genes showed a very low expression with an FPKM below 1 and were considered not expressed.*

*Among these 20 genes, there are 10 gene copies. Collinearity analyses of these 20 genes indicated that all the genes derived from tandem duplication (Figure5d).*

*CpTPS04 has three copies including CpTPS17, CpTPS18, and CpTPS19. CpTPS5 has three copies including CpTPS6, CpTPS12, and CpTPS13. CpTPS14 has one copy CpTPS15.*

Question: Figure 5, e & f: legend and figure are opposite.

*Reply: Thanks. We are sorry for this mistake. We have now adjusted the legend.*

Question: Figure 6a: What are the genes boxed by red and green boxes? And what are the non-boxed genes?

*Reply: The genes in green and red boxes were generated by WGD and tandem duplication events respectively. The non-boxed genes didn’t undergo these events.*

Question: Figure 6b, left panel: What are the grey bands connecting between chromosomes?

*Reply: The genes generated by whole-genome duplication events on different chromosomes were connected by both the grey and green bands. Now we have removed the grey bands only left the green bands connecting the duplicated benzenoids/phenylpropanoids biosynthesis genes. Now, we have changed the green bands to black bands in the revised Figure 6b.*

**Third round of review**

**Reviewer 2:**

Thank you for this revision. I have no major questions this time, just some wording issues:

Should gene names be italicized or not? For example, in the paragraph starting from line 627, some TPS are italicized but some not. Please be consistent throughout the manuscript.

Line 921: tick marks or thick marks?
